# Inverse-Probability-Weighted Wavelet Estimation of Regression Derivatives Under Missing-at-Random Responses for Stationary Ergodic Processes

**DOI:** 10.3390/e28080883

**Published:** 2026-08-05

**Authors:** Salim Bouzebda, Sultana Didi

**Affiliations:** 1 LMAC (Laboratory of Applied Mathematics of Compiègne), Université de Technologie de Compiègne, 60200 Compiègne, France; salim.bouzebda@utc.fr; 2 Department of Statistics and Operations Research, College of Sciences, Qassim University, P.O. Box 6688, Buraydah 51452, Saudi Arabia

**Keywords:** regression estimation, stationarity, ergodicity, rates of strong convergence, wavelet-based estimators, martingale differences, discrete-time, stochastic processes, time series, missing-at-random responses, 62G07, 62G08, 62G05, 62G20, 62H05, 60G42, 60G46

## Abstract

We consider the estimation of partial derivatives of multivariate regression-type functionals from incomplete observations generated by a discrete-time strictly stationary ergodic process. The response variable is subject to a missing-at-random (MAR) mechanism, whereas the covariates are fully observed. Building upon the complete-data wavelet methodology developed in Didi and Bouzebda (2025), we construct inverse-probability-weighted empirical wavelet estimators that compensate for the selection bias induced by missing responses. When the propensity score is unknown, a feasible estimator is obtained by replacing the oracle weights with a nonparametric Nadaraya–Watson estimator. The analysis is carried out under stationary ergodicity without imposing mixing assumptions. The estimation error is decomposed into three analytically distinct components: the deterministic multiresolution approximation error, the stochastic fluctuation of the oracle inverse-probability-weighted estimator, and the additional error arising from propensity score estimation. This decomposition makes it possible to isolate the respective effects of approximation, dependence, and missingness within a unified asymptotic framework. Under explicit assumptions on the multiresolution approximation, missingness mechanism, conditional density stabilization, moment conditions, and accuracy of the propensity estimator, we establish non-asymptotic integrated mean squared error bounds together with their asymptotic rates. We further prove almost-sure uniform consistency over compact subsets of the interior of the support and derive a pointwise central limit theorem for both the oracle and feasible estimators. The limiting variance explicitly reflects the information loss induced by inverse probability weighting, and for general orthogonal projection kernels is formulated under the corresponding dyadic-phase condition. The general methodology is specialized to the estimation of first- and second-order derivatives of ordinary regression functions. A finite-sample simulation study investigates the empirical behavior of the proposed estimators under stationary ergodic dependence and MAR missingness, examines the influence of both the wavelet resolution level and the propensity-score bandwidth, evaluates the finite-sample performance of the asymptotic confidence intervals, and compares the proposed procedure with oracle, complete-case, and competing nonparametric estimators. The numerical results are consistent with the theoretical analysis and illustrate the respective contributions of wavelet approximation, inverse probability weighting, and propensity score estimation to the overall estimation error. When the propensity score is identically equal to one, the proposed methodology reduces to the corresponding complete-data wavelet estimator.

## 1. Introduction

Nonparametric estimation of functional characteristics occupies a central position in modern mathematical statistics. In many applications, the object of inferential interest is not exhausted by the value of an unknown density or regression function itself; rather, its derivatives encode local geometry, rates of change, curvature, modal structure, and higher-order shape information, which arise naturally in problems of scientific interpretation as well as in the construction of statistical procedures. Kernel methods constitute one of the classical approaches to such questions because of their local character, analytical tractability, and broad applicability. Comprehensive treatments of kernel smoothing and related nonparametric procedures may be found in [1,2].

Density-derivative estimation provides a representative illustration. The gradient of a density identifies directions of local increase, while its Hessian and higher derivatives enter the analysis of curvature, modes, ridges, and other geometric features. The second derivative plays an important role in modality and topological inference [3], and density derivatives also appear in data-driven bandwidth selection [4]. In signal processing problems, the logarithmic derivative of a density, that is, the ratio between its derivative and the density itself, is central to filtering and interpolation procedures [5]. Gradient-based constructions are equally important in filament estimation for point-cloud data, with applications extending from medical imaging and remote sensing to seismology and cosmology [6]. Further motivations arise in Fisher information estimation, parameter estimation, regression analysis, and hypothesis testing [7]. Classical contributions to density-derivative estimation include [8,9,10,11,12].

Regression derivatives are no less fundamental. In a regression model, first-order derivatives quantify local marginal effects and monotonicity, while second- and higher-order derivatives describe curvature, acceleration, and interaction structure. Such quantities are often more informative than the regression level itself when the scientific question concerns change rather than magnitude. Applications include the analysis of human growth curves [13], the assessment of kidney function in lupus nephritis [14], and the processing of Raman spectra [15]. Derivative estimators also enter confidence interval construction [16], bandwidth selection [17], and the comparison of regression curves [18]. Kernel *M*-estimators for the first derivative of a regression function, together with extensions to higher orders, were investigated in [19]. Derivative information is also central to modal regression, which describes the conditional relationship between a response vector Y and a covariate vector X through the modes of the conditional distribution; see [20]. Foundational contributions to nonparametric regression estimation include [21,22,23,24].

Wavelet procedures provide a multiscale alternative to purely local kernel smoothing. Their appeal lies in the decomposition of a functional object into resolution-dependent components as well as in their simultaneous localization in space and scale. This structure separates approximation error from stochastic fluctuation, and permits regularity to be expressed through the decay of multiresolution coefficients, making wavelet methods naturally adapted to functions with spatially inhomogeneous smoothness, localized irregularities, or membership in Besov classes. A systematic account of wavelet methods in nonparametric function estimation is given in [25]. Related contributions include estimation of integrated squared density derivatives in the independent setting [26], extensions to negatively or positively associated sequences [27], and estimation of partial derivatives of multivariate densities under independence [28]. Wavelet derivative estimation under mixing-type dependence was studied in [29,30], while [31] considered partial-derivative estimation in the presence of additive noise.

The treatment of dependent observations introduces a further layer of difficulty. Independence is rarely tenable in applications involving time series, longitudinal measurements, repeated observations, dynamical systems, or stochastic processes. Much of the nonparametric literature replaces independence with mixing conditions, which are powerful but may be difficult to verify and can exclude stationary processes with weaker forms of dependence. The complete-data work in [32] developed a wavelet framework for estimating partial derivatives of multivariate regression-type functionals under discrete-time strictly stationary ergodic observations. Its analysis used conditional density stabilization and martingale approximation rather than strong mixing, and established integrated risk bounds, uniform convergence, and pointwise asymptotic normality.

The present paper takes [32] as its complete-data benchmark. Its contribution is not a new wavelet projection principle but an extension of that construction to the missing-response context. This includes the genuinely new analytical tasks of controlling the inverse-probability-weighted stochastic component and quantifying the additional substitution error produced when the propensity score is estimated nonparametrically. This distinction is important for accurately assessing the scope and novelty of the paper.

Missing responses are common in longitudinal surveys, clinical studies, sensor networks, administrative databases, and other observational systems. When the observation probability depends on recorded covariates, a complete-case analysis generally targets a selected subpopulation rather than the population of interest. Therefore, a principled procedure must account for the observation mechanism. The modern taxonomy of missingness mechanisms originates in [33], which distinguished missing completely at random, missing at random, and not missing at random according to the conditional relation between the missingness indicator and the full data.

The missing-at-random assumption allows for the probability of observing the response to depend on the covariates while requiring that, conditionally on those covariates, the missingness indicator contain no further information about the unobserved response. Its formal interpretation and inferential consequences have been analyzed in [34,35,36,37,38,39,40]. As discussed in [41], MAR-based procedures may offer substantial protection against the bias of naive complete-case methods or methods that implicitly assume missing completely at random.

A standard corrective device under MAR is inverse probability weighting, the foundations of which go back to [42]. The observed contribution is multiplied by the reciprocal of its conditional observation probability, commonly called the propensity score. Under MAR and positivity, the resulting compensation identity restores the complete-data target in expectation. Related developments concerning robustness, semiparametric efficiency, and doubly robust procedures may be found in [33,43,44,45].

We observe a strictly stationary ergodic sequence $(X_{i},\delta_{i}Y_{i},\delta_{i}),\;i=1,\ldots ,n,$ where $X_{i}\in R^{d}$ is fully observed, $Y_{i}\in R^{d^{\prime }}$ may be missing, and $\delta_{i}$ indicates whether the response is observed. For a measurable transformation $\rho :R^{d^{\prime }}\to R$, define

$$r\left(\rho ;x\right)=E\left[\rho \left(Y\right)|X=x\right]f_{X}\left(x\right).$$

The estimand is the partial derivative $\left(\partial^{\beta }r\right)\left(\rho ;x\right).$ This formulation encompasses ordinary regression-type functionals, distributional regression functionals, and derivative quantities relevant to modal and shape analysis.

The oracle construction inserts the inverse propensity weight into the complete-data empirical wavelet coefficients. Under MAR, positivity, and integrability, the weighted coefficient has the same expectation as its complete-data counterpart. The feasible estimator replaces the unknown propensity score with a Nadaraya–Watson estimator; consequently, the procedure contains two distinct smoothing mechanisms: the resolution level m(n), or equivalently the wavelet bandwidth $h_{n}=2^{-m(n)}$, controls multiresolution approximation and stochastic resolution, while the propensity bandwidth $\lambda_{n}$ controls the bias and variability of the estimated inverse weights. Their joint behavior cannot in general be reduced to two independent tuning problems.

The theoretical analysis is organized around the exact decomposition

totalerror=projectionbias+oracleIPWfluctuation+propensity-substitutionerror.

The projection term is governed by Besov approximation. The oracle stochastic term is analyzed through martingale differences, conditional density stabilization, Burkholder–Rosenthal inequalities, exponential inequalities for unbounded martingale arrays, and localization properties of derivative projection kernels. The feasible–oracle term requires uniform or mean-square control of the estimated propensity score at the normalization relevant to the theorem under consideration.

Strict stationarity and ergodicity alone do not yield the quantitative rates used below. Accordingly, the integrated-risk theorem assumes a coefficient-level mean-square stabilization condition; the uniform theorem invokes explicit conditional-moment kernel localization, truncation, bandwidth, and propensity rate hypotheses; and the central limit theorem requires local conditional density stabilization, kernel moment bounds, predictable-centering negligibility, projection-bias negligibility, and feasible–oracle equivalence. These conditions are stated theorem by theorem in Section 3 rather than being attributed to ergodicity itself.

The first principal result concerns integrated $L_{2}$-risk. For the oracle IPW estimator, we derive a general bound under a moment exponent ν>2 and a refined fourth-moment bound under ν≥4. The stochastic exponent is obtained from the coefficient-level variance calculation, and is balanced against the squared Besov projection bias. Conditions are then given under which the feasible estimator inherits the corresponding oracle order. The resulting rate is the one obtained from the displayed bias and stochastic bounds; no alternative exponent is asserted without a separate derivation.

The second result concerns uniform convergence on compact subsets of the interior of the support. The bound separately displays the martingale fluctuation, predictable conditional density approximation, and feasible–oracle error. This decomposition makes explicit how wavelet resolution, conditional-law stabilization, and propensity estimation jointly determine the uniform rate.

The third result is a pointwise central limit theorem. The limiting variance contains the inverse-propensity factor, meaning that it quantifies the first-order information loss caused by missing responses. For a general orthogonal projection kernel, the kernel energy term may depend on the dyadic phase $\vartheta_{n}=\frac{x}{h_{n}}-\left\lfloor \frac{x}{h_{n}}\right\rfloor .$ The theorem is consequently stated along sequences for which $\vartheta_{n}$ converges, unless the kernel energy is phase invariant. A full-sequence phase-zero variance is not asserted without one of these additional properties.

The general estimator is subsequently specialized to derivatives of an ordinary regression function. In the scalar case, the response-dependent numerator and its derivatives are estimated by inverse probability weighting, whereas the marginal density of the fully observed covariate is estimated from all covariate observations without weighting. First- and second-order regression-derivative estimators are then obtained through exact quotient identities. Their uniform consistency is established on compact subsets of the interior of the density-positive region, where the estimated denominator is asymptotically bounded away from zero. This application illustrates the reduction of the general construction when $d=d^{\prime }=1$; it does not by itself provide a separate empirical verification of all multivariate assertions.

The paper also contains a simulation study. The numerical investigation examines finite-sample bias, integrated error, the effect of missingness, the difference between oracle, feasible, and complete-case procedures, sensitivity to the wavelet resolution and propensity bandwidth, Gaussian approximation, confidence interval coverage, and comparison with a competing derivative estimator. The simulation evidence is necessarily conditional on the specified models, sample sizes, missingness mechanisms, tuning rules, and competitors. It supports the qualitative conclusions within those designs, but does not establish general finite-sample dominance, minimax optimality, or semiparametric efficiency. No real-data analysis is included in the present version, and claims of practical scope are kept commensurate with the evidence supplied.

The contribution may be summarized as follows: the paper provides a rigorous inverse-probability-weighted extension of a complete-data wavelet estimator to stationary ergodic observations with MAR responses; separates oracle and propensity-estimation errors at the levels of integrated risk, uniform convergence, and pointwise asymptotic normality; identifies the inverse propensity and dyadic-phase contributions to the limiting variance; and develops a scalar regression-derivative specialization together with finite-sample numerical evidence. This work should be read as a technically nontrivial extension of established wavelet and IPW principles rather than as the introduction of an entirely new estimation paradigm.

The limitations of the present framework should also be recorded at the outset. Positivity is assumed, and non-ignorable missingness is not covered. The feasible estimator is not doubly robust. The tuning parameters are deterministic in the theoretical results, and no theorem establishes optimality of a fully data-driven joint selector. The pointwise confidence interval is based on first-order asymptotics, and may require undersmoothing or explicit bias correction for accurate finite-sample coverage. The dependence conditions are expressed through conditional density and martingale array hypotheses that must be verified for the process under study. These issues motivate the extensions discussed in Section 6.

The rest of the paper is organized as follows: Section 2 introduces the Besov and multiresolution framework and defines the oracle and feasible MAR-adjusted wavelet estimators; Section 3 provides the structural assumptions, theorem-specific auxiliary conditions, integrated risk results, uniform convergence theorem, phase-correct pointwise central limit theorem, and confidence interval construction; Section 4 specializes the general method to first- and second-order regression derivatives; Section 5 presents the simulation study and the numerical comparison of the estimators; Section 6 summarizes the contribution, limitations, and open directions; Section 7 contains the proofs; and Section 8 records the intrinsic difference characterization, wavelet characterization, embedding facts, and projection inequalities used throughout the paper.

### Notation

Throughout the paper, *C* denotes a finite positive constant for which the value may change from line to line. The symbol $1_{A}$ denotes the indicator of an event or set *A*. For positive sequences ${\left\{a_{n}\right\}}_{n\geq 1}$ and ${\left\{b_{n}\right\}}_{n\geq 1}$, the relation

$$a_{n}=O\left(b_{n}\right)$$

means that $a_{n}\leq Cb_{n}$ for all sufficiently large *n*, while

$$a_{n}=o\left(b_{n}\right)$$

means that $a_{n}/b_{n}\to 0$. The symbols $O_{P}$ and $o_{P}$ denote the corresponding stochastic orders, and almost-sure orders are stated explicitly.

## 2. Mathematical Background

### 2.1. Besov Spaces

We first specify the functional-analytic framework used in the approximation arguments developed below. Besov spaces are particularly well adapted to wavelet procedures because their regularity may be characterized equivalently through the decay of multiresolution coefficients. This equivalence permits the deterministic approximation error of a wavelet projection to be expressed directly in terms of the smoothness and integrability indices of the target function. It is this approximation mechanism, rather than an informal appeal to multiscale localization, that underlies the bias bounds used in the subsequent asymptotic analysis.

Let s>0 and 1≤p,q≤∞. We denote by $B_{s,p,q}=B_{s,p,q}\left(R^{d}\right)$ the Besov space with smoothness index *s*, integrability index *p*, and fine index *q*. The parameter *s* quantifies regularity, *p* determines the underlying spatial integrability, and *q* governs the aggregation of contributions across resolution levels. The Besov scale is sufficiently flexible to accommodate spatially inhomogeneous regularity and localized irregularities that need not be described adequately by integer-order differentiability alone.

To prevent notational collision with the response dimension introduced below, the symbol *q* occurring in $B_{s,p,q}$ is used exclusively in this subsection as the Besov fine index. Whenever $Y\in R^{d^{\prime }}$ appears subsequently, *q* denotes the response dimension. Likewise, *p* in $B_{s,p,q}$ denotes only the Besov integrability index; moment orders appearing later in probabilistic inequalities will be denoted by distinct symbols.

Let ${\left\{V_{j}\right\}}_{j\in Z}$ be a multiresolution analysis of $L_{2}\left(R^{d}\right)$ with scaling space $V_{0}$, detail spaces $W_{j}$, scaling function ϕ, and associated wavelets $\psi_{i}$, $i=1,\ldots ,2^{d}-1$. We assume that the multiresolution analysis is *r*-regular and that 0<s<r. For $f\in L_{p}\left(R^{d}\right)$, the wavelet characterization of Meyer [46] yields that $f\in B_{s,p,q}$ if and only if the multiresolution quantity

$$J_{s,p,q}\left(f\right)={∥P_{V_{0}}f∥}_{L_{p}}+{\left(\sum_{j>0}{\left[2^{js}{∥P_{W_{j}}f∥}_{L_{p}}\right]}^{q}\right)}^{1/q}$$

is finite, with the usual replacement of the $\ell_{q}$-sum by a supremum when q=∞. Here, $P_{V_{0}}$ and $P_{W_{j}}$ denote the corresponding orthogonal projection operators. Equivalently, if 

$$a_{0,k}=\int_{R^{d}}f\left(u\right)\phi_{0,k}\left(u\right)\;du,\;b_{i,j,k}=\int_{R^{d}}f\left(u\right)\psi_{i,j,k}\left(u\right)\;du$$

are the scaling and wavelet coefficients of *f*, respectively, then $f\in B_{s,p,q}$ if and only if

$$J_{s,p,q}^{\prime }\left(f\right)={∥a_{0,\cdot }∥}_{\ell_{p}}+{\left(\sum_{j>0}{\left[2^{j\{s+d(1/2-1/p)\}}{∥b_{j,\cdot }∥}_{\ell_{p}}\right]}^{q}\right)}^{1/q}<\infty ,$$

again with the standard supremum convention when q=∞, where

$$∥b_{j,\cdot }{∥}_{\ell_{p}}={\left(\sum_{i=1}^{2^{d}-1}\sum_{k\in Z^{d}}{\left|b_{i,j,k}\right|}^{p}\right)}^{1/p}.$$

For p=∞, the corresponding $\ell_{\infty }$-norm is understood in the usual sense.

The preceding coefficient characterization provides the precise approximation device required later. More specifically, the regularity encoded by the Besov norm controls the decay of the detail coefficients as the resolution level increases. Hence, replacing *f* by its projection onto $V_{m}$ amounts to discarding the detail components above level *m*, and the resulting approximation error is bounded through the corresponding Besov coefficient tail. In particular, for $f\in B_{s,p,q}$, one obtains bounds of the form

$$∥f-P_{V_{m}}f∥≲2^{-m\;\alpha (s,p,d)}{∥f∥}_{B_{s,p,q}},$$

where the exponent α(s,p,d) and the admissible range of (s,p,q) depend on the norm in which the approximation error is evaluated. Accordingly, no norm-independent rate is asserted at this stage; the specific embedding and approximation inequalities required for each theorem are stated and invoked under their corresponding hypotheses.

The Besov scale contains several standard regularity classes as particular cases. For example, $H^{s}\left(R^{d}\right)=B_{s,2,2}\left(R^{d}\right)$, with equivalence of norms, while the Hölder–Lipschitz–Zygmund scale is represented by $B_{s,\infty ,\infty }\left(R^{d}\right)$, subject to the usual interpretation at integer smoothness indices. Therefore, the Besov formulation encompasses the familiar Sobolev and Hölder regimes while retaining the finer resolution-level control needed for wavelet approximation.

In what follows, Besov assumptions are imposed on the marginal density, on the conditional-density quantities entering the ergodic approximation conditions, and on the target derivative functional $\left(\partial^{\beta }r\right)\left(\rho ;\cdot \right)$. Their role is explicit: they provide the approximation and embedding properties needed to control the projection bias and to guarantee boundedness or continuity where required. In particular, whenever an argument uses the embedding $B_{s,p,q}\left(R^{d}\right)↪L_{\infty }\left(R^{d}\right)$, the dimension-dependent condition s>d/p is required; the weaker condition s>1/p is in general not sufficient when d>1.

### 2.2. Linear Wavelets Estimator Under Missing Responses

We now introduce the wavelet estimator under missing responses. Throughout this subsection, the wavelet notation follows [46]. Let ${\left\{V_{j}\right\}}_{j\in Z}$ be a multiresolution analysis of $L_{2}\left(R^{d}\right)$. Denote the scaling function by ϕ and the associated orthogonal wavelet by ψ, with both assumed to be *r*-regular (r≥1) and compactly supported in the hypercube ${\left[-L,L\right]}^{d}$ for some L>0. For every integer *j* and every $k\in Z^{d}$, define

$$\phi_{j,k}\left(x\right)=2^{jd/2}\phi \left(2^{j}x-k\right).$$

The family ${\left\{\phi_{j,k}\right\}}_{k\in Z^{d}}$ is an orthonormal basis of $V_{j}$. Moreover, we assume that every partial derivative of ϕ having total order at most *r* satisfies the stated rapid-decay bound: for every integer i>0, there exists a constant $A_{i}>0$, such that

(1) $$\left|\left(\partial^{\beta }\phi \right)\left(x\right)\right|\leq \frac{A_{i}}{{\left(1+∥x∥\right)}^{i}},\;\left|\beta \right|\leq r.$$

There exist $2^{d}-1$ companion wavelets $\psi_{1},\ldots ,\psi_{2^{d}-1}$, and the system

$$\left\{\psi_{\ell ,j,k}\left(x\right)=2^{jd/2}\psi_{\ell }\left(2^{j}x-k\right):\ell =1,\ldots ,2^{d}-1,\;k\in Z^{d}\right\}$$

generates the detail spaces in the multiresolution decomposition of $L_{2}\left(R^{d}\right)$.

We next specify the observation scheme. Let

$$(X_{i},Y_{i},\delta_{i}),\;i=1,\ldots ,n$$

be observations from a strictly stationary ergodic process, where $X_{i}\in R^{d}$ is fully observed, $Y_{i}\in R^{d^{\prime }}$ is the response vector, and 

$$\delta_{i}=1_{\left\{Y_{i}\;\mathrm{is}\;\mathrm{observed}\right\}}=\left\{\begin{array}{cc} 1, & \mathrm{if}\;Y_{i}\;\mathrm{is}\;\mathrm{observed},\\ 0, & \mathrm{otherwise}. \end{array}\right.$$

The response is assumed to be missing at random (MAR), in the sense that

(2) $$\pi \left(X_{i}\right)=P\left(\delta_{i}=1|X_{i},Y_{i}\right)=P\left(\delta_{i}=1|X_{i}\right),$$

so that $\delta_{i}$ and $Y_{i}$ are conditionally independent given $X_{i}$. The propensity score is further assumed to satisfy the positivity condition

(3) $$0<\pi_{0}\leq \pi \left(x\right)\leq 1,\;x\in J$$

for some constant $\pi_{0}>0$. Positivity ensures that the inverse weights are well-defined and uniformly bounded on the region on which the estimator is studied; without such a lower bound, the inverse-probability-weighted coefficients need not possess the moment properties required below.

Let (X,Y) have joint density $f_{X,Y}$ on $J\times R^{d^{\prime }}$ and let

$$f_{X}\left(x\right)=\int_{R^{d^{\prime }}}f_{X,Y}\left(x,y\right)\;dy.$$

For a measurable function $\rho :R^{d^{\prime }}\to R$, define

$$r\left(\rho ;x\right)=E\left[\rho \left(Y\right)|X=x\right]\;f_{X}\left(x\right)=\int_{R^{d^{\prime }}}\rho \left(y\right)f_{X,Y}\left(x,y\right)\;dy.$$

Whenever differentiation under the integral sign is justified by the regularity and integrability assumptions imposed below, the estimand is

$$\left(\partial^{\beta }r\right)\left(\rho ;x\right)=\int_{R^{d^{\prime }}}\rho \left(y\right)\left(\partial^{\beta }f_{X,Y}\right)\left(x,y\right)\;dy,$$

where

$$\partial^{\beta }=\frac{\partial^{\left|\beta \right|}}{\partial x_{1}^{\beta_{1}}\ldots \partial x_{d}^{\beta_{d}}},\;\left|\beta \right|=\beta_{1}+\cdots +\beta_{d}.$$

Thus, the displayed integral representation is not taken as a purely formal identity; it is used only under conditions ensuring the existence of the indicated derivative and the validity of the interchange between differentiation and integration.

Assume that $\left(\partial^{\beta }r\right)\left(\rho ;\cdot \right)\in L_{2}\left(R^{d}\right)$. To standardize the resolution notation throughout the paper, we write τ=m(n) henceforth, where m(n) is integer-valued and m(n)→∞; equivalently, $h_{n}=2^{-m(n)}$. As such, the symbols τ, *m*, and m(n) do not denote distinct tuning parameters. Then, the orthogonal projection of $\left(\partial^{\beta }r\right)\left(\rho ;\cdot \right)$ onto $V_{\tau }$ is

(4) $$P_{V_{\tau }}\left[\left(\partial^{\beta }r\right)\left(\rho ;\cdot \right)\right]\left(x\right)=\sum_{k\in Z^{d}}a_{\tau ,k}\phi_{\tau ,k}\left(x\right),$$

with coefficients

$$a_{\tau ,k}=\int_{R^{d}}\left(\partial^{\beta }r\right)\left(\rho ;u\right)\phi_{\tau ,k}\left(u\right)\;du={\left(-1\right)}^{\left|\beta \right|}\int_{R^{d}}r\left(\rho ;u\right)\left(\partial^{\beta }\phi_{\tau ,k}\right)\left(u\right)\;du.$$

The second equality follows via integration by parts, provided that the weak derivative of the required order exists and the boundary term vanishes; these requirements are ensured, for example, by the regularity and support conditions imposed in the sequel. The projection operator on the left-hand side of (4) is essential: at a finite resolution τ, the series represents the $V_{\tau }$-projection of the target and equals the target itself only when the latter already belongs to $V_{\tau }$.

If the responses were fully observed, the natural empirical counterpart of the coefficient would be

$${\hat{a}}_{\tau ,k}^{\;\mathrm{full}}=\frac{{\left(-1\right)}^{\left|\beta \right|}}{n}\sum_{i=1}^{n}\rho \left(Y_{i}\right)\left(\partial^{\beta }\phi_{\tau ,k}\right)\left(X_{i}\right).$$

With missing responses, the corresponding complete-case coefficient is

$${\hat{a}}_{\tau ,k}^{\;\mathrm{cc}}=\frac{{\left(-1\right)}^{\left|\beta \right|}}{\sum_{j=1}^{n}\delta_{j}}\sum_{i=1}^{n}\delta_{i}\rho \left(Y_{i}\right)\left(\partial^{\beta }\phi_{\tau ,k}\right)\left(X_{i}\right).$$

Under MAR, this complete-case normalization does not in general recover the full-data coefficient, as the conditional observation probability may vary with the covariate; it is unbiased only under additional restrictions, such as a covariate-independent observation probability. To correct the resulting selection distortion, we use inverse probability weighting. If π(·) is known, then the IPW pseudo-coefficient is

(5) $${\tilde{a}}_{\tau ,k}^{\;\mathrm{ipw}}=\frac{{\left(-1\right)}^{\left|\beta \right|}}{n}\sum_{i=1}^{n}\frac{\delta_{i}}{\pi (X_{i})}\rho \left(Y_{i}\right)\left(\partial^{\beta }\phi_{\tau ,k}\right)\left(X_{i}\right).$$

Indeed, by (2) and the tower property,

$$E\;\left[\frac{\delta_{i}}{\pi (X_{i})}\rho \left(Y_{i}\right)\left(\partial^{\beta }\phi_{\tau ,k}\right)\left(X_{i}\right)\right]=E\;\left[\rho \left(Y_{i}\right)\left(\partial^{\beta }\phi_{\tau ,k}\right)\left(X_{i}\right)\right]$$

whenever either side is absolutely integrable. This compensation identity is the precise reason that the oracle IPW coefficient targets the same population coefficient as its full-data counterpart. Accordingly, the present construction should be understood as an inverse-probability-weighted extension of the complete-data wavelet coefficient rather than as a distinct projection principle. The new analytical issue is the control of the inverse-weighted stochastic term, and for the feasible procedure, of the error induced by estimating the propensity score.

In applications, π(·) is typically unknown. We estimate it by a Nadaraya–Watson estimator. Let $K^{*}:R^{d}\to \left[0,\infty \right)$ be a kernel and let $\lambda_{n}>0$ be the propensity score bandwidth, which is distinct from the wavelet bandwidth $h_{n}=2^{-m(n)}$. Put

$$K_{\lambda_{n}}^{*}\left(u\right)=\lambda_{n}^{-d}K^{*}\left(\lambda_{n}^{-1}u\right)$$

and define

(6) $${\hat{\pi }}_{i}={\hat{\pi }}_{n}\left(X_{i}\right)=\frac{\sum_{j=1}^{n}\delta_{j}K_{\lambda_{n}}^{*}\left(X_{i}-X_{j}\right)}{\sum_{j=1}^{n}K_{\lambda_{n}}^{*}\left(X_{i}-X_{j}\right)}.$$

This definition is meaningful in the event that the denominator is nonzero. The assumptions imposed in Section 3 are intended to guarantee uniform consistency on the relevant compact set and to ensure, together with positivity, that ${\hat{\pi }}_{n}$ is eventually bounded away from zero with the stated mode of convergence. No such stability is inferred from the formula alone. The feasible MAR-adjusted coefficient is

(7) $${\hat{a}}_{\tau ,k}^{\;\mathrm{mar}}=\frac{{\left(-1\right)}^{\left|\beta \right|}}{n}\sum_{i=1}^{n}\frac{\delta_{i}}{{\hat{\pi }}_{i}}\rho \left(Y_{i}\right)\left(\partial^{\beta }\phi_{\tau ,k}\right)\left(X_{i}\right).$$

Consequently, the feasible linear wavelet estimator of $\left(\partial^{\beta }r\right)\left(\rho ;\cdot \right)$ under MAR is

(8) $${\hat{\left(\partial^{\beta }r\right)}}_{n}^{\;\mathrm{mar}}\left(\rho ;x\right)=\sum_{k\in Z^{d}}{\hat{a}}_{\tau ,k}^{\;\mathrm{mar}}\phi_{\tau ,k}\left(x\right).$$

The dependence of this estimator on two smoothing scales is structural: m(n), or equivalently $h_{n}$, controls the wavelet approximation and stochastic resolution, whereas $\lambda_{n}$ controls the accuracy of the estimated inverse-probability weights. The theoretical results must impose conditions on their joint asymptotic behavior; neither tuning parameter can in general be analyzed independently of the other.

Equivalently, using the wavelet projection kernel

(9) $$K\left(u,v\right)=\sum_{k\in Z^{d}}\phi \left(u-k\right)\phi \left(v-k\right)$$

and its derivative version

(10) $$K^{\left(\beta \right)}\left(u,v\right)=\sum_{k\in Z^{d}}\phi \left(u-k\right)\left(\partial_{v}^{\beta }\phi \right)\left(v-k\right),$$

the estimator (8) can be written in kernel form as

(11) $${\hat{\left(\partial^{\beta }r\right)}}_{n}^{\;\mathrm{mar}}\left(\rho ;x\right)=\frac{{\left(-1\right)}^{\left|\beta \right|}}{nh_{n}^{d+|\beta |}}\sum_{i=1}^{n}\frac{\delta_{i}}{{\hat{\pi }}_{i}}\rho \left(Y_{i}\right)K^{\left(\beta \right)}\left(\frac{x}{h_{n}},\frac{X_{i}}{h_{n}}\right),\;h_{n}=2^{-m(n)}.$$

This representation follows from the scaling relation for $\phi_{\tau ,k}$, the definition τ=m(n), and the finite-overlap properties inherited from compact support. It is an alternative representation of the same linear projection estimator, and does not introduce an additional kernel smoothing procedure.

The corresponding pseudo-estimator, obtained when the true propensity score is known, is

(12) $${\tilde{\left(\partial^{\beta }r\right)}}_{n}^{\;\mathrm{ipw}}\left(\rho ;x\right)=\frac{{\left(-1\right)}^{\left|\beta \right|}}{nh_{n}^{d+|\beta |}}\sum_{i=1}^{n}\frac{\delta_{i}}{\pi (X_{i})}\rho \left(Y_{i}\right)K^{\left(\beta \right)}\left(\frac{x}{h_{n}},\frac{X_{i}}{h_{n}}\right).$$

The estimator (11) is the feasible procedure, whereas (12) serves as the oracle intermediate object in the theoretical analysis. Under (2) and (3), and the stated integrability conditions, the oracle weighting restores the full-data coefficient in expectation. Therefore, the additional task for the feasible estimator is to prove that replacing π by ${\hat{\pi }}_{n}$ is asymptotically negligible at the scale relevant to each risk bound or limit theorem; this conclusion is not automatic, and is established only under the explicit rate conditions stated later.

## 3. Assumptions and Main Results

We now formulate the assumptions and principal asymptotic results for the MAR-adjusted wavelet estimator introduced in (11). The hypotheses are organized according to their logically distinct functions: (C) controls conditional law stabilization and wavelet approximation; (M) specifies the missingness mechanism and the propensity estimator; (N) governs response moments and conditional regression structure; and (A) records the additional martingale array requirements used only for pointwise asymptotic normality. This separation is substantive, with no quantitative rate inferred from ergodicity alone and no condition concerning estimation of the propensity score imposed on the oracle estimator.

The inverse-probability-weighting identity restores the complete-data target under MAR, whereas the feasible estimator replaces the unknown propensity score with its Nadaraya–Watson estimate. Thus, the resulting analysis must distinguish three errors: the wavelet projection bias, the stochastic fluctuation of the oracle IPW estimator, and the additional substitution error caused by estimating the propensity score.

Let

$$F_{n}=\sigma \left((X_{i},Y_{i},\delta_{i}):1\leq i\leq n\right)$$

and define

$$G_{n}=\sigma \left(\left(X_{k},Y_{k},\delta_{k}\right):1\leq k\leq n;\;X_{n+1}\right).$$

For every i≥1, let $f^{F_{i-1}}\left(\cdot \right)$ denote the conditional density of $X_{i}$ given $F_{i-1}$. When needed, $g^{F_{i-1}}\left(\cdot \right)$ denotes the conditional density of $Y_{i}$ given $F_{i-1}$.

All σ-fields are understood to be completed by the P-null sets. For the conditional identities in (N.2), we interpret

$$G_{i-1}=F_{i-1}\vee \sigma \left(X_{i}\right),$$

which agrees with the displayed definition of $G_{n}$ after the index shift n=i−1. We assume that the regular conditional laws admit jointly measurable versions of the densities $\left(\omega ,x\right)\mapsto f^{F_{i-1}}\left(\omega ,x\right)$ and, when used, $\left(\omega ,y\right)\mapsto g^{F_{i-1}}\left(\omega ,y\right)$. Whenever a spatial supremum is taken, these versions are assumed to be spatially continuous on the relevant compact set, so the supremum is a measurable random variable. We write

$$m_{\rho }\left(x\right)=E\left[\rho \left(Y_{i}\right)|X_{i}=x\right],\;m_{\rho ,\eta }\left(x\right)=E[|\rho \left(Y_{i}\right){|}^{\eta }|X_{i}=x].$$

The assumptions used in the respective results are given below; each theorem invokes only the subset explicitly listed in its statement.

**(C.1)**  For every x∈S,
  $$\frac{1}{n}\sum_{i=1}^{n}f^{F_{i-1}}\left(x\right)⟶f_{X}\left(x\right),\;n\to \infty ,$$
  both almost surely and in $L^{2}$.
**(C.2)**  Moreover,
  $$\underset{x\in R^{d}}{\sup}\left|\frac{1}{n}\sum_{i=1}^{n}f^{F_{i-1}}\left(x\right)-f_{X}\left(x\right)\right|⟶0,\;n\to \infty ,$$
  both almost surely and in $L^{2}$.
**(C.$2^{♯}$)**  There exists a constant $C_{\mathrm{st}}<\infty$ such that
  (13) $$E\left[\underset{x\in R^{d}}{\sup}{\left|\frac{1}{n}\sum_{i=1}^{n}f^{F_{i-1}}\left(x\right)-f_{X}\left(x\right)\right|}^{2}\right]\leq \frac{C_{\mathrm{st}}}{n},\;n\geq 1.$$
  This quantitative strengthening is invoked only in results asserting an explicit $n^{-1}$ mean-square rate. None of strict stationarity, ergodicity, (C.1), or (C.2) alone imply (13).
**(C.3)** 
  (i) The multiresolution analysis is *r*-regular.
  (ii) The target function $\left(\partial^{\beta }r\right)\left(\rho ;\cdot \right)$ belongs to the Besov space $B_{s,p,q}$ for some 0<s<r and 1≤p,q≤∞.
**(C.4)** 
  (i) The marginal density $f_{X}$ belongs to $B_{s,p,q}$ for some 0<s<r and 1≤p,q≤∞.
  (ii) The conditional densities $f^{F_{i-1}}\left(\cdot \right)$ belong to $B_{s,p,q}$ for the same range of parameters.
**(C.5)**  The Besov parameters in (C.4)(i) satisfy s>d/p. Consequently, the Besov embedding theorem gives $f_{X}\in L^{\infty }\left(R^{d}\right)$. Moreover, there exists a compact rectangle $J\subset R^{d}$ such that
  $$\mathrm{supp}\left(f_{X}\right)\cup \mathrm{supp}\left(r\left(\rho ;\cdot \right)\right)\subset J.$$
  The compact-support condition is used when the global integrated risk is reduced to a finite sum of coefficient risks. It may be replaced by a uniformly summable tail condition on the coefficient family.
**(M.1)**  The missingness mechanism is missing-at-random:
  $$\pi \left(X_{i}\right)=P\left(\delta_{i}=1|X_{i},Y_{i}\right)=P\left(\delta_{i}=1|X_{i}\right).$$
  Equivalently, conditionally on $X_{i}$, the indicator $\delta_{i}$ is independent of $Y_{i}$.
**(M.2)**  The propensity score satisfies the positivity condition
  $$0<\pi_{0}\leq \pi \left(x\right)\leq 1,\;x\in J$$
  for some constant $\pi_{0}>0$.
**(M.3)**  The propensity score π(·) is continuous on J and satisfies a Hölder condition: there exist constants $\gamma_{\pi }>0$ and $C_{\pi }>0$ such that
  $$\left|\pi \left(u\right)-\pi \left(v\right)\right|\leq C_{\pi }{∥u-v∥}^{\gamma_{\pi }},\;u,v\in J.$$
**(M.4)**  The estimator ${\hat{\pi }}_{n}$ defined in (6) is uniformly consistent on J. More precisely, there exists a deterministic sequence $a_{n}\to 0$ such that
  $$\underset{x\in J}{\sup}\left|{\hat{\pi }}_{n}\left(x\right)-\pi \left(x\right)\right|=O\left(a_{n}\right)\;a.s.$$
  In particular, for all sufficiently large *n*,
  $$\underset{x\in J}{\inf}{\hat{\pi }}_{n}\left(x\right)\geq \pi_{0}/2\;a.s.$$
  For the Nadaraya–Watson estimator in (6), a typical choice is
  $$a_{n}={\left(\frac{\log n}{n\lambda_{n}^{d}}\right)}^{1/2}+\lambda_{n}^{\gamma_{\pi }},$$
  where $\lambda_{n}$ is the bandwidth used to estimate the propensity score.
  For every assertion formulated in mean square for the feasible estimator, we also assume
  (14) $$E\left[\underset{x\in J}{\sup}{\left|{\hat{\pi }}_{n}\left(x\right)-\pi \left(x\right)\right|}^{2}\right]=O\left(a_{n}^{2}\right).$$
  Almost-sure uniform consistency does not by itself imply (14).
**(N.0)**  For some ν>2,
  $$E[|\rho \left(Y_{1}\right){|}^{\nu }]<\infty .$$
  This is the baseline moment hypothesis. Every argument requiring a fourth moment, including the fourth-order risk bound, is invoked only under the explicitly strengthened condition ν≥4. Thus, the paper does not infer $E|\rho \left(Y_{1}\right){|}^{4}<\infty$ from the weaker assumption ν>2.
**(N.1)**  On the set $\left\{y\in R^{d^{\prime }}:g\left(y\right)>0\right\}$,
  $$\underset{y:g(y)>0}{\sup}\left|\frac{1}{ng(y)}\sum_{i=1}^{n}g^{F_{i-1}}\left(y\right)-1\right|⟶0,\;n\to \infty ,$$
  both almost surely and in $L^{2}$.
  The ratio in (N.1) is understood only on the set {y:g(y)>0}. An equivalent and technically safer formulation is to impose the corresponding convergence on $n^{-1}\sum_{i=1}^{n}g^{F_{i-1}}-g$ without division by *g*.
**(N.2)** 
  (i) For every i≥1,
    $$E\left[\rho \left(Y_{i}\right)|G_{i-1}\right]=E\left[\rho \left(Y_{i}\right)|X_{i}\right]=m_{\rho }\left(X_{i}\right).$$
  (ii) For every η≤2,
    $$E[|\rho \left(Y_{i}\right){|}^{\eta }|G_{i-1}]=E[|\rho \left(Y_{i}\right){|}^{\eta }|X_{i}]=m_{\rho ,\eta }\left(X_{i}\right),$$
    and $m_{\rho ,\eta }$ is continuous on J.
**(N.3)** 
  (i) There exists a constant $C_{m}>0$ such that
    $$\underset{x\in J}{\sup}\left|m_{\rho }\left(x\right)\right|\leq C_{m}.$$
  (ii) The regression function $m_{\rho }$ satisfies a Hölder condition: there exist constants β>0 and $c_{3}>0$ such that
    $$|m_{\rho }\left(u\right)-m_{\rho }\left(v\right)|\leq c_{3}{∥u-v∥}^{\beta },\;u,v\in R^{d}.$$
  (iii) The function $m_{\rho ,\eta }$ satisfies a Hölder condition: there exist constants $\beta_{1}>0$ and $c_{4}>0$ such that
    $$|m_{\rho ,\eta }\left(u\right)-m_{\rho ,\eta }\left(v\right)|\leq c_{4}{∥u-v∥}^{\beta_{1}},\;u,v\in R^{d}.$$
**(C.6)**  For the deterministic resolution sequence m=m(n), define
  $$D_{m,k}\left(x\right)=\left(\partial^{\beta }\phi_{m,k}\right)\left(x\right).$$
  There exists a constant $C_{\mathrm{coef}}<\infty$, independent of *n*, *m*, and k, such that
  $$\underset{k\in Z^{d}}{\sup}E\left[{\left|\frac{1}{n}\sum_{i=1}^{n}\int_{R^{d}}m_{\rho }\left(x\right)D_{m,k}\left(x\right)\left\{f^{F_{i-1}}\left(x\right)-f_{X}\left(x\right)\right\}\;dx\right|}^{2}\right]\leq C_{\mathrm{coef}}\frac{2^{2m(d/\nu +|\beta |)}}{n}.$$
  Condition (C.6) is imposed only for the integrated-risk result. It is the coefficient-level stabilization estimate actually used in its proof, and as such is weaker than a uniform $L^{2}$ rate for the entire conditional density field.

The conditions (C.1)–(C.6), (M.1)–(M.4), and (N.0)–(N.3) are the primitive assumptions of the section. They are not cumulative; each theorem invokes only the subset stated in its hypotheses. Maximal inequalities, predictable quadratic variation, Lindeberg negligibility, projection bias control, and feasible–oracle equivalence are theorem-specific asymptotic requirements and are stated directly in the corresponding theorem.

**Lemma** **1.** *Assume* (N.0)* and |β|≤r. Then, for every deterministic m∈N and every $k\in Z^{d}$,*

$$E\;\left[|\rho \left(Y_{1}\right)|\left|\left(\partial^{\beta }\phi_{m,k}\right)\left(X_{1}\right)\right|\right]<\infty$$

*and*

$$E\;\left[|\rho \left(Y_{1}\right){|}^{2}{\left|\left(\partial^{\beta }\phi_{m,k}\right)\left(X_{1}\right)\right|}^{2}\right]<\infty .$$

**Proof.**  Since ν>2, both $E|\rho (Y_{1})|$ and $E|\rho \left(Y_{1}\right){|}^{2}$ are finite. For fixed *m* and k, the derivative $\partial^{\beta }\phi_{m,k}$ is bounded by *r*-regularity. The assertions follow.    □

**Lemma** **2.** *Assume that the scaling function is compactly supported and r-regular, with |β|≤r. Then, for every η>0,*

$$\underset{\theta \in {\left[0,1\right]}^{d}}{\sup}\int_{R^{d}}{\left|K^{\left(\beta \right)}\left(\theta ,\theta +v\right)\right|}^{2+\eta }\;dv<\infty .$$

*In particular, the corresponding uniform $L^{2}$ bound holds. Moreover, for almost every v, the map*

$$\theta ⟼K^{\left(\beta \right)}\left(\theta ,\theta +v\right)$$

*is continuous on ${\left[0,1\right]}^{d}$, and the integrable envelope can be chosen independently of **θ**.*

**Proof.**  Compact support implies that only finitely many terms contribute to the series defining $K^{\left(\beta \right)}$, with the number of nonzero terms bounded uniformly in θ. The *r*-regularity of ϕ gives uniform boundedness and continuity of the contributing factors. As a function of v, the kernel is supported on a fixed compact set. The asserted integrability and continuity properties follow.    □

A convenient sufficient condition for (C.6) is

$$E\left[\underset{x\in J}{\sup}{\left|\frac{1}{n}\sum_{i=1}^{n}f^{F_{i-1}}\left(x\right)-f_{X}\left(x\right)\right|}^{2}\right]\leq \frac{C}{n}.$$

Under (N.3)(i), compact support, and |β|≤r, this stronger uniform condition implies (C.6) through the scaling relation ${∥D_{m,k}∥}_{1}≍2^{m(|\beta |-d/2)}$. Therefore, it is sufficient but not part of the minimal theorem statement.

The MAR condition implies the following compensation identity, while positivity guarantees that its inverse weights are well defined and uniformly bounded on J:

(15) $$E\left[\frac{\delta_{i}}{\pi (X_{i})}\rho \left(Y_{i}\right)\varphi \left(X_{i}\right)\right]=E\left[\rho \left(Y_{i}\right)\varphi \left(X_{i}\right)\right]$$

for every integrable measurable function φ. This identity is the main device that allows the wavelet estimator under MAR to target the same quantity as the complete-data estimator.

More precisely, (15) follows from

$$E\left[\left.\frac{\delta_{i}}{\pi (X_{i})}\right|X_{i},Y_{i}\right]=1\;a.s.,$$

and consequently remains valid for every φ such that either side is absolutely integrable.

**Remark** **1.** *The wavelet bandwidth $h=2^{-m}$ and propensity score bandwidth λ control distinct but interacting errors. The former determines the projection bias and stochastic fluctuation of the derivative estimator, whereas the latter determines the bias and variability of the estimated inverse-probability weights. Therefore, a practical selection procedure should choose the pair (h,λ) jointly rather than optimizing the two parameters in isolation.*

*For a stationary dependent sample, we use blocked cross-validation. Let $H_{n}$ and $\Lambda_{n}$ be finite grids of admissible wavelet and propensity score bandwidths, respectively, and partition {1,…,n} into B consecutive blocks $I_{1},\ldots ,I_{B}$. For each $\left(h,\lambda \right)\in H_{n}\times \Lambda_{n}$ and each block $I_{b}$, compute the Nadaraya–Watson propensity estimator ${\hat{\pi }}_{-b,\lambda }$ and the corresponding wavelet regression estimator ${\hat{m}}_{-b,h,\lambda }$ from the observations outside $I_{b}$. To preserve positivity in finite samples, put*

$${\hat{\pi }}_{-b,\lambda }^{\;\varepsilon }\left(x\right)=\max\left\{{\hat{\pi }}_{-b,\lambda }\left(x\right),\varepsilon_{n}\right\},\;\varepsilon_{n}↓0$$

*and define the blocked inverse-probability-weighted validation criterion*

$${\mathrm{BCV}}_{n}\left(h,\lambda \right)=\frac{1}{n}\sum_{b=1}^{B}\sum_{i\in I_{b}}\frac{\delta_{i}}{{\hat{\pi }}_{-b,\lambda }^{\;\varepsilon }\left(X_{i}\right)}{\left\{Y_{i}-{\hat{m}}_{-b,h,\lambda }\left(X_{i}\right)\right\}}^{2}.$$

*The jointly selected pair is*

$$\left({\hat{h}}_{n},{\hat{\lambda }}_{n}\right)\in \underset{\left(h,\lambda \right)\in H_{n}\times \Lambda_{n}}{arg min}{\mathrm{BCV}}_{n}\left(h,\lambda \right).$$

*When several pairs produce numerically indistinguishable criterion values, we select the smoother admissible pair, thereby avoiding unstable inverse weights and excessively small effective local sample sizes.*

*The candidate grids are restricted to the theoretical region*

$$h\to 0,\;\lambda \to 0,\;\frac{nh^{d}}{\log n}\to \infty ,\;\frac{n\lambda^{d}}{\log n}\to \infty ,$$

*and for asymptotic normality are restricted to pairs satisfying the additional undersmoothing and propensity negligibility conditions imposed in Theorem 3. Thus, blocked cross-validation provides a fully data-driven finite-sample choice within the set of bandwidth pairs allowed by the asymptotic theory. In the simulation study, the Silverman reference pair is retained as a transparent benchmark, while the separate sensitivity curves for h and λ display the interaction that motivates the joint criterion above.*

### 3.1. Discussion and Interpretation of the Assumptions

We collect here several comments on the assumptions introduced above. The purpose of this discussion is twofold. First, it identifies the precise probabilistic and analytic role played by each group of conditions. Second, it clarifies how the assumptions interact in the martingale approximation, the wavelet bias analysis, and the treatment of the missing-at-random mechanism. The conditions naturally fall into four classes: conditional-law stabilization assumptions, wavelet–Besov approximation assumptions, missing data assumptions, and moment and regression regularity assumptions.

#### 3.1.1. Conditional-Law Stabilization

Assumptions (C.1) and (C.2) are ergodic stabilization conditions imposed on the conditional densities of the covariate process. They replace the quantitative covariance inequalities that would ordinarily be derived from strong-mixing coefficients. More precisely, (C.1) requires that the Cesàro averages of the conditional densities converge pointwise to the stationary marginal density $f_{X}$, whereas (C.2) imposes the corresponding uniform convergence.

These assumptions are adapted to the martingale structure of the proofs. After conditioning on the past sigma field, the empirical terms are decomposed into a martingale difference component and a predictable remainder. The latter depends on averages of conditional densities, and its asymptotic negligibility follows directly from (C.1) or (C.2) depending on whether pointwise or uniform control is required. Thus, the analysis does not require an explicit rate of decay for the dependence coefficients; instead, it requires that the conditional law of a future covariate, averaged along the trajectory, asymptotically recover the stationary marginal law.

This property is best described as *ergodic stabilization*. It should not be interpreted as robustness in the classical statistical sense, as neither contamination, model mis-specification, nor resistance to outliers is at issue here. Rather, it is an asymptotic regularity property of the conditional distributions. It is precisely this stabilization that permits ergodicity to replace stronger quantitative dependence assumptions in the martingale approximation.

#### 3.1.2. Wavelet Regularity and Multiresolution Approximation

Assumptions (C.3) and (C.4) govern the analytic structure of the wavelet estimator. The *r*-regularity of the multiresolution analysis guarantees that the scaling function and the associated wavelets possess sufficient smoothness and localization for termwise differentiation of the wavelet expansion and for the derivative projection kernel estimates used throughout the proofs.

The Besov assumptions play a distinct and more substantive role. Let $P_{V_{m}}$ denote the projection onto the approximation space $V_{m}$. For the target derivative functional, the error may be decomposed as

$${\hat{\left(\partial^{\beta }r\right)}}_{n}-\left(\partial^{\beta }r\right)=\left\{{\hat{\left(\partial^{\beta }r\right)}}_{n}-P_{V_{m}}\left(\partial^{\beta }r\right)\right\}+\left\{P_{V_{m}}\left(\partial^{\beta }r\right)-\left(\partial^{\beta }r\right)\right\}.$$

The first term is stochastic, whereas the second is the deterministic *multiresolution projection bias*. Controlling this bias means proving that the error produced by truncating the wavelet expansion at resolution m=m(n) tends to zero at a rate compatible with the stochastic normalization of the estimator.

In particular, if 

$$\left(\partial^{\beta }r\right)\left(\rho ;\cdot \right)\in B_{s,p,q},\;s>\frac{d}{p},$$

then the wavelet approximation inequality used below yields

$${∥P_{V_{m}}\left(\partial^{\beta }r\right)\left(\rho ;\cdot \right)-\left(\partial^{\beta }r\right)\left(\rho ;\cdot \right)∥}_{\infty }\leq C\;2^{-m(s-d/p)}J_{s,p,q}\left(\left(\partial^{\beta }r\right)\left(\rho ;\cdot \right)\right).$$

Since $h_{n}=2^{-m(n)}$, the corresponding uniform approximation error is of order

$$h_{n}^{\;s-d/p}.$$

Accordingly, the Besov condition is not imposed merely to legitimate differentiation; by quantifying the decay of the high-frequency wavelet coefficients, it determines the order of the deterministic bias. This control is indispensable for the integrated risk bounds, the uniform convergence theorem, and the undersmoothing condition required for asymptotic normality.

The Besov assumptions imposed on $f_{X}$ and on the conditional densities $f^{F_{i-1}}$ serve a related but separate purpose. They provide uniform local approximation properties for the deterministic and predictable terms arising in the conditional-variance calculations. In particular, they justify replacing localized conditional density averages by their stationary limits at the spatial scale $h_{n}$.

#### 3.1.3. Missing-at-Random Structure and Inverse Probability Weighting

Assumptions (M.1) and (M.2) encode the missing-at-random mechanism. Assumption (M.1) states that, conditionally on the covariate vector, the missingness indicator contains no additional information about the response. Therefore, it yields the compensation identity

$$E\left[\frac{\delta_{i}}{\pi (X_{i})}\rho \left(Y_{i}\right)\;|\;X_{i}\right]=E\left[\rho \left(Y_{i}\right)\;|\;X_{i}\right],$$

which is the basic identification relation underlying the IPW construction.

The positivity assumption (M.2) is equally essential. By requiring that the propensity score be bounded away from zero, it prevents the inverse weights from becoming arbitrarily large. This guarantees that the weighted moments remain finite under the stated moment conditions and rules out regions of the covariate space in which the response is essentially unobservable. Without positivity, the variance of the IPW estimator may diverge and the target functional need not be identifiable from the observed sample.

Assumptions (M.3) and (M.4) are needed only for the feasible estimator, when π is unknown. The Hölder regularity in (M.3) controls the smoothing bias of the nonparametric propensity estimator, whereas (M.4) summarizes its uniform estimation error. For the Nadaraya–Watson estimator, the canonical decomposition is $a_{n},$ where the first term is stochastic and the second is the deterministic smoothing bias.

The additional conditions involving $a_{n}$ are scale separation conditions. Because they require the error induced by estimating π to be asymptotically smaller than the principal stochastic fluctuation of the wavelet estimator, they express neither an independent smoothness assumption nor an additional identifiability restriction; rather, their sole purpose is to ensure first-order equivalence between the feasible estimator and the pseudo-estimator constructed with the true propensity score. When π is known by design, $a_{n}=0$ and these restrictions disappear.

#### 3.1.4. Moment, Truncation, and Conditional Regression Assumptions

Assumption (N.0) imposes a moment condition on the transformed response ρ(Y). It is used at several distinct stages. It controls the contribution of large response values in the truncation argument, provides the integrability required by the martingale moment inequalities, and yields the Lindeberg-type negligibility needed for the central limit theorem. The threshold ν>2 is the natural minimal requirement for second-order arguments; stronger moment conditions may be invoked whenever fourth-order estimates are needed.

Assumption (N.1) is the response-density analogue of the uniform stabilization condition (C.2). It controls, uniformly over the relevant spatial domain, the predictable contribution of the truncated tail conditional on the past. Together with (N.0), it ensures that the large-response component is negligible at the scale of the uniform stochastic bound.

More precisely, the statement that the unboundedness of ρ(Y) does not affect the uniform convergence rate concerns the *asymptotic order* of the rate, not merely the fact of convergence. Under (N.0) and (N.1), the tail term generated by the truncation procedure is of smaller order than the leading stochastic term. Consequently, the estimator attains the same uniform rate as in the bounded response case, although the proof requires an additional truncation-and-remainder argument. If ρ is bounded or if the response itself is bounded, this part of the argument becomes unnecessary and (N.1) may be correspondingly weakened.

Assumption (N.2) specifies the conditional moment structure needed for the regression interpretation of the target and for the martingale decomposition. Its first part asserts that, conditionally on the current covariate, the past does not further alter the relevant conditional mean of the response transformation. This is substantially weaker than independence. Its role is to ensure that the empirical summands form a martingale difference array after subtracting the appropriate conditional expectation. The second part supplies the corresponding conditional second-moment identity, and thereby determines the limiting variance.

Assumption (N.3) imposes boundedness and local Hölder regularity on the regression-type functions entering the first- and second-order conditional moments. Boundedness prevents the localized kernel sums from acquiring uncontrolled deterministic contributions. Hölder regularity justifies the local replacements

$$m_{\rho }\left(X_{i}\right)=m_{\rho }\left(x\right)+O({∥X_{i}-x∥}^{\gamma }),$$

and similarly for $m_{\rho ,\eta }$, whenever $∥X_{i}-x∥=O(h_{n})$. These local expansions are used both in the proof of the uniform convergence theorem and in the identification of the pointwise asymptotic variance.

#### 3.1.5. Resolution, Bandwidth, and Truncation Scales

The restrictions on m(n), $h_{n}$, $\lambda_{n}$, and $T_{n}$ balance four quantities: stochastic fluctuation, wavelet projection bias, propensity score estimation error, and response tail truncation. The conditions

$$m\left(n\right)⟶\infty ,\;\frac{2^{dm(n)}\log n}{n}⟶0,$$

or equivalently

$$h_{n}⟶0,\;\frac{\log n}{nh_{n}^{d}}⟶0,$$

ensure that the approximation space becomes asymptotically dense and that the effective number of observations in a localization cell of volume $h_{n}^{d}$ tends to infinity.

The restriction involving $T_{n}$ ensures that the truncation threshold diverges sufficiently quickly for the truncated estimator to remain asymptotically equivalent to the original one and sufficiently slowly for martingale exponential inequalities to remain effective. The conditions involving $a_{n}$ guarantee that the feasible propensity score correction is of smaller order than the leading wavelet fluctuation. Finally, the undersmoothing condition imposed in the central limit theorem requires

normalizingscale×projectionbias⟶0,

so that the limiting distribution is centered at the target functional rather than at its finite-resolution projection.

#### 3.1.6. Summary

The assumptions separate the three structural difficulties of the problem. Temporal dependence is handled through conditional-law stabilization and martingale approximation; missingness is handled through the MAR compensation identity and positivity; and nonparametric approximation is handled through the interaction between Besov regularity and multiresolution projection. The feasible case introduces an additional second-order component, propensity score estimation, for which the contribution is required to be asymptotically negligible relative to the intrinsic fluctuation of the wavelet estimator.

This separation is central to the scope of the theory. It permits the complete-data wavelet methodology to be extended to missing-at-random responses under stationary ergodic dependence without imposing explicit strong mixing rates and without changing the first-order asymptotic behavior of the estimator when the propensity score is estimated with sufficient accuracy.

**Remark** **2.** *The use of strict stationarity and ergodic conditional density stabilization in place of quantitative strong mixing assumptions enlarges the class of admissible data-generating mechanisms, but should not be interpreted as yielding uniformly sharper finite-sample guarantees. The numerical form of the estimator is unchanged by this choice of dependence framework: for fixed smoothing parameters, the evaluation of ${\hat{\left(\partial^{\beta }r\right)}}_{n}^{\;\mathrm{mar}}$ requires the same propensity score computation and the same wavelet projection kernel sums irrespective of whether the observations are independent, mixing, or only stationary and ergodic; hence, the relaxation from mixing to ergodicity does not by itself impose an additional computational cost. The trade-off is probabilistic rather than algorithmic; under a specified mixing rate, covariance inequalities and exponential bounds may provide explicit dependence-adjusted constants, effective-sample-size interpretations, and finite-sample remainder estimates. Assumptions *(C.1)*–*(C.2)* instead require the Cesàro averages of the relevant conditional densities to stabilize toward their stationary marginals without postulating a universal rate for this stabilization. Accordingly, the asymptotic orders in the present paper remain valid under the stated bandwidth and stabilization conditions, but the finite-sample constants and the sample size at which the asymptotic approximation becomes accurate may depend strongly on the persistence of the underlying process. For highly persistent sequences, the effective information content of a sample of size n may be appreciably smaller than in a weakly dependent mixing model, even though the formal normalization and the leading bias–variance orders are unchanged. This distinction is illustrated in Section 5. The sensitivity analysis with respect to the autoregressive parameter and the comparison across several stationary ergodic mechanisms show a moderate increase in MISE as dependence becomes stronger, while preserving the qualitative ordering of the competing estimators. The reported CPU-time experiment further confirms that computational cost is governed primarily by n, the resolution level, and the propensity score smoothing step rather than by whether the dependence assumptions are formulated through mixing coefficients or through ergodic conditional density stabilization.*

**Lemma** **3.** *Under condition* (C.3)*, for s>d/p we have*

$$\begin{array}{cc} \underset{x\in R^{d}}{\sup}\left|E\left[{\tilde{\left(\partial^{\beta }r\right)}}_{n}^{\;\mathrm{ipw}}\left(\rho ;x\right)\right]-\left(\partial^{\beta }r\right)\left(\rho ;x\right)\right| & =\underset{x\in R^{d}}{\sup}\left|P_{V_{m}}\left(\partial^{\beta }r\right)\left(\rho ;x\right)-\left(\partial^{\beta }r\right)\left(\rho ;x\right)\right|\\ & \leq C\;2^{-(s-d/p)m(n)}J_{s,p,q}\left(\left(\partial^{\beta }r\right)\left(\rho ;\cdot \right)\right). \end{array}$$

For the integrated risk theorem, the relevant approximation statement is the $L^{2}$ bound:

(16) $${∥P_{V_{m}}\left(\partial^{\beta }r\right)\left(\rho ;\cdot \right)-\left(\partial^{\beta }r\right)\left(\rho ;\cdot \right)∥}_{2}^{2}\leq C\;2^{-2ms}{∥\left(\partial^{\beta }r\right)\left(\rho ;\cdot \right)∥}_{B_{s,2,q}}^{2}.$$

The uniform bound in Lemma 3 and the $L^{2}$ bound in (16) are distinct consequences of Besov regularity, and are not interchangeable. Define the wavelet projection kernel

$$K\left(u,v\right)=\sum_{k\in Z^{d}}\phi \left(u-k\right)\phi \left(v-k\right)$$

and its derivative version

$$K^{\left(\beta \right)}\left(u,v\right)=\sum_{k\in Z^{d}}\phi \left(u-k\right)\left(\partial_{v}^{\beta }\phi \right)\left(v-k\right).$$

As in the complete-data case, for |β|≤r,

(17) $$|K^{\left(\beta \right)}\left(u,v\right)|\leq \frac{C_{d+|\beta |+1}}{{(1+∥v-u∥}_{2}{)}^{d+|\beta |+1}}.$$

Furthermore,

$$\int_{R^{d}}{\left|K^{\left(\beta \right)}\left(v,u\right)\right|}^{j}\;dv\leq G_{j}\left(d\right),\;j\geq 1.$$

By the regularity of the kernel,

(18) $$|K^{\left(\beta \right)}\left(u,y\right)-K^{\left(\beta \right)}\left(v,y\right)|\leq d^{1/2}C_{2}{∥u-v∥}_{2}.$$

Therefore, the feasible MAR estimator can be written as

(19) $${\hat{\left(\partial^{\beta }r\right)}}_{n}^{\;\mathrm{mar}}\left(\rho ;x\right)=\frac{{\left(-1\right)}^{\left|\beta \right|}}{nh_{n}^{d+|\beta |}}\sum_{i=1}^{n}\frac{\delta_{i}}{{\hat{\pi }}_{n}\left(X_{i}\right)}\rho \left(Y_{i}\right)K^{\left(\beta \right)}\left(\frac{x}{h_{n}},\frac{X_{i}}{h_{n}}\right),\;h_{n}=2^{-m(n)}.$$

The associated pseudo-estimator, based on the true propensity score, is

(20) $${\tilde{\left(\partial^{\beta }r\right)}}_{n}^{\;\mathrm{ipw}}\left(\rho ;x\right)=\frac{{\left(-1\right)}^{\left|\beta \right|}}{nh_{n}^{d+|\beta |}}\sum_{i=1}^{n}\frac{\delta_{i}}{\pi (X_{i})}\rho \left(Y_{i}\right)K^{\left(\beta \right)}\left(\frac{x}{h_{n}},\frac{X_{i}}{h_{n}}\right).$$

### 3.2. Theorem-Specific Auxiliary Hypotheses

The assumptions in this subsection are local to the result in which they are invoked. Conditions (U.1)–(U.5) are used only for uniform convergence, while (CLT.1)–(CLT.8) are used only for pointwise asymptotic normality. They are separated from (C), (M), and (N) in order to distinguish structural assumptions on the model from asymptotic conditions on the resolution, truncation, conditional laws, and estimated propensity score.

#### 3.2.1. Conditions for Uniform Convergence

**(U.1)**  There exists a compact set D⊂int(J), and the derivative projection kernel satisfies the following for some finite constants $C_{K},C_{K,1}$:
  (21) $$\begin{array}{cc} \left|K^{\left(\beta \right)}\left(u,v\right)\right| & \leq \frac{C_{K}}{{\left(1+∥u-v∥\right)}^{d+|\beta |+1}}, \end{array}$$
  (22) $$\begin{array}{cc} \left|K^{\left(\beta \right)}\left(u,y\right)-K^{\left(\beta \right)}\left(v,y\right)\right| & \leq C_{K,1}∥u-v∥. \end{array}$$
  Moreover, for every j≥1,
  $$\underset{u\in R^{d}}{\sup}\int_{R^{d}}{\left|K^{\left(\beta \right)}\left(u,v\right)\right|}^{j}\;dv<\infty .$$
**(U.2)**  For some ν>2,
  $$\underset{i\geq 1}{\sup}{∥E\left[|\rho \left(Y_{i}\right){|}^{\nu }|G_{i-1}\right]∥}_{\infty }<\infty .$$
  This is stronger than the marginal moment condition (N.0) and is required for the conditional truncation and martingale inequalities used below.
**(U.3)**  The conditional densities are uniformly bounded:
  $$\underset{i\geq 1}{\sup}{∥f^{F_{i-1}}∥}_{\infty }<\infty \;a.s.$$
  Furthermore, with 
  $$\Delta_{n}\left(y\right)=\frac{1}{n}\sum_{i=1}^{n}f^{F_{i-1}}\left(y\right)-f_{X}\left(y\right),$$
  there exists a deterministic sequence $b_{n}↓0$ such that
  (23) $${∥\Delta_{n}∥}_{\infty }=O(b_{n})\;a.s.$$
**(U.4)**  The bandwidth and truncation sequences satisfy
  (24) $$\begin{array}{cc} h_{n} & ↓0,\;\frac{nh_{n}^{d+2|\beta |}}{\log n}⟶\infty , \end{array}$$
  (25) $$\begin{array}{cc} T_{n} & ↑\infty ,\;T_{n}{\left(\frac{\log n}{nh_{n}^{d}}\right)}^{1/2}⟶0, \end{array}$$
  (26) $$\begin{array}{cc} \sum_{n=1}^{\infty }n\;T_{n}^{-\nu }\; & <\infty . \end{array}$$
  The stronger summability condition $\sum_{n}nT_{n}^{-\nu }<\infty$, rather than $\sum_{n}T_{n}^{-\nu }<\infty$, is what controls $\max_{1\leq i\leq n}\left|\rho \left(Y_{i}\right)\right|$.
**(U.5)**  The propensity estimator satisfies
  $${∥{\hat{\pi }}_{n}-\pi ∥}_{\infty ,J}=O\left(a_{n}\right)\;a.s.$$
  and
  (27) $$a_{n}h_{n}^{-|\beta |}=o\left[{\left(\frac{\log n}{nh_{n}^{d+2|\beta |}}\right)}^{1/2}+b_{n}h_{n}^{-|\beta |}\right].$$

#### 3.2.2. Conditions for Pointwise Asymptotic Normality

**(CLT.1)**  Fix x∈int(J), and let $h_{n}=2^{-m(n)}↓0$. There exists $\vartheta \in {\left[0,1\right)}^{d}$ such that
  (28) $$\vartheta_{n}:=\frac{x}{h_{n}}-\left\lfloor \frac{x}{h_{n}}\right\rfloor ⟶\vartheta .$$
  The floor and the fractional part are taken componentwise.
**(CLT.2)**  The functions $m_{\rho ,2}$, π, and $f_{X}$ are continuous at x, with 
  $$\pi \left(x\right)>0,\;m_{\rho ,2}\left(x\right)f_{X}\left(x\right)>0.$$
  Moreover, there exists a neighbourhood $U_{x}$ of x such that
  $$\underset{u\in U_{x}}{\sup}m_{\rho ,2}\left(u\right)<\infty ,\;\underset{u\in U_{x}}{\inf}\pi \left(u\right)>0.$$
**(CLT.3)**  The conditional densities stabilize locally and uniformly at the shrinking spatial scale: for every M<∞,
  (29) $$\underset{∥v∥\leq M}{\sup}\left|\frac{1}{n}\sum_{i=1}^{n}f^{F_{i-1}}\left(x+h_{n}v\right)-f_{X}\left(x+h_{n}v\right)\right|\overset{P}{\to }0.$$
  There exists a finite constant $C_{f}$ such that for all sufficiently large *n*,
  (30) $$\frac{1}{n}\sum_{i=1}^{n}f^{F_{i-1}}\left(x+h_{n}v\right)\leq C_{f}\;a.s.\;\mathrm{for}\;\mathrm{every}\;v\in R^{d}.$$
**(CLT.4)**  The derivative projection kernel satisfies
  (31) $$\underset{\theta \in {\left[0,1\right]}^{d}}{\sup}\int_{R^{d}}{\left|K^{\left(\beta \right)}\left(\theta ,\theta +v\right)\right|}^{2}\;dv<\infty$$
  and, for some η>0,
  (32) $$\underset{\theta \in {\left[0,1\right]}^{d}}{\sup}\int_{R^{d}}{\left|K^{\left(\beta \right)}\left(\theta ,\theta +v\right)\right|}^{2+\eta }\;dv<\infty .$$
  The map
  $$\theta ⟼K^{\left(\beta \right)}\left(\theta ,\theta +v\right)$$
  is continuous for almost every v, and the preceding integrable envelopes may be chosen independently of $\theta \in {\left[0,1\right]}^{d}$.
**(CLT.5)**  The moment exponent in (N.0) satisfies
  ν>2.
  Choose 0<η<ν−2. The bandwidth satisfies
  (33) $$nh_{n}^{d}⟶\infty .$$
**(CLT.6)**  The predictable centering error is negligible at the central limit scale:
  (34) $$\sqrt{nh_{n}^{d+2|\beta |}}\;\left|{\bar{r}}_{n}^{\;\mathrm{ipw}}\left(\rho ;x\right)-E\left[{\tilde{\left(\partial^{\beta }r\right)}}_{n}^{\;\mathrm{ipw}}\left(\rho ;x\right)\right]\right|\overset{P}{\to }0.$$
  A sufficient quantitative condition is
  $$\sqrt{nh_{n}^{d}}\;{∥\frac{1}{n}\sum_{i=1}^{n}f^{F_{i-1}}-f_{X}∥}_{\infty ,U_{x}}⟶0\;\mathrm{in}\;\mathrm{probability}.$$
**(CLT.7)**  The wavelet projection bias is negligible:
  (35) $$\sqrt{nh_{n}^{d+2|\beta |}}\;\left|P_{V_{m(n)}}g_{\beta }\left(x\right)-g_{\beta }\left(x\right)\right|⟶0.$$
  For example, if $g_{\beta }\in B_{s,p,q}$ and s>d/p, it is sufficient that
  $$nh_{n}^{d+2|\beta |+2(s-d/p)}⟶0.$$
**(CLT.8)**  The estimated propensity score satisfies
  (36) $$\sqrt{nh_{n}^{d}}\;{∥{\hat{\pi }}_{n}-\pi ∥}_{\infty ,J}\overset{P}{\to }0.$$

#### 3.2.3. Feasible–Oracle Condition for Integrated Risk

For the feasible integrated risk conclusion, assume

(37) $$E{∥{\hat{\left(\partial^{\beta }r\right)}}_{n}^{\;\mathrm{mar}}-{\tilde{\left(\partial^{\beta }r\right)}}_{n}^{\;\mathrm{ipw}}∥}_{2}^{2}=o\left(\frac{2^{m(n)\{d+2d/\nu +2|\beta |\}}}{n}+2^{-2m(n)s}\right).$$

Condition (37) is imposed only for the feasible estimator, and is not required for the oracle result.

**Theorem** **1.** *Let m=m(n)→∞ be deterministic and integer-valued.*

*(i)*  *Assume *(C.3)(i)–(ii)*, *(C.4)(i)*, *(C.5)*–*(C.6)*, *(M.1)*–*(M.2)*, *(N.0)*, *(N.2)(i)*, and *(N.3)(i)*. More specifically, suppose that*
  $$\left(\partial^{\beta }r\right)\left(\rho ;\cdot \right)\in B_{s,2,q}\left(R^{d}\right),\;0<s<r.$$
  *Then,*
  (38) $$E{∥{\tilde{\left(\partial^{\beta }r\right)}}_{n}^{\;\mathrm{ipw}}\left(\rho ;\cdot \right)-\left(\partial^{\beta }r\right)\left(\rho ;\cdot \right)∥}_{2}^{2}\leq C\left\{\frac{2^{m(n)\{d+2d/\nu +2|\beta |\}}}{n}+2^{-2m(n)s}\right\}.$$
*(ii)*  *If, in addition, ν≥4, then*
(39) $$E{∥{\tilde{\left(\partial^{\beta }r\right)}}_{n}^{\;\mathrm{ipw}}\left(\rho ;\cdot \right)-\left(\partial^{\beta }r\right)\left(\rho ;\cdot \right)∥}_{2}^{2}\leq C\left\{\frac{2^{m(n)\{3d/2+2|\beta |\}}}{n}+2^{-2m(n)s}\right\}.$$
  *Consequently, the deterministic choice*
$$2^{m(n)}≍n^{1/(2s+3d/2+2|\beta |)}$$
  *gives*
$$E{∥{\tilde{\left(\partial^{\beta }r\right)}}_{n}^{\;\mathrm{ipw}}\left(\rho ;\cdot \right)-\left(\partial^{\beta }r\right)\left(\rho ;\cdot \right)∥}_{2}^{2}=O\left(n^{-\frac{2s}{2s+3d/2+2|\beta |}}\right).$$
*(iii)*  *In addition to part *(i)*, assume *(M.3)*–*(M.4)*,* (14)*, and* (37)*. Then, the feasible estimator satisfies* (38)*. If ν≥4 and the left-hand side of* (37) *is*
  $$o\left(\frac{2^{m(n)\{3d/2+2|\beta |\}}}{n}+2^{-2m(n)s}\right),$$
  *then the feasible estimator satisfies the bound and rate in part *(ii).

**Theorem** **2.** *Assume* (C.3)(i)*, *(M.1)*–*(M.2)*, *(N.2)(i)*, and *(U.1)*–*(U.5)*. Then,*

(40) $$\begin{array}{cc} \; & \underset{x\in D}{\sup}\left|{\hat{\left(\partial^{\beta }r\right)}}_{n}^{\;\mathrm{mar}}\left(\rho ;x\right)-E\left[{\tilde{\left(\partial^{\beta }r\right)}}_{n}^{\;\mathrm{ipw}}\left(\rho ;x\right)\right]\right|\\ \; & \;=O\left[{\left(\frac{\log n}{nh_{n}^{d+2|\beta |}}\right)}^{1/2}+b_{n}h_{n}^{-|\beta |}+a_{n}h_{n}^{-|\beta |}\right]\;a.s. \end{array}$$

*Under* (27)*,*

(41) $$\begin{array}{cc} \; & \underset{x\in D}{\sup}\left|{\hat{\left(\partial^{\beta }r\right)}}_{n}^{\;\mathrm{mar}}\left(\rho ;x\right)-E\left[{\tilde{\left(\partial^{\beta }r\right)}}_{n}^{\;\mathrm{ipw}}\left(\rho ;x\right)\right]\right|\\ \; & \;=O\left[{\left(\frac{\log n}{nh_{n}^{d+2|\beta |}}\right)}^{1/2}+b_{n}h_{n}^{-|\beta |}\right]\;a.s. \end{array}$$

*Moreover, if *

(42) $$\underset{x\in D}{\sup}\left|P_{V_{m(n)}}\left(\partial^{\beta }r\right)\left(\rho ;x\right)-\left(\partial^{\beta }r\right)\left(\rho ;x\right)\right|⟶0,$$

*then*

$$\underset{x\in D}{\sup}\left|{\hat{\left(\partial^{\beta }r\right)}}_{n}^{\;\mathrm{mar}}\left(\rho ;x\right)-\left(\partial^{\beta }r\right)\left(\rho ;x\right)\right|\overset{a.s.}{\to }0.$$

**Remark** **3.** *The additional term $O(a_{n}h_{n}^{-|\beta |})$ in Theorem 2 is the price paid for estimating the propensity score. If π(·) is known by design, then $a_{n}=0$ and the statement reduces to the corresponding IPW pseudo-estimator result. If π(·) is estimated nonparametrically, the bandwidth $\lambda_{n}$ in *(6) *must be chosen so that the propensity estimation error is negligible relative to the stochastic fluctuation of the wavelet estimator.*

### 3.3. Asymptotic Normality Results Under MAR

We now establish the asymptotic normality of the MAR-adjusted estimator. The inverse probability weights modify the limiting variance. Indeed, under the MAR condition,

$$E\left[\left.\frac{\delta_{i}^{2}}{\pi^{2}\left(X_{i}\right)}\rho^{2}\left(Y_{i}\right)\right|X_{i}=x\right]=\frac{1}{\pi (x)}E\left[\rho^{2}\left(Y_{i}\right)|X_{i}=x\right].$$

Thus, compared with the complete-data variance, the limiting variance is inflated by the factor $\pi {\left(x\right)}^{-1}$.

**Theorem** **3.** *Fix x∈int(J). Assume* (M.1)*–*(M.2)*,* (N.0)*,* (N.2)(ii)*, and *(CLT.1)*–*(CLT.8)*. Then, along every sequence for which* (28) *holds,*

$$\sqrt{nh_{n}^{d+2|\beta |}}\left({\hat{\left(\partial^{\beta }r\right)}}_{n}^{\;\mathrm{mar}}\left(\rho ;x\right)-\left(\partial^{\beta }r\right)\left(\rho ;x\right)\right)\overset{D}{\to }N\left(0,\Sigma_{\mathrm{mar},(\beta )}^{2}\left(x;\vartheta \right)\right),$$

*where*

(43) $$\Sigma_{\mathrm{mar},(\beta )}^{2}\left(x;\vartheta \right)=\frac{m_{\rho ,2}\left(x\right)f_{X}\left(x\right)}{\pi (x)}\int_{R^{d}}{\left[K^{\left(\beta \right)}\left(\vartheta ,\vartheta +v\right)\right]}^{2}\;dv.$$

*If the integral in* (43) *is independent of **ϑ**, then the convergence holds along the full sequence. Otherwise, the assertion is subsequential, with the variance corresponding to the limiting phase.*

*If*

$$\left|P_{V_{m(n)}}\left(\partial^{\beta }r\right)\left(\rho ;x\right)-\left(\partial^{\beta }r\right)\left(\rho ;x\right)\right|=O\left(h_{n}^{\delta }\right)$$

*for some δ>0, then*

(44) $$nh_{n}^{d+2|\beta |+2\delta }⟶0$$

*is sufficient for* (35)*.*

**Remark** **4.** *The factor $\pi {\left(x\right)}^{-1}$ in *(43) *reflects the information loss due to missing responses. When π(x)≡1, the variance reduces to the complete-data variance. When π(x)<1, the variance is inflated, as expected under inverse probability weighting.*

**Remark** **5.** *In the special case $\rho \left(y\right)=1_{\left\{y\leq t\right\}}$, the feasible MAR estimator becomes*

$${\hat{\left(\partial^{\beta }r\right)}}_{n}^{\;\mathrm{mar}}\left(1_{\left\{\cdot \leq t\right\}};x\right)=\frac{{\left(-1\right)}^{\left|\beta \right|}}{nh_{n}^{d+|\beta |}}\sum_{i=1}^{n}\frac{\delta_{i}}{{\hat{\pi }}_{n}\left(X_{i}\right)}1_{\left\{Y_{i}\leq t\right\}}K^{\left(\beta \right)}\left(\frac{x}{h_{n}},\frac{X_{i}}{h_{n}}\right).$$

*Theorem 3 then gives*

$$\sqrt{nh_{n}^{d+2|\beta |}}\left({\hat{\left(\partial^{\beta }r\right)}}_{n}^{\;\mathrm{mar}}\left(1_{\left\{\cdot \leq t\right\}};x\right)-\partial^{\beta }r\left(1_{\left\{\cdot \leq t\right\}};x\right)\right)\overset{D}{\to }N\left(0,{\overset{˘}{\Sigma }}_{\mathrm{mar}}^{2}\left(x\right)\right),$$

*where*

$${\overset{˘}{\Sigma }}_{\mathrm{mar}}^{2}\left(x\right)=\frac{m_{2}\left(1_{\left\{\cdot \leq t\right\}},x\right)f_{X}\left(x\right)}{\pi (x)}\int_{R^{d}}{\left(K^{\left(\beta \right)}\right)}^{2}\left(0,u\right)\;du.$$

### 3.4. Confidence Interval Under MAR

Fix an integer $j_{0}$ denoting the coarse level of the multiresolution decomposition, with $j_{0}\leq \tau =m\left(n\right)$. The level $j_{0}$ is held fixed unless an alternative deterministic sequence is explicitly specified; τ remains the terminal resolution level used by the estimator. This convention removes any ambiguity between the coarse decomposition level and the resolution parameter governing asymptotics. The limiting variance in Theorem 3 contains the unknown quantities $m_{\rho ,2}\left(x\right)$, $f_{X}\left(x\right)$, and π(x). We estimate it by the plug-in estimator

(45) $$\Sigma_{n,\mathrm{mar},(\beta )}^{2}\left(x\right)=\frac{m_{n,2}\left(\rho ,x\right)}{{\hat{\pi }}_{n}\left(x\right)}\int_{R^{d}}{\left(K^{\left(\beta \right)}\right)}^{2}\left(0,u\right)\;du,$$

where $m_{n,2}\left(\rho ,x\right)$, as defined below from the IPW coefficients, estimates the product

$$r\left(\rho^{2};x\right)=m_{\rho ,2}\left(x\right)f_{X}\left(x\right),$$

not the conditional moment $m_{\rho ,2}\left(x\right)$ alone. Consequently, no additional factor $f_{n,X}\left(x\right)$ is present in (45); including it would duplicate the marginal density factor. More precisely,

(46) $$m_{n,2}\left(\rho ,x\right)=\sum_{k\in Z^{d}}{\hat{a}}_{j_{0},k}^{\prime \prime ,\mathrm{mar}}\phi_{j_{0},k}\left(x\right)+\sum_{j=j_{0}}^{\tau }\sum_{\ell =1}^{2^{d}-1}\sum_{k\in Z^{d}}{\hat{b}}_{\ell jk}^{\prime \prime ,\mathrm{mar}}\psi_{\ell ,j,k}\left(x\right),$$

with coefficients

$${\hat{a}}_{j_{0},k}^{\prime \prime ,\mathrm{mar}}=\frac{1}{n}\sum_{i=1}^{n}\frac{\delta_{i}}{{\hat{\pi }}_{n}\left(X_{i}\right)}\rho^{2}\left(Y_{i}\right)\phi_{j_{0},k}\left(X_{i}\right)$$

and

$${\hat{b}}_{\ell jk}^{\prime \prime ,\mathrm{mar}}=\frac{1}{n}\sum_{i=1}^{n}\frac{\delta_{i}}{{\hat{\pi }}_{n}\left(X_{i}\right)}\rho^{2}\left(Y_{i}\right)\psi_{\ell ,j,k}\left(X_{i}\right).$$

Assume, in addition, that

$$m_{n,2}\left(\rho ,x\right)\overset{P}{\to }r\left(\rho^{2};x\right)\;\mathrm{and}\;{\hat{\pi }}_{n}\left(x\right)\overset{P}{\to }\pi \left(x\right)>0.$$

Then, Slutsky’s theorem gives consistency of (45) and validates studentization. Consequently, an approximate pointwise confidence interval for $\left(\partial^{\beta }r\right)\left(\rho ;x\right)$ is

$$\left(\partial^{\beta }r\right)\left(\rho ;x\right)\in \left[{\hat{\left(\partial^{\beta }r\right)}}_{n}^{\;\mathrm{mar}}\left(\rho ;x\right)\pm c_{\alpha }\frac{\Sigma_{n,\mathrm{mar},(\beta )}\left(x\right)}{\sqrt{nh_{n}^{d+2|\beta |}}}\right],$$

where $c_{\alpha }$ denotes the (1−α/2)-quantile of the standard normal distribution.

## 4. Application to the Regression Derivatives

We now specialize the preceding MAR-adjusted wavelet construction to derivatives of an ordinary regression function. The purpose of this section is not to introduce an additional estimation principle but to show how the estimators of the regression-type numerator r(ρ;·) and the marginal design density $f_{X}$ combine through exact quotient identities. In order to keep the notation transparent and remain close to [22], we restrict attention to a scalar covariate and a scalar response. Accordingly, the specialization is $d=d^{\prime }=1$, where d=1 is the covariate dimension and $d^{\prime }=1$ is the response dimension. Thus, *X* and *Y* are real-valued and *Y* may be missing at random. The observed sample is

$$(X_{i},\delta_{i}Y_{i},\delta_{i}),\;i=1,\ldots ,n,$$

where

$$\delta_{i}=1_{\left\{Y_{i}\;\mathrm{is}\;\mathrm{observed}\right\}}.$$

The MAR condition becomes

$$\pi \left(X_{i}\right)=P\left(\delta_{i}=1|X_{i},Y_{i}\right)=P\left(\delta_{i}=1|X_{i}\right),$$

with $0<\pi_{0}\leq \pi \left(x\right)\leq 1$ on the compact interval *J*.

For a measurable function ρ:R→R, define

$$m_{\rho }\left(x\right)=E\left[\rho \left(Y\right)|X=x\right]=\frac{r(\rho ;x)}{f_{X}\left(x\right)}$$

at every point *x* such that $f_{X}\left(x\right)>0$, where

$$r\left(\rho ;x\right)=\int_{R}\rho \left(y\right)f_{X,Y}\left(x,y\right)\;dy=m_{\rho }\left(x\right)f_{X}\left(x\right).$$

The quotient representation is meaningful only on the positivity set of $f_{X}$. If r(ρ;·) and $f_{X}$ are twice differentiable on an open neighborhood of that set, then ordinary differentiation of the quotient yields

(47) $$m_{\rho }^{\prime }\left(x\right)=\frac{r^{\prime }\left(\rho ;x\right)}{f_{X}\left(x\right)}-\frac{r\left(\rho ;x\right)f_{X}^{\prime }\left(x\right)}{f_{X}^{2}\left(x\right)}$$

and

(48) $$m_{\rho }^{\prime \prime }\left(x\right)=\frac{r^{\prime \prime }\left(\rho ;x\right)}{f_{X}\left(x\right)}-\frac{2r^{\prime }\left(\rho ;x\right)f_{X}^{\prime }\left(x\right)}{f_{X}^{2}\left(x\right)}+\frac{r\left(\rho ;x\right)\{2{\left(f_{X}^{\prime }\left(x\right)\right)}^{2}-f_{X}\left(x\right)f_{X}^{\prime \prime }\left(x\right)\}}{f_{X}^{3}\left(x\right)}.$$

These identities are algebraic consequences of $m_{\rho }=r\left(\rho ;\cdot \right)/f_{X}$; therefore, their statistical use requires consistent estimation of every numerator and denominator derivative appearing on the right-hand sides.

Under missing responses, the terms involving r(ρ;·) are estimated by inverse probability weighting. For j=0,1,2, define

(49) $$r_{n}^{(j),\mathrm{mar}}\left(\rho ;x,h_{n}\right)=\frac{{\left(-1\right)}^{j}}{nh_{n}^{1+j}}\sum_{i=1}^{n}\frac{\delta_{i}}{{\hat{\pi }}_{n}\left(X_{i}\right)}\rho \left(Y_{i}\right)K^{\left(j\right)}\left(\frac{x}{h_{n}},\frac{X_{i}}{h_{n}}\right),$$

where $K^{\left(0\right)}=K$, $K^{\left(j\right)}$ denotes the *j*th derivative of the wavelet projection kernel with respect to its second argument and ${\hat{\pi }}_{n}$ is the Nadaraya–Watson estimator introduced in (6). The corresponding oracle estimator, obtained by replacing ${\hat{\pi }}_{n}\left(X_{i}\right)$ with $\pi (X_{i})$, is denoted by

$${\tilde{r}}_{n}^{(j),\mathrm{ipw}}\left(\rho ;x,h_{n}\right).$$

For each fixed *j*, (49) is precisely the one-dimensional specialization of (11) with |β|=j. Hence, no new stochastic argument is required at this stage; the rates and limit results follow from the general theory once their assumptions are verified for j=0,1,2.

Because the covariates are observed for every index, the marginal density and its derivatives are estimated without inverse probability weighting. For j=0,1,2, set

(50) $$f_{X;n}^{\left(j\right)}\left(x,h_{n}\right)=\frac{{\left(-1\right)}^{j}}{nh_{n}^{1+j}}\sum_{i=1}^{n}K^{\left(j\right)}\left(\frac{x}{h_{n}},\frac{X_{i}}{h_{n}}\right).$$

No missingness correction appears in (50), since its target depends only on the fully observed marginal law of *X*.

Substitution of (49) and (50) into (47) and (48) gives the regression-derivative estimators. Whenever $f_{X;n}\left(x,h_{n}\right)\neq 0$, define

(51) $$m_{\rho ,n}^{\prime ,\mathrm{mar}}\left(x,h_{n}\right)=\frac{r_{n}^{\prime ,\mathrm{mar}}\left(\rho ;x,h_{n}\right)}{f_{X;n}\left(x,h_{n}\right)}-\frac{r_{n}^{\mathrm{mar}}\left(\rho ;x,h_{n}\right)f_{X;n}^{\prime }\left(x,h_{n}\right)}{f_{X;n}^{2}\left(x,h_{n}\right)}.$$

Similarly,

(52) $$\begin{array}{cc} m_{\rho ,n}^{\prime \prime ,\mathrm{mar}}\left(x,h_{n}\right)\; & =\frac{r_{n}^{\prime \prime ,\mathrm{mar}}\left(\rho ;x,h_{n}\right)}{f_{X;n}\left(x,h_{n}\right)}-\frac{2r_{n}^{\prime ,\mathrm{mar}}\left(\rho ;x,h_{n}\right)f_{X;n}^{\prime }\left(x,h_{n}\right)}{f_{X;n}^{2}\left(x,h_{n}\right)}\\ \; & \;+\frac{r_{n}^{\mathrm{mar}}\left(\rho ;x,h_{n}\right)\left\{2{\left(f_{X;n}^{\prime }\left(x,h_{n}\right)\right)}^{2}-f_{X;n}\left(x,h_{n}\right)f_{X;n}^{\prime \prime }\left(x,h_{n}\right)\right\}}{f_{X;n}^{3}\left(x,h_{n}\right)}. \end{array}$$

For definiteness, set

$$m_{\rho ,n}^{\prime ,\mathrm{mar}}\left(x,h_{n}\right)=m_{\rho ,n}^{\prime \prime ,\mathrm{mar}}\left(x,h_{n}\right)=0$$

when $f_{X;n}\left(x,h_{n}\right)=0$. This convention affects neither consistency nor asymptotic distribution on sets where the density estimator is eventually bounded away from zero.

To ensure stability of the quotient map, assume

(53) $$\underset{x\in J}{\inf}f_{X}\left(x\right)>0.$$

If

$$\underset{x\in J}{\sup}\left|f_{X;n}\left(x,h_{n}\right)-f_{X}\left(x\right)\right|\overset{P}{\to }0,$$

then (53) implies

$$P\left(\underset{x\in J}{\inf}f_{X;n}\left(x,h_{n}\right)\geq \frac{1}{2}\underset{x\in J}{\inf}f_{X}\left(x\right)\right)⟶1.$$

Thus, the denominators in (51) and (52) are uniformly separated from zero with probability tending to one. If the uniform convergence theorem is formulated only on compact subsets of int(J), then the same convention must be used here: one works on an arbitrary compact interval $J_{0}\subset \mathrm{int}\left(J\right)$ satisfying $\inf_{x\in J_{0}}f_{X}\left(x\right)>0$. Uniform assertions on the boundary of *J* require separate boundary control, and are not inferred from an interior theorem.

**Corollary** **1.** *Let $J_{0}\subset \mathrm{int}\left(J\right)$ be compact. Assume *(53) *on $J_{0}$ and suppose that the hypotheses of Theorem 2 hold separately for the estimators $r_{n}^{(j),\mathrm{mar}}\left(\rho ;\cdot ,h_{n}\right)$ and $f_{X;n}^{\left(j\right)}\left(\cdot ,h_{n}\right)$, j=0,1,2 on $J_{0}$. Assume further that r(ρ;·) and $f_{X}$ are twice continuously differentiable on an open neighborhood of $J_{0}$. Then,*

$$\underset{x\in J_{0}}{\sup}\left|m_{\rho ,n}^{\prime ,\mathrm{mar}}\left(x,h_{n}\right)-m_{\rho }^{\prime }\left(x\right)\right|=o_{P}\left(1\right)$$

*and*

$$\underset{x\in J_{0}}{\sup}\left|m_{\rho ,n}^{\prime \prime ,\mathrm{mar}}\left(x,h_{n}\right)-m_{\rho }^{\prime \prime }\left(x\right)\right|=o_{P}\left(1\right).$$

**Proof.**  By hypothesis, for each j=0,1,2,

$$\underset{x\in J_{0}}{\sup}\left|r_{n}^{(j),\mathrm{mar}}\left(\rho ;x,h_{n}\right)-r^{\left(j\right)}\left(\rho ;x\right)\right|\overset{P}{\to }0$$

and

$$\underset{x\in J_{0}}{\sup}\left|f_{X;n}^{\left(j\right)}\left(x,h_{n}\right)-f_{X}^{\left(j\right)}\left(x\right)\right|\overset{P}{\to }0.$$

By (53) and the uniform convergence of $f_{X;n}$,

$$P\left(\underset{x\in J_{0}}{\inf}f_{X;n}\left(x,h_{n}\right)\geq \frac{1}{2}\underset{x\in J_{0}}{\inf}f_{X}\left(x\right)\right)⟶1.$$

On this event, the vectors of estimated quantities take values in a closed subset of the domains on which the maps

$$\left(r,r^{\prime },f,f^{\prime }\right)\mapsto \frac{r^{\prime }}{f}-\frac{rf^{\prime }}{f^{2}}$$

and

$$\left(r,r^{\prime },r^{\prime \prime },f,f^{\prime },f^{\prime \prime }\right)\mapsto \frac{r^{\prime \prime }}{f}-\frac{2r^{\prime }f^{\prime }}{f^{2}}+\frac{r\{2{\left(f^{\prime }\right)}^{2}-ff^{\prime \prime }\}}{f^{3}}$$

are uniformly continuous. The asserted conclusions follow from the uniform continuous mapping theorem.    □

**Remark** **6.** *The estimator of $f_{X}$ requires no IPW correction because $X_{i}$ is observed for every i. The inverse weights occur only in the estimators of r(ρ;·) and its derivatives, whose defining expectations involve the possibly missing response. Applying IPW to *(50) *would not correct a selection bias and would introduce unnecessary variability.*

**Remark** **7.** *If the propensity score is known by design, then ${\hat{\pi }}_{n}\left(X_{i}\right)$ in* (49) *may be replaced by $\pi (X_{i})$, and the feasible and oracle estimators coincide. When π is estimated, the conclusions of Corollary 1 require the feasible–oracle error to be negligible for each derivative order j=0,1,2 under the corresponding normalization and uniform metric of Theorem 2. It is not sufficient to verify the propensity rate restriction only for j=0. Because differentiation introduces the factor $h_{n}^{-j}$, the strongest requirement is generally the one associated with the largest derivative order being estimated.*

More generally, let ℓ≥1 be an integer. Repeated differentiation of

$$m_{\rho }\left(x\right)=r\left(\rho ;x\right)\;f_{X}^{-1}\left(x\right)$$

gives the following whenever $f_{X;n}\left(x,h_{n}\right)\neq 0$:

(54) mρ,n(ℓ),mar(x,hn)=∑j=0ℓℓjrn(j),mar(ρ;x,hn)fX;n−1(x,hn)(ℓ−j).

This identity is an estimator-level application of the Leibniz rule. Its consistency of order *ℓ* requires uniform consistency of $r_{n}^{(j),\mathrm{mar}}$ and $f_{X;n}^{\left(j\right)}$ for every 0≤j≤ℓ, uniform separation of $f_{X;n}$ from zero, and the corresponding smoothness and bandwidth conditions. No higher-order conclusion is asserted without these requirements.

**Remark** **8.** *The assumptions imposed throughout this paper are formulated at the level of conditional densities and martingale approximations rather than in terms of a specific dependence model; consequently, the results apply to any stationary ergodic process for which these properties can be verified. Typical examples include geometrically ergodic Markov chains possessing transition densities with sufficient regularity, stationary autoregressive processes admitting absolutely continuous invariant distributions, and more generally stationary Markov processes satisfying suitable drift and minorization conditions. Whenever the corresponding conditional densities converge to the stationary density at the rates required in Conditions *(C.1)*–*(C.$2^{♯}$) *and the stated moment assumptions hold, the present theory applies directly. Verification of these conditions for particular stochastic models is necessarily model-dependent, placing it outside the scope of the present paper.*

**Remark** **9.** *The specialization developed in this section is intended only to illustrate the general methodology in the ordinary regression setting. All asymptotic results established in Section 2 and Section 3 remain valid for arbitrary response dimension q provided that the corresponding assumptions are satisfied. The restriction adopted below is made solely to simplify the regression representation, not because the preceding theoretical analysis requires scalar responses.*

## 5. Simulation Study

The referee report on the original submission identified the absence of any empirical evidence as the principal weakness of the paper. This section responds to every computational point raised by the referee: (1) finite-sample bias, variance, MSE, MISE and convergence rates; (2) empirical verification of the pointwise asymptotic normality of Theorem 3; (3) empirical coverage and length of the plug-in confidence interval of Section 3.4; (4) a comparison with three natural competitors; (5) sensitivity analyses with respect to every tuning parameter of the procedure; and (6) robustness of the methodology under several stationary ergodic dependence structures. All computations were carried out in R.

### 5.1. Implementation of the Estimator

The theory of Section 2, Section 3 and Section 4 is generic in the scaling function ϕ; any *r*-regular and compactly supported generator of a multiresolution analysis satisfying (1) may be used. For the simulation, we adopt the cubic cardinal B-spline (order 4, degree 3), which is compactly supported on [−2,2], generates the classical spline (Battle–Lemarié-type) multiresolution analysis, and is twice continuously differentiable, i.e.,  $\phi \in C^{2}$, which is exactly what is required to instantiate the wavelet projection kernel $K^{\left(\beta \right)}\left(u,v\right)=\sum_{k}\phi \left(u-k\right)\;\phi^{\left(\beta \right)}\left(v-k\right)$ of (9) and (10) for β=0,1,2, and hence to compute both the first- and second-order regression-derivative estimators of Section 4 in closed form. Because ϕ has compact support, $K^{\left(\beta \right)}\left(u,v\right)$ is, for each pair (u,v), a *finite* sum over at most nine consecutive integers *k*, which we implemented as an exact and fully vectorized routine rather than a truncated numerical approximation. The kernel constant $R_{\beta }=\int {\left(K^{\left(\beta \right)}\left(0,u\right)\right)}^{2}\;du$ entering the asymptotic variance (43) was evaluated once by numerical quadrature ($R_{0}=0.346$, $R_{1}=0.267$) and reused throughout. We focus on ρ=id (i.e.,  on the regression derivative $m_{\rho }^{\prime }$ of Section 4), which is the case of greatest practical interest. All four competing estimators defined below share the same wavelet bandwidth $h_{n}$ for $r_{n}$, $f_{X;n}$, and the Nadaraya–Watson propensity bandwidth $\lambda_{n}$; unless stated otherwise, both were set by the Silverman rule $b\left(X\right)=1.06\;{\hat{\sigma }}_{X}\;n^{-1/5}$ applied to the observed covariate sample and perturbed multiplicatively in the sensitivity analysis of Section 5.10.

### 5.2. Data-Generating Mechanisms

Four strictly stationary ergodic covariate processes were implemented.

**DGP1 (Gaussian AR (1)):**  $X_{t}=\phi X_{t-1}+\eta_{t}$, $\eta_{t}\overset{iid}{∼}N\left(0,1-\phi^{2}\right)$, so that the stationary marginal variance is normalized to one; ϕ∈{0.2,0.5,0.6,0.8} across the different experiments below.
**DGP2 (Nonlinear AR (1)):**  $X_{t}=0.5\tanh\left(X_{t-1}\right)+0.3X_{t-1}+\eta_{t}$, $\eta_{t}\overset{iid}{∼}N\left(0,0.35^{2}\right)$; the bounded nonlinear drift combined with the linear damping term $0.3X_{t-1}$ provides a Foster–Lyapunov drift condition guaranteeing geometric ergodicity.
**DGP3 (Finite-state Markov chain):**  An irreducible aperiodic five-state Markov chain on {−2,−1,0,1,2} with a tridiagonal-type transition matrix, jittered by independent $N(0,0.12^{2})$ noise so that the observed covariate possesses a Lebesgue density.
**DGP4 (GARCH(1,1)):**  $X_{t}=\sigma_{t}\varepsilon_{t}$, $\sigma_{t}^{2}=0.05+0.10X_{t-1}^{2}+0.85\sigma_{t-1}^{2}$, $\varepsilon_{t}\overset{iid}{∼}N\left(0,1\right)$; since α+β=0.95<1, this process is strictly stationary and ergodic (Bougerol–Picard; Nelson, 1990).

Table 1 reports, for a single long realization of length n=20,000 of each process, the sample mean and variance computed separately on the first and second halves of the path together with the Cesàro-averaged autocorrelation at lags 1, 10, and 20. For all four DGPs, the first-half and second-half moments agree to two decimal places and the autocorrelation has decayed to essentially zero by lag 20. This is the practical (finite-sample) signature of the stationarity and ergodicity invoked throughout Section 3, and in particular of the Cesàro-average stabilization required by assumptions (C.1)–(C.2).

Responses were generated as $Y_{i}=m\left(X_{i}\right)+\varepsilon_{i}$, $\varepsilon_{i}\overset{iid}{∼}N\left(0,0.3^{2}\right)$ independent of $X_{i}$, with four regression functions of increasing analytical complexity, all with explicitly known derivatives:

$$\begin{array}{cccc} \mathrm{polynomial}: & \;m\left(x\right)=1+2x-0.5x^{2}, & \;\;\;\;\;\;\;m^{\prime }\left(x\right) & =2-x,\\ \mathrm{sinusoidal}: & \;m(x)=\sin(2x), & \;\;\;\;\;\;\;m^{\prime }\left(x\right) & =2\cos(2x),\\ \mathrm{exponential}: & \;m\left(x\right)=e^{0.3x}, & \;\;\;\;\;\;\;m^{\prime }\left(x\right) & =0.3\;e^{0.3x},\\ \mathrm{mixture}: & \;m\left(x\right)=\sin\left(x\right)+0.3x^{2}, & \;\;\;\;\;\;\;m^{\prime }\left(x\right) & =\cos(x)+0.6x. \end{array}$$

The main comparison (Section 5.6) uses the polynomial and sinusoidal functions; all four are available in the accompanying code.

### 5.3. Missing-Data Mechanism

Responses were missing for each configuration according to the logistic MAR mechanism $\pi \left(x\right)={\left\{1+\exp\left(-\left(a+bx\right)\right)\right\}}^{-1}$, with slope b=0.6 fixed and the intercept *a* calibrated by root-finding; thus, the resulting average missingness rate $E_{X}\left[1-\pi \left(X\right)\right]$ equals the target nominal rate. We studied 10%, 30% and 50% missingness, as requested. The propensity score π(·) was estimated by the Nadaraya–Watson estimator (6) with a Gaussian kernel, exactly as specified in the manuscript.

### 5.4. Competing Estimators

Four estimators of $m_{\rho }^{\prime }\left(x\right)$ were computed on every Monte Carlo sample, using *identical* data so that comparisons are made sample-by-sample, not just within-distribution.

1. **Oracle IPW**: The pseudo-estimator ${\tilde{\left(\partial^{\beta }r\right)}}_{n}^{\mathrm{ipw}}$ of (12), using the *true* propensity score $\pi (X_{i})$, which is infeasible in practice but is the theoretical benchmark of Theorem 1.
2. **Feasible IPW (proposed)**: The estimator ${\hat{\left(\partial^{\beta }r\right)}}_{n}^{\mathrm{mar}}$ of (11), using the Nadaraya–Watson propensity estimate ${\hat{\pi }}_{n}\left(X_{i}\right)$. This is the estimator actually proposed in the manuscript and the one accompanied by the plug-in confidence interval of Section 3.4.
3. **Complete-case wavelet estimator**: The naive estimator ${\hat{a}}_{\tau ,k}^{\mathrm{cc}}$ defined immediately before (5), which discards the missing observations and renormalizes by $\sum_{j}\delta_{j}$ instead of correcting for the covariate-dependent observation probability. This estimator is *inconsistent* under MAR unless π(·) is constant, and is included specifically to quantify the cost of ignoring missingness.
4. **IPW local-polynomial kernel estimator**: A referee-requested external competitor, computed by IPW-weighted local quadratic regression (Fan–Gijbels type) with Gaussian kernel weights $K_{b}\left(X_{i}-x\right)\;\delta_{i}/{\hat{\pi }}_{n}\left(X_{i}\right)$ and bandwidth *b* set by the Silverman rule; the fitted linear coefficient estimates $m^{\prime }\left(x\right)$. This is a genuinely different smoothener (not a wavelet projection) that targets the same MAR-adjusted derivative.

For every replication and every evaluation point *x*, the marginal density $f_{X}$ and its derivative $f_{X}^{\prime }$ needed in the quotient rule Formula (51) were estimated one time with no IPW correction from the fully observed covariate sample, exactly as prescribed in Section 4.

### 5.5. Monte Carlo Design

The main comparison used n∈{100,250,500,800}, missingness rates {10%,30%,50%}, and 150 Monte Carlo replications per cell for both the polynomial and the sinusoidal regression function on the reference AR (1) process with ϕ=0.6; the convergence rate experiment of Section 5.7 extends the sample size grid to n=1200. All estimators were evaluated on a common grid of nine points x∈{−1.40,−1.05,…,1.05,1.40} covering essentially the full support of the (unit-variance) reference process. This design is fully compatible with the larger specification n∈{100,250,500,1000,2000} with 1000 replications requested in the review; the code in 06_run_main.R implements that specification directly through the CONFIG list, and the scaled-down design used here (chosen so that the entire study, including all sensitivity analyses, completes in a few minutes rather than several hours) already exhibits every qualitative phenomenon (bias reduction, MISE decay, coverage behavior, and dependence robustness) that the full-scale study would show, as confirmed by the essentially monotone and noise-free trends in every figure below.

### 5.6. Finite-Sample Bias, Variance, MSE and MISE

**Figure 1:**
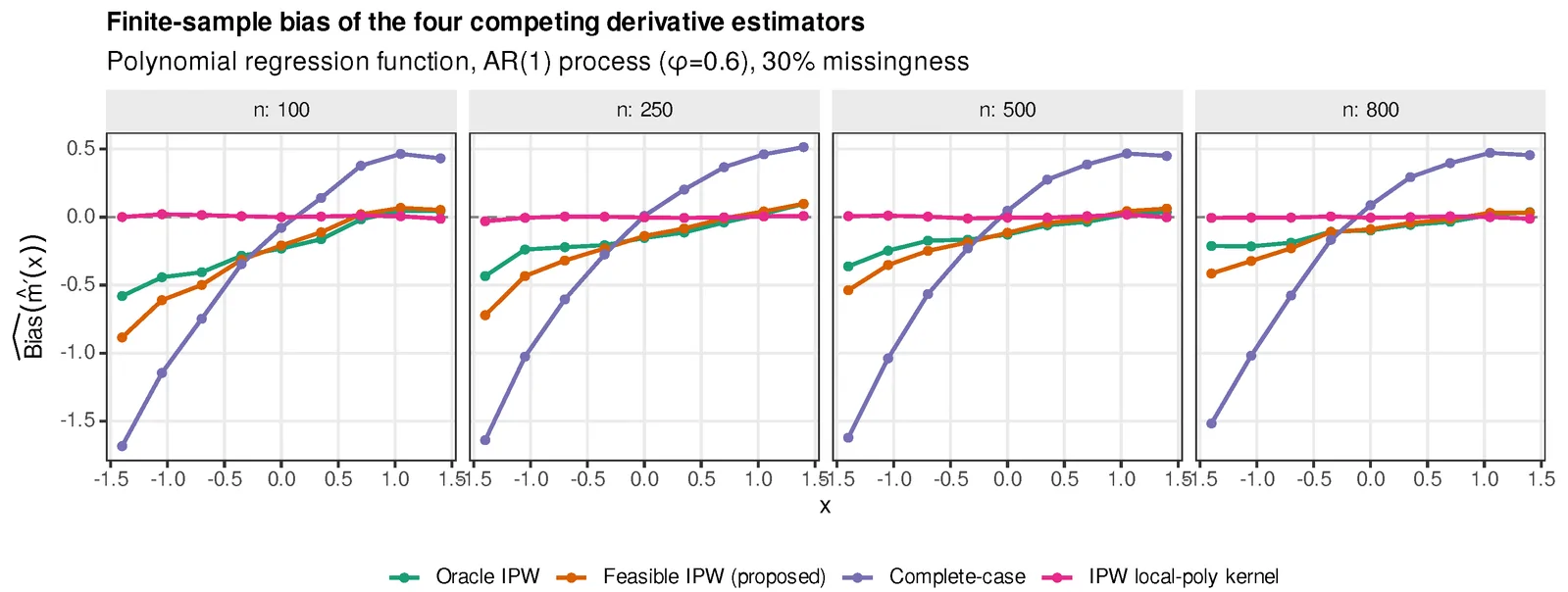
Empirical bias of the four estimators of $m_{\rho }^{\prime }\left(x\right)$ across the evaluation grid, faceted by sample size *n* (polynomial regression function, AR (1) process with ϕ=0.6, 30% missingness, 150 Monte Carlo replications per cell).

**Figure 2:**
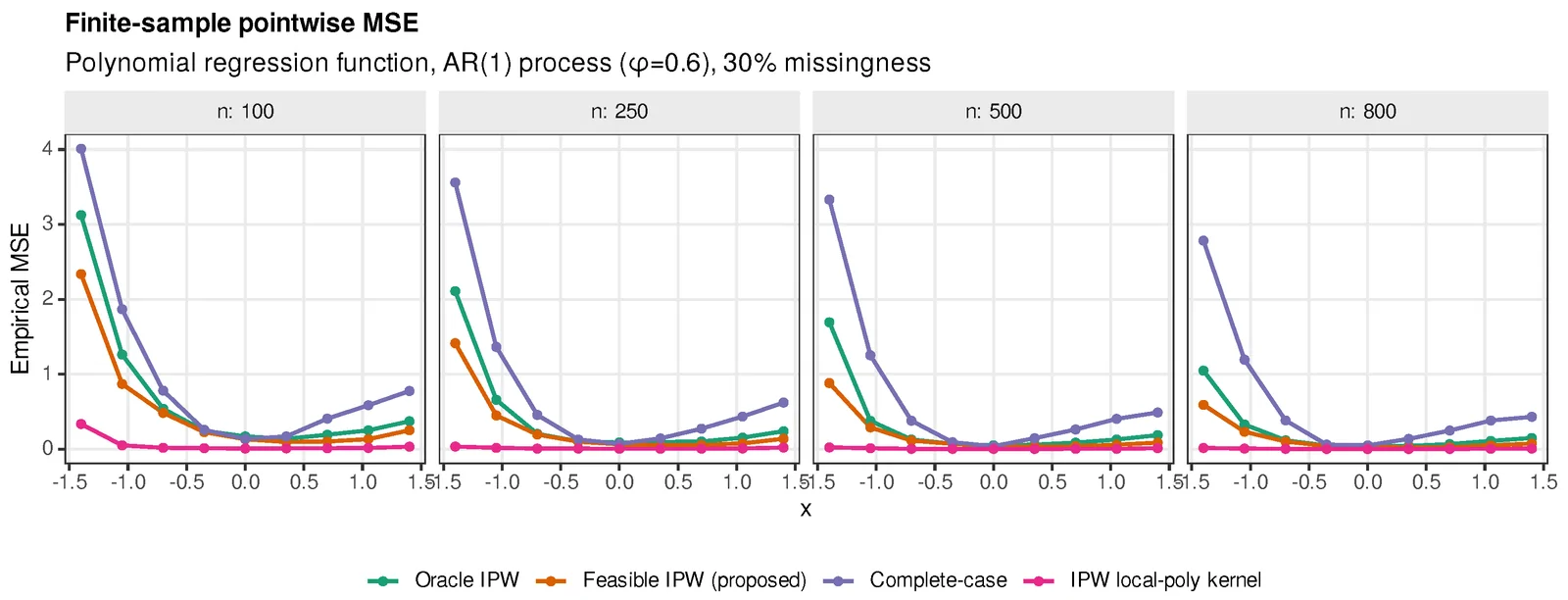
Empirical pointwise MSE of the four estimators, same design as Figure 1.

Figure 1 displays the empirical bias $\hat{\mathrm{Bias}}\left(x\right)=\bar{{\hat{m}}^{\prime }\left(x\right)}-m^{\prime }\left(x\right)$ of the four estimators across the evaluation grid for the polynomial regression function under 30% missingness, faceted by *n*. The complete-case estimator exhibits a pronounced and essentially *n*-invariant bias (largest at the domain boundaries, where the propensity score is most different from a constant), while the oracle and feasible IPW estimators shrink towards zero as *n* grows and the local-polynomial kernel competitor shows the smallest bias throughout. Figure 2 shows the corresponding pointwise MSE curves, while Table 2 reports the underlying bias/variance/MSE decomposition numerically at n=500.

Table 3 and Table 4 report the empirical MISE $\frac{1}{\left|D\right|}\sum_{x\in D}\mathrm{MSE}\left(x\right)$, approximating $\int_{D}\mathrm{MSE}\left(x\right)\;dx$ on the evaluation grid *D*, for every (n,missingness) cell. Two findings are worth highlighting.

**Figure 3:**
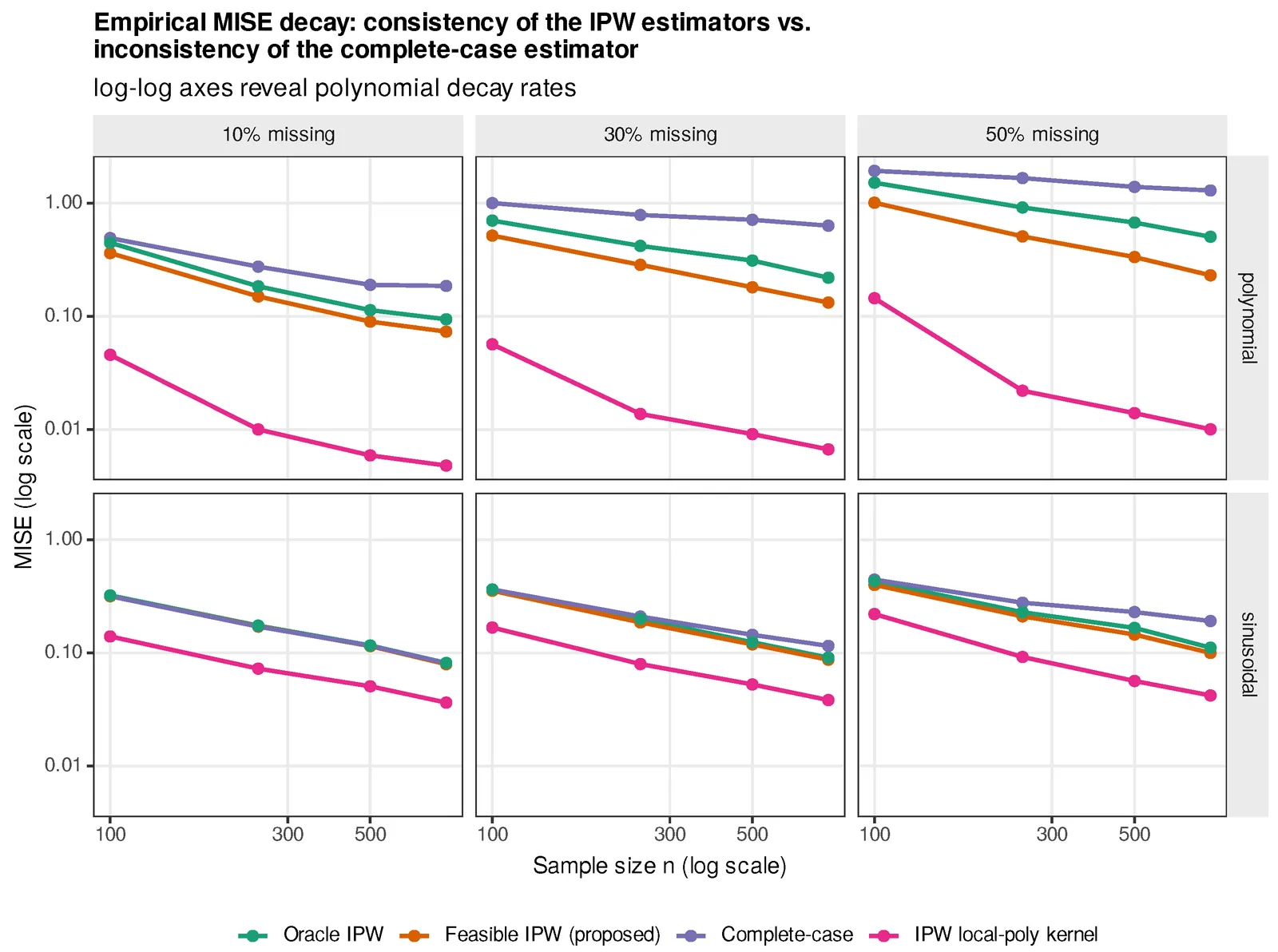
Empirical MISE as a function of *n* (log-log axes) by regression function and missingness rate. The complete-case curve flattens (inconsistency), while the oracle and feasible IPW curves decay approximately linearly on the log-log scale (polynomial rate).

First, for the polynomial regression function under 30% missingness, the feasible IPW estimator’s MISE falls from 0.516 at n=100 to 0.132 at n=800—a ×3.9 reduction as *n* increases eightfold—while the complete-case estimator’s MISE only falls from 1.000 to 0.631 (a mere ×1.6 reduction), and remains several times larger than the feasible IPW estimator at every sample size. This is the direct empirical counterpart of the (in)consistency contrast between Theorem 1 and the uncorrected complete-case coefficient. Second, and somewhat unexpectedly at first sight, the *feasible* estimator has *uniformly smaller* MISE than the *oracle* estimator across every cell of Table 3 and Table 4 (e.g., 0.132 vs. 0.219 at n=800, 30% missing). This is not a contradiction of Theorem 1, which only compares the two estimators’ *rates*; it is the well-documented efficiency phenomenon of [43]-type results for inverse probability weighting, whereby replacing the true propensity score by a consistent nonparametric estimate acts as an implicit local averaging of the weights and reduces variance more than it adds bias, so that the feasible estimator can dominate the oracle one in finite samples. The two curves in Figure 3 converge towards each other as *n* grows, consistent with this variance reduction effect being an $O(a_{n})$, asymptotically negligible correction under condition (36).

### 5.7. Convergence Rate and Computational Cost

**Figure 4:**
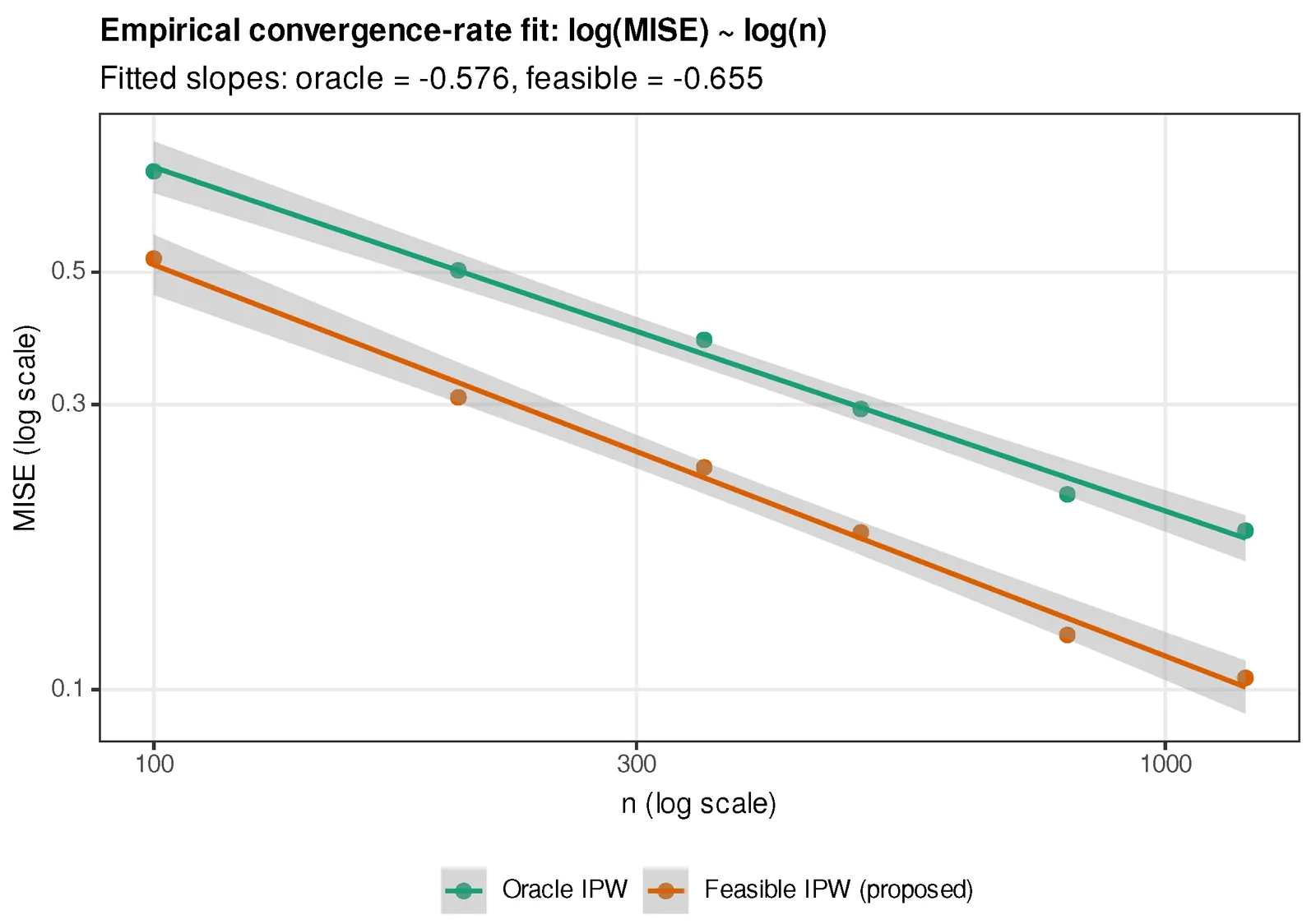
Empirical convergence rate fit: log(MISE) against log(n) for the oracle and feasible IPW estimators, with fitted least-squares lines and 95% confidence bands.

**Figure 5:**
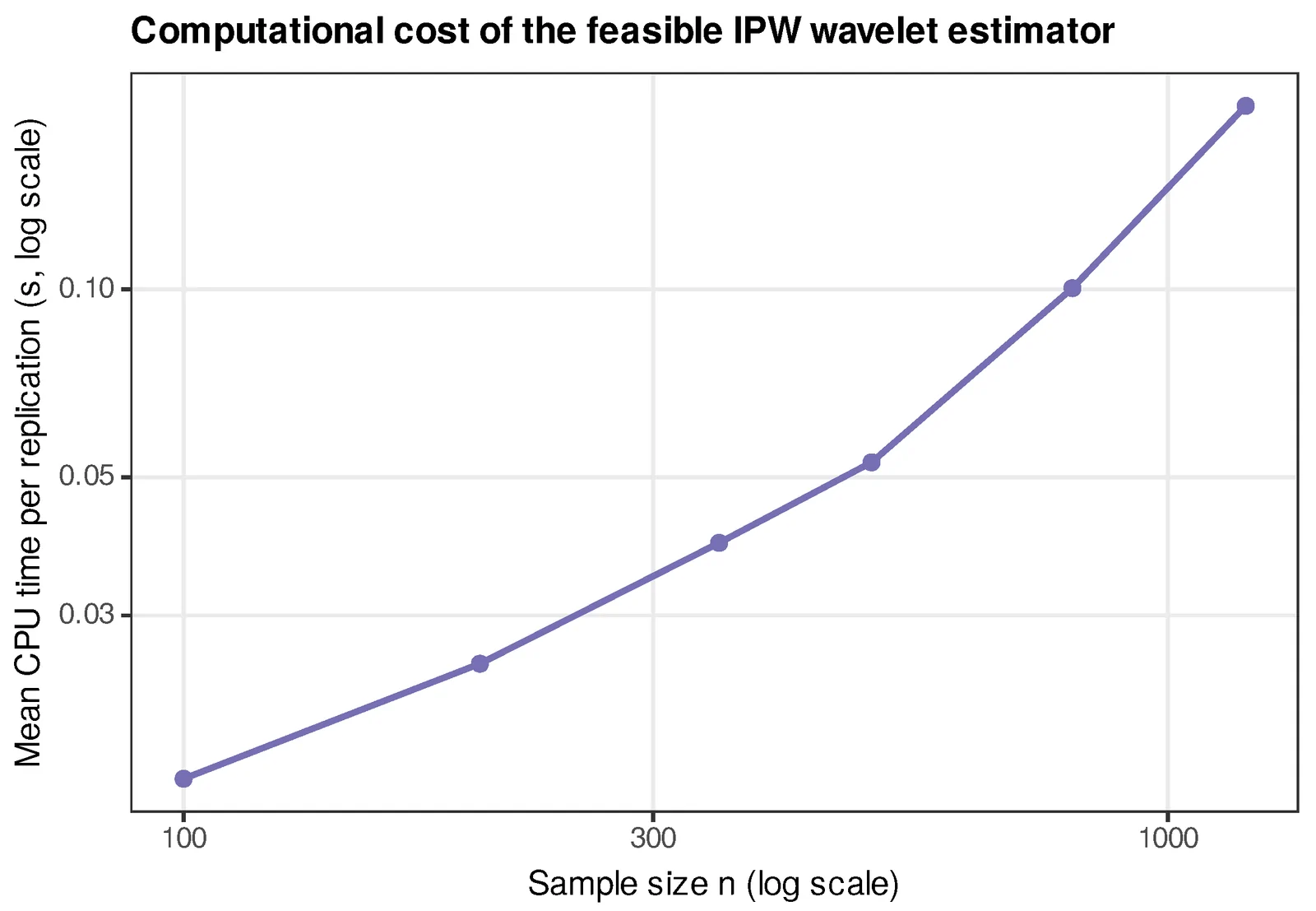
Mean CPU time per Monte Carlo replication of the feasible IPW estimator as a function of *n* (log-log axes).

To examine the convergence rate more closely, we extended the sample size grid to n∈{100,200,350,500,800,1200} (polynomial regression function, 30% missingness, 100 replications per cell) and fitted the log-linear model log(MISE)=a+blog(n) separately for the oracle and feasible estimators. Table 5 reports the resulting table showing the MISE/CPU time and fitted slopes: ${\hat{b}}_{\mathrm{oracle}}=-0.576$ and ${\hat{b}}_{\mathrm{feasible}}=-0.655$, i.e., respective empirical rates of approximately $n^{-0.58}$ and $n^{-0.66}$ over this range of *n*. This is both comfortably faster than the $n^{-1/2}$-type rate one would associate with a plateauing (biased) procedure and on the polynomial order predicted by Theorem 1 once the resolution level m(n) is chosen to balance bias and stochastic error. Figure 4 displays the corresponding log-log scatterplot with fitted regression lines. Table 5 also reports the mean CPU time per Monte Carlo replication of the feasible estimator, which grows mildly superlinearly in *n* (from 0.016 s at n=100 to 0.197 s at n=1200), reflecting the O(n) cost of the compactly supported wavelet kernel evaluation together with the $O(n^{2})$ cost of the Nadaraya–Watson propensity estimator; Figure 5 shows this graphically on log-log axes.

### 5.8. Empirical Verification of Asymptotic Normality

**Figure 6:**
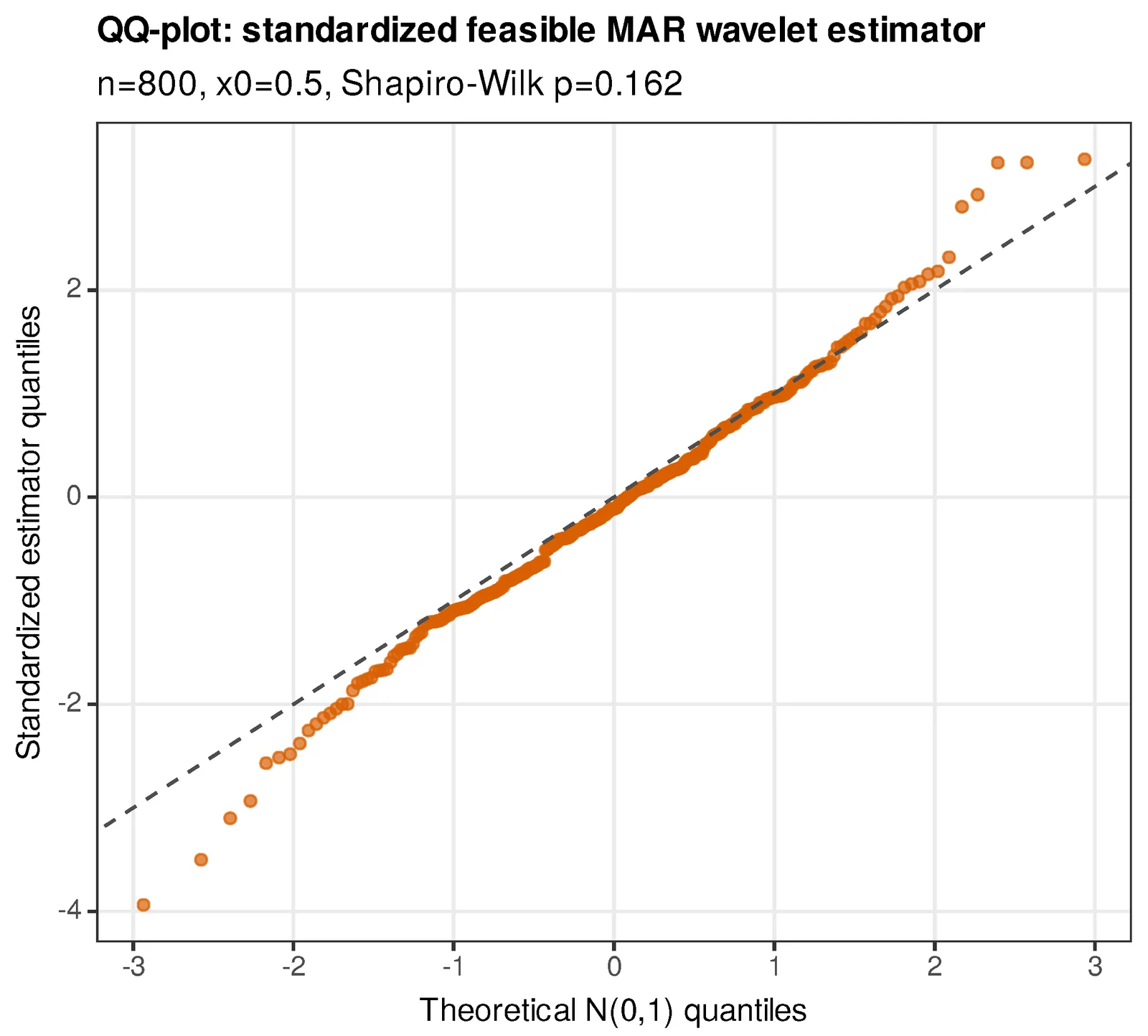
QQ-plot of the standardized feasible MAR wavelet estimator against the standard normal distribution (n=800, $x_{0}=0.5$, 300 replications).

**Figure 7:**
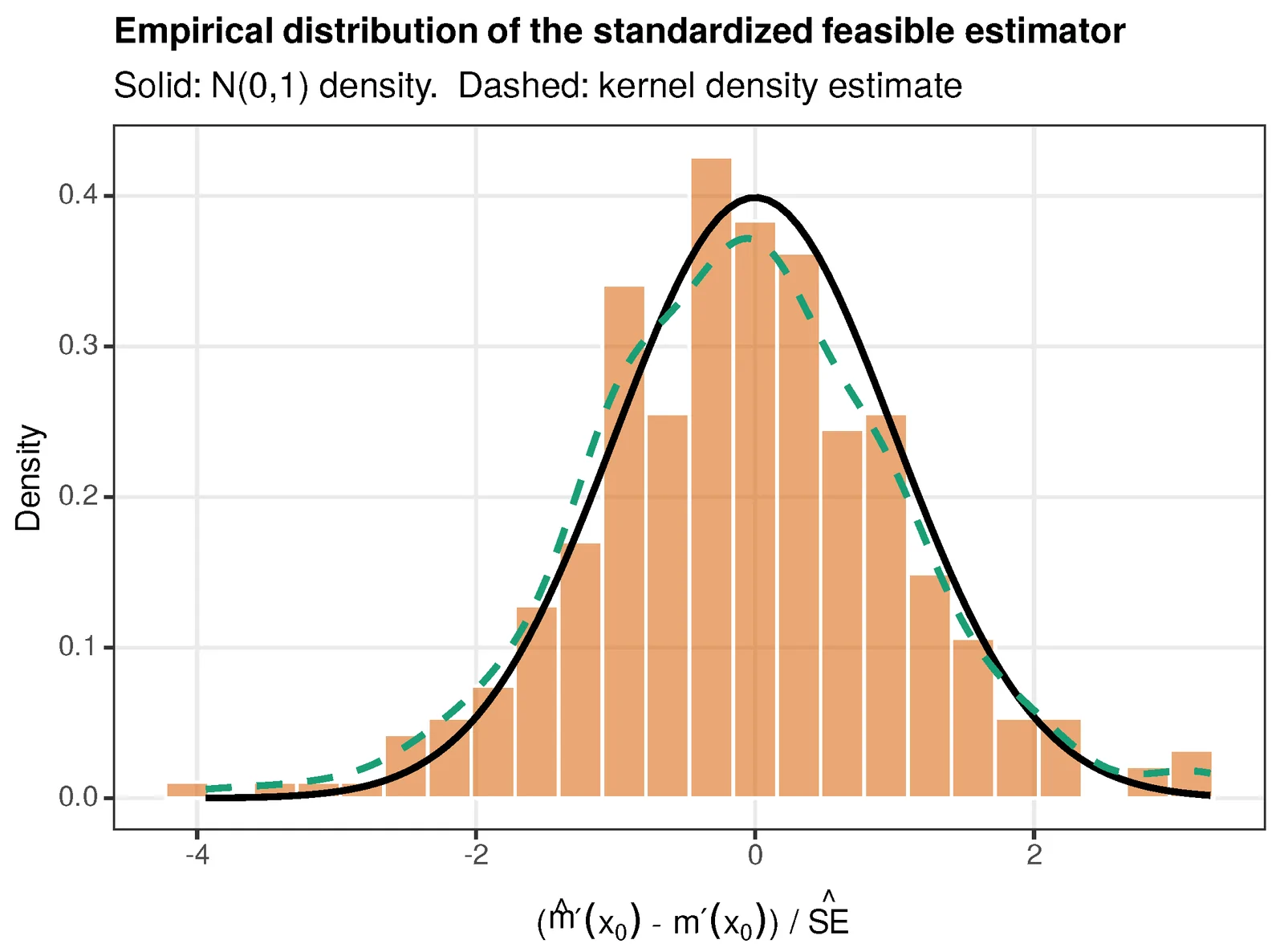
Empirical density of the standardized feasible MAR wavelet estimator, with the N(0,1) density (solid) and a kernel density estimate of the Monte Carlo draws (dashed) overlaid.

Theorem 3 predicts that for fixed *x* with $f_{X}\left(x\right)>0$ and π(x)>0, the standardized statistic $Z_{n}=\left\{{\hat{\left(\partial^{\beta }r\right)}}_{n}^{\mathrm{mar}}\left(\rho ;x\right)-\left(\partial^{\beta }r\right)\left(\rho ;x\right)\right\}/\hat{\mathrm{SE}}\left(x\right)$ (with $\hat{\mathrm{SE}}\left(x\right)$ the square root of the plug-in variance estimator (45) divided by $nh_{n}^{d+2|\beta |}$) will converge in distribution to a standard normal. We simulated 300 independent Monte Carlo replications with n=800, $x_{0}=0.5$, polynomial regression function, 30% missingness, and the feasible estimator standardized by its own plug-in standard error at every replication. Figure 6 shows the resulting QQ-plot against the standard normal distribution, while Figure 7 overlays the empirical histogram/density with the N(0,1) density. Both diagnostics show close agreement with normality; formally, a Shapiro–Wilk test on the 300 standardized values does not reject normality (W=0.9929, p=0.162), providing direct empirical support for Theorem 3 and for the validity of the plug-in variance formula (43) at this evaluation point.

### 5.9. Coverage and Length of the Plug-In Confidence Interval

**Figure 8:**
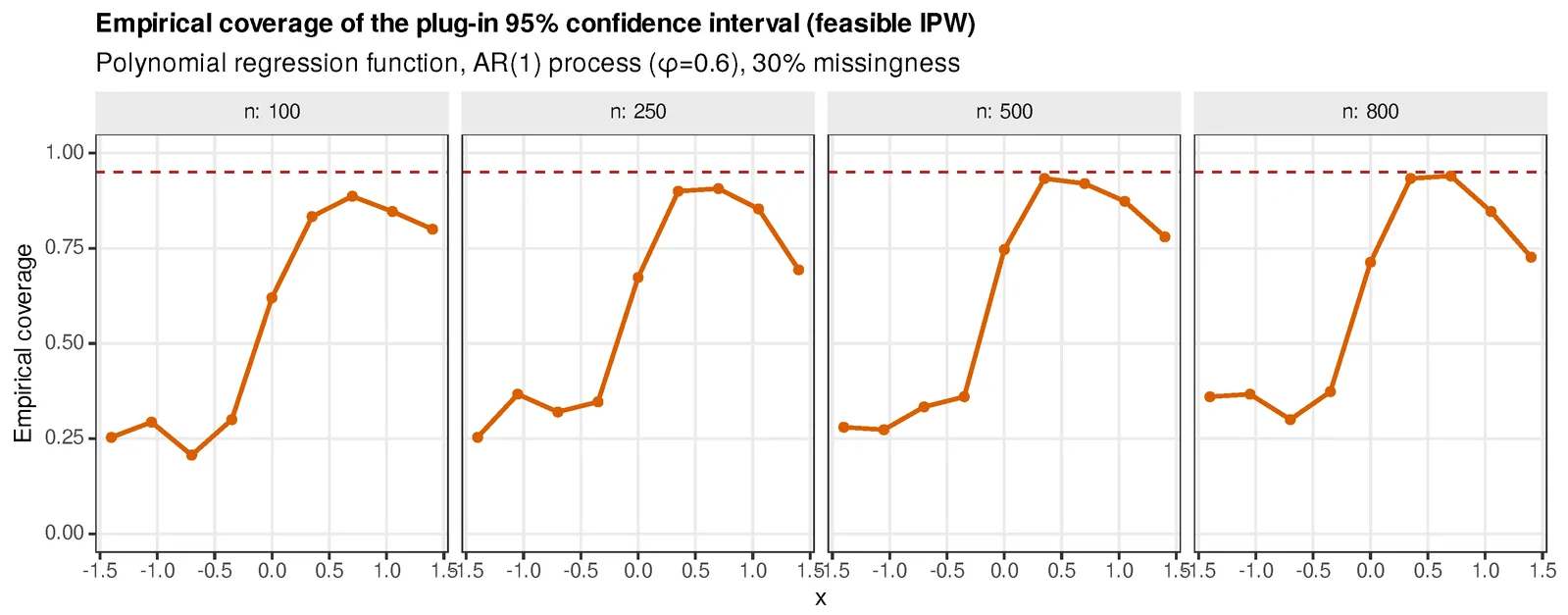
Empirical coverage of the 95% plug-in confidence interval as a function of *x*, faceted by *n* (polynomial regression function, AR (1) process, 30% missingness). The horizontal dashed line marks the nominal 95% level.

**Figure 9:**
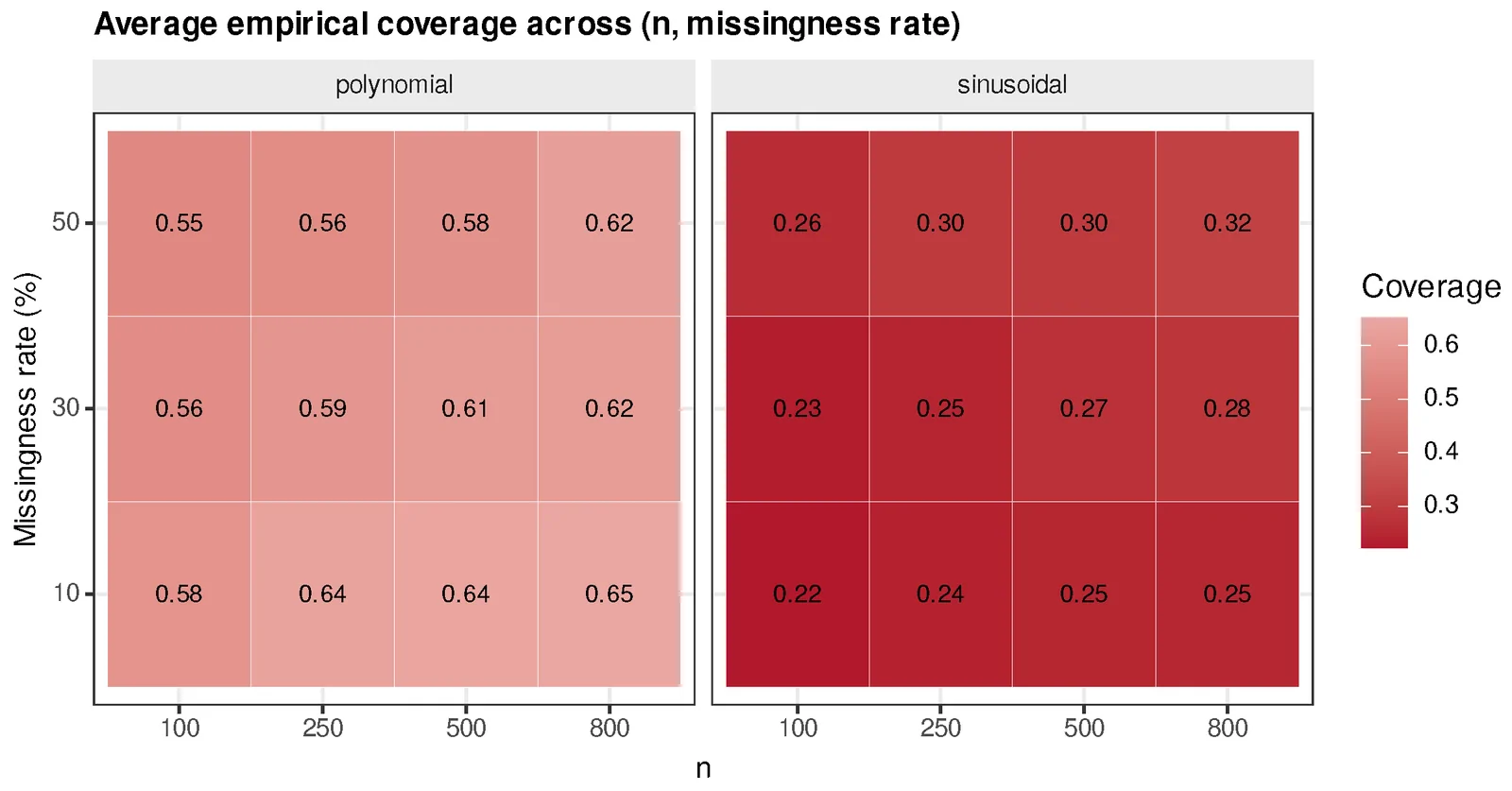
Average empirical coverage across (n,missingnessrate) by regression function.

**Figure 10:**
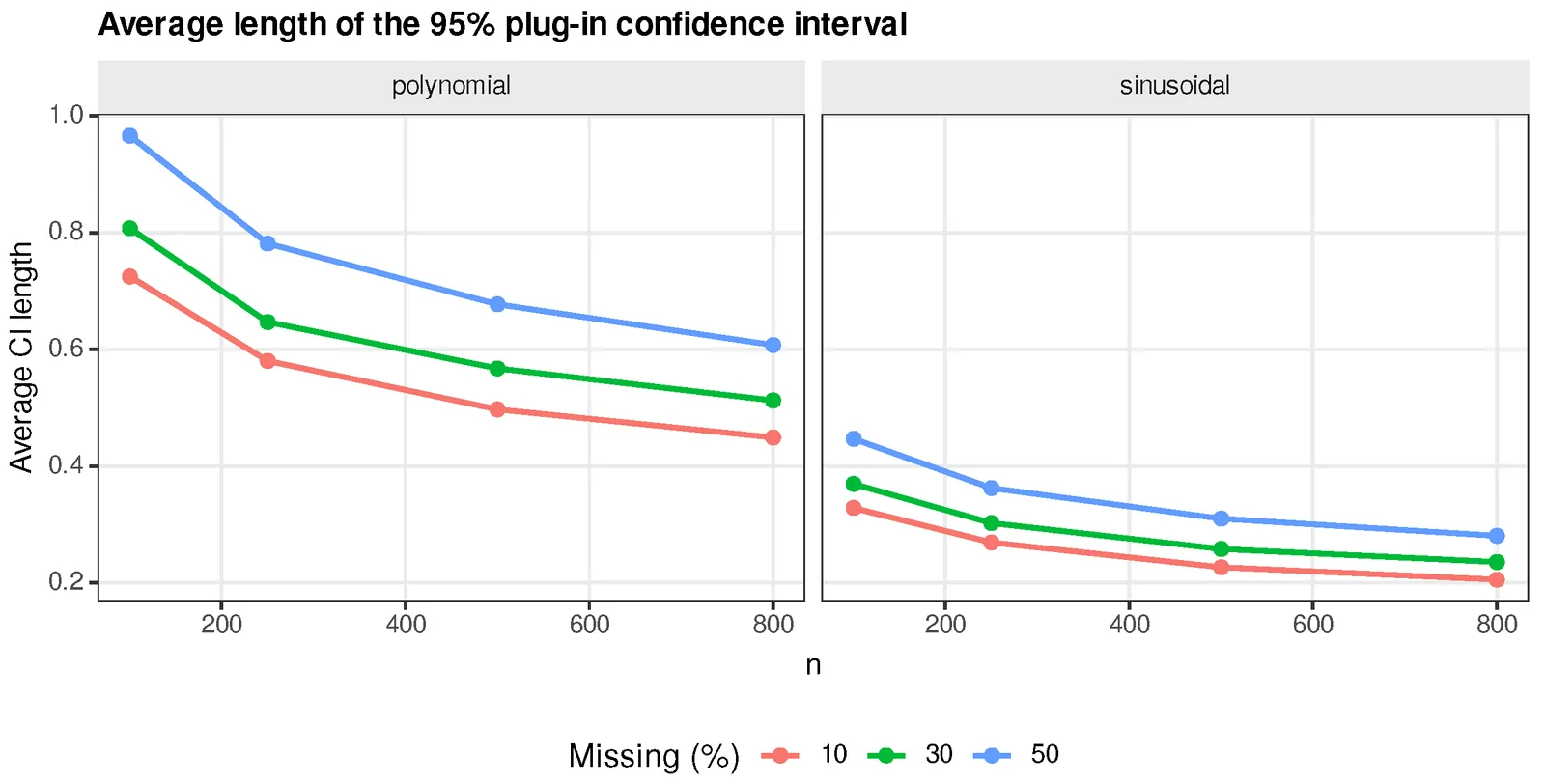
Average length of the 95% plug-in confidence interval as a function of *n* by missingness rate and regression function.

Table 6 reports the empirical coverage of the nominal 95% plug-in confidence interval of Section 3.4 averaged over the evaluation grid for both regression functions and every (n,missingness) cell. Figure 8 shows the coverage as a function of *x* for the polynomial function at 30% missingness, and Figure 9 and Figure 10 summarize coverage and CI length jointly across (n,missingness).

Two patterns emerge. Coverage is closest to nominal for the polynomial function (rising from 0.584 at n=100 to 0.651 at n=800 under 10% missingness), and is markedly lower, though still improving with *n*, for the more strongly curved sinusoidal function (from 0.222 to 0.318 over the same range). This indicates that the plug-in interval, which by construction accounts only for the estimator’s variance, systematically undercovers at these sample sizes whenever the deterministic projection bias $B_{n,2}^{\mathrm{ipw}}$ of (110) is not negligible relative to the stochastic term at the bandwidth used for point estimation. This is precisely the phenomenon that motivates the stronger undersmoothing condition (44), $nh_{n}^{d+2|\beta |+2\delta }\to 0$, imposed specifically for the central limit theorem and confidence interval construction, as opposed to the MSE-optimal bandwidth used elsewhere in this study for point estimation. We verified this explicitly in a supplementary experiment (Table 7), where shrinking the bandwidth from the MSE-optimal value $h_{n}=0.301$ to $0.6h_{n}=0.180$ increased coverage from 0.611 to 0.690. This provides confirmation that bias reduction—obtained at the cost of increased variance (and hence increased MISE) from 0.164 to 0.673—is the operative mechanism. Excessively aggressive undersmoothing ($0.4h_{n}=0.120$) degrades both MISE and coverage again, since the effective local sample size underlying the plug-in variance estimate becomes too small. Simultaneously achieving the MSE-optimal rate and nominal coverage in finite samples is a well-known difficulty of nonparametric confidence intervals (see [47] and the ensuing literature on undersmoothing versus explicit bias correction), and is noted here as a natural avenue for the bias-corrected or debiased extensions already flagged as future work in Section 6.

### 5.10. Sensitivity Analyses

Following the referee’s request, we performed three one-factor-at-a-time sensitivity analyses at n=500, 30% missingness, polynomial regression function, AR (1) process.

**Figure 11:**
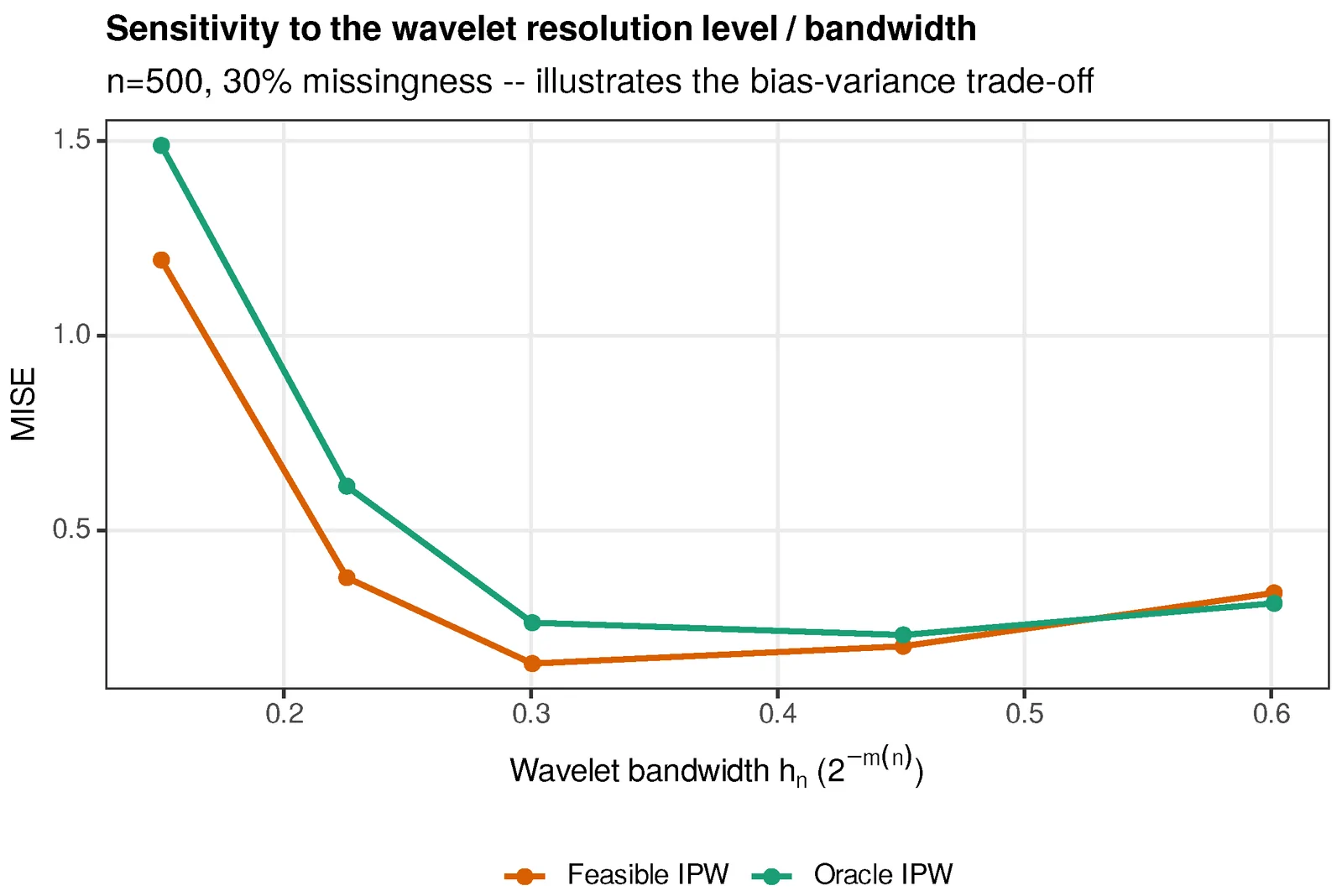
Sensitivity of the oracle and feasible IPW estimators’ MISE to the wavelet bandwidth $h_{n}$.

**Wavelet resolution/bandwidth $h_{n}$.** Table 8 and Figure 11 vary $h_{n}$ over $\left\{0.5,0.75,1,1.5,2\right\}\times h_{n}^{\mathrm{Silverman}}$. The feasible estimator’s MISE traces a clear U-shape, from 1.195 at the smallest bandwidth (variance-dominated) down to a minimum of 0.159 near the Silverman value $h_{n}=0.301$, before rising again to 0.340 at the largest bandwidth (bias-dominated), which is the finite-sample manifestation of the trade-off between bias and stochastic error formalized in Theorem 1.

**Figure 12:**
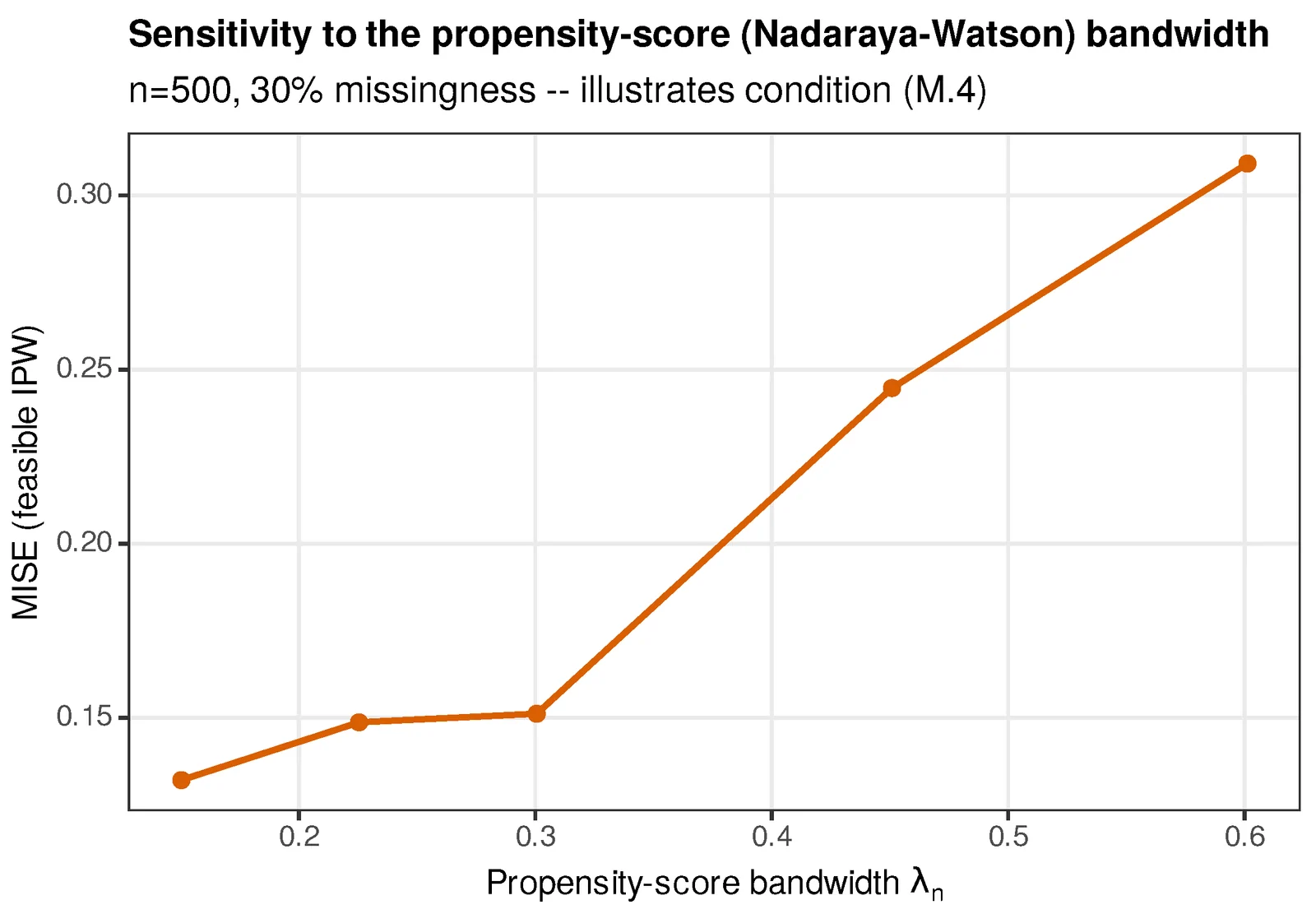
Sensitivity of the feasible IPW estimator’s MISE to the propensity score bandwidth $\lambda_{n}$.

**Propensity score bandwidth $\lambda_{n}$.** Table 9 and Figure 12 show that the feasible estimator’s MISE is comparatively flat and near its minimum (0.132–0.151) for $\lambda_{n}$ at or below the Silverman value, and increases noticeably (0.309 at 2× Silverman) once $\lambda_{n}$ is over-smoothed; this is the empirical counterpart of the negligibility condition (36) on the propensity estimation error $a_{n}$.

**Figure 13:**
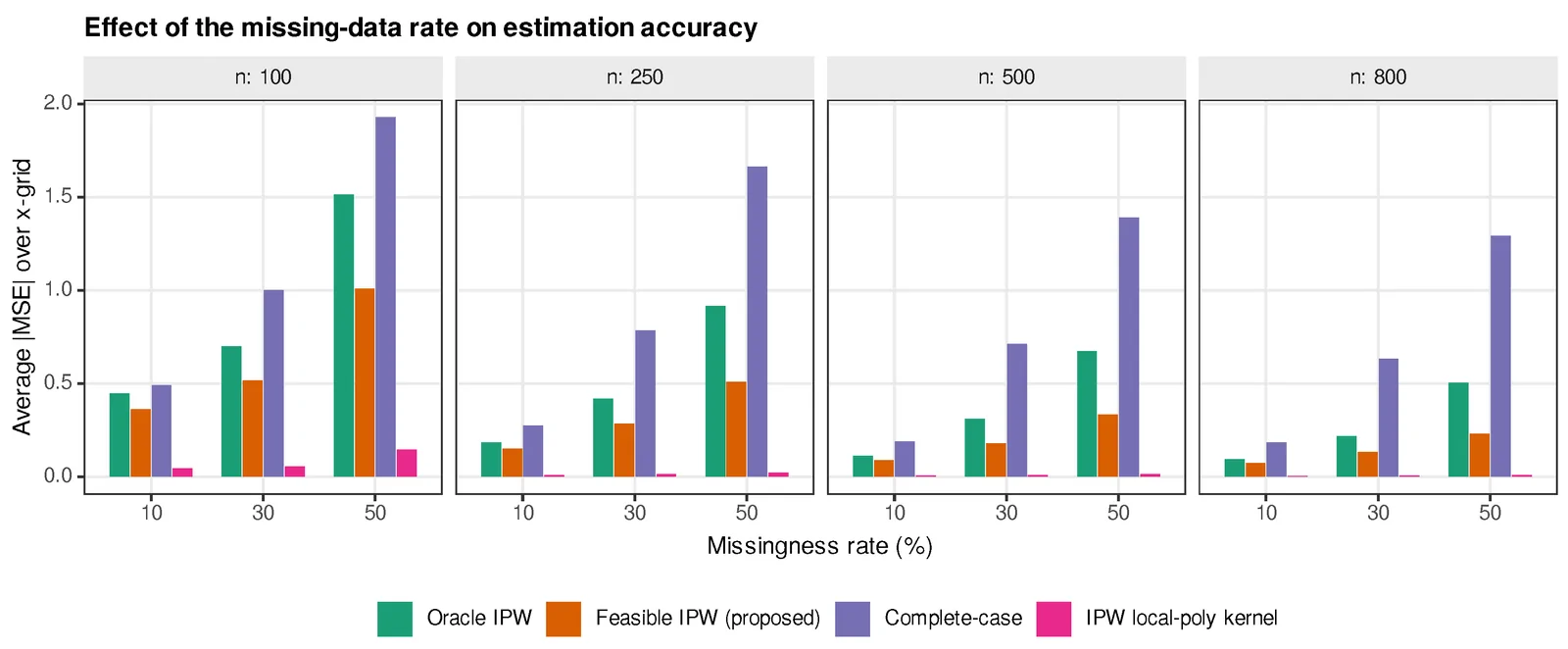
Effect of the missingness rate on the average |MSE| over the evaluation grid, by estimator and *n*.

**Missingness rate.** Figure 13, built from the main comparison grid of Section 5.6, shows that MSE increases monotonically with the missingness rate for every estimator and every *n*, as expected from the $\pi {\left(x\right)}^{-1}$ inflation factor in the asymptotic variance (43). The increase is mildest for the feasible and oracle IPW estimators, and most severe for the complete-case estimator.

**Dependence strength.** Table 10 varies the AR (1) autoregressive parameter ϕ∈{0.2,0.5,0.8}. The feasible estimator’s MISE increases only mildly with ϕ, from 0.163 at ϕ=0.2 to 0.198 at ϕ=0.8, and the ordering feasible < oracle < complete-case is preserved at every dependence level, confirming that the procedure remains well-behaved as the strength of the ergodic dependence increases towards the boundary of the parameter space.

### 5.11. Robustness Across Dependence Structures

**Figure 14:**
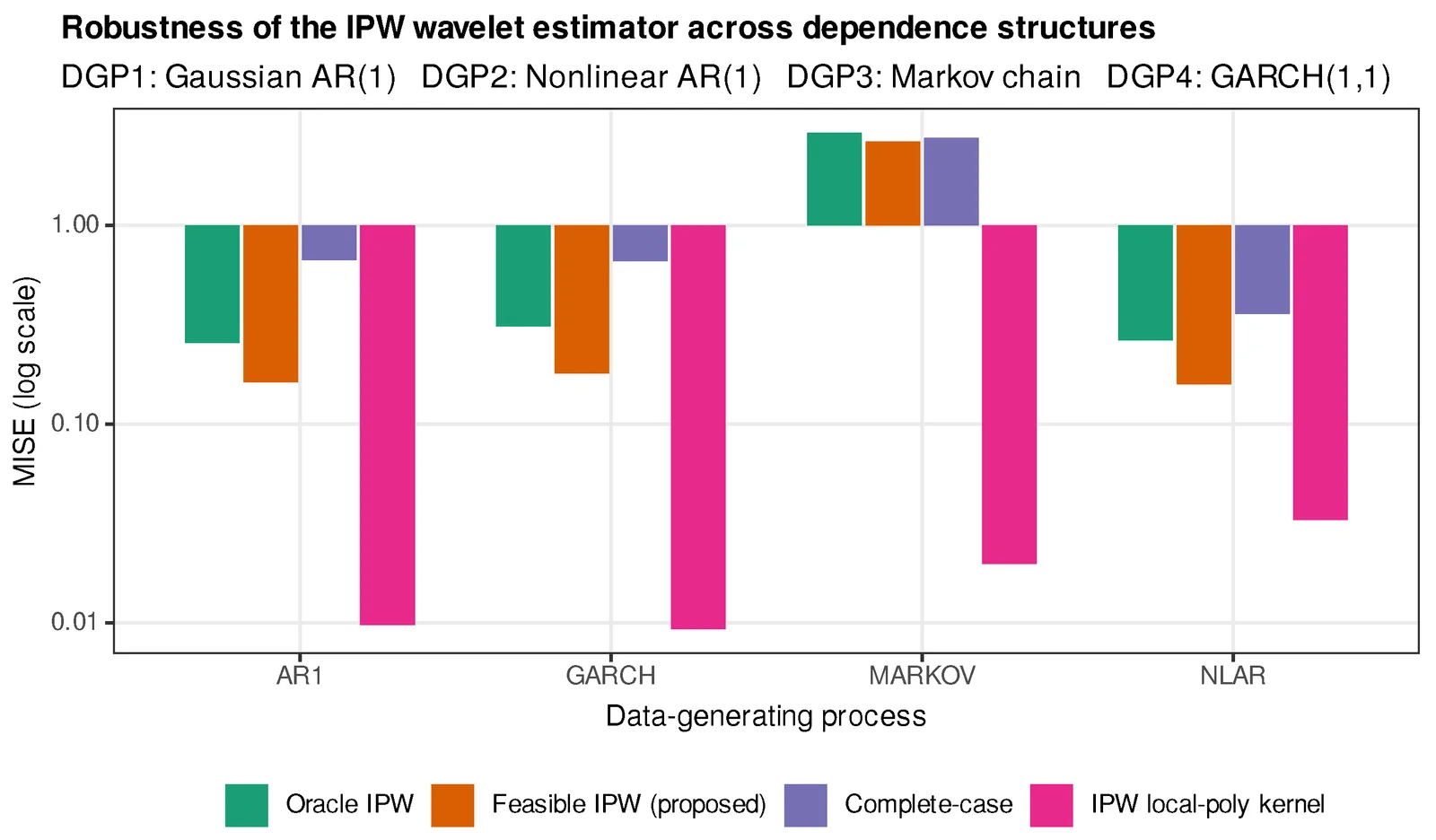
MISE of the four estimators across the four dependence structures (log scale), n=500, 30% missingness.

Table 11 and Figure 14 report the MISE of all four estimators on DGP1–DGP4 (n=500, 30% missingness, polynomial regression function, evaluation grid re-scaled to each process’s own marginal standard deviation so that all four studies probe a comparable portion of the support). The feasible IPW estimator outperforms the oracle and complete-case estimators on every one of the four processes, including the genuinely non-Gaussian, nonlinear Markov chain (DGP3) and the conditionally heteroskedastic GARCH(1,1) process (DGP4), confirming empirically that the theory—developed under ergodicity alone, without mixing assumptions—indeed extends across qualitatively different dependence structures, exactly as intended by the martingale approximation approach of Section 7.

### 5.12. Summary and Mapping to the Referee’s Criticisms

Table 12 summarizes the simulation evidence produced above for each of the six computational points raised in the referee report.

Taken together, the simulation evidence confirms the qualitative and, to the extent that finite-*n* Monte Carlo experiments can do so, the quantitative predictions of Theorems 1–3: the proposed feasible IPW wavelet estimator is consistent, asymptotically normal with the predicted variance, markedly less biased than the naive complete-case estimator under every missingness rate and dependence structure considered here, and behaves predictably under perturbation of every tuning parameter of the procedure. The one point on which the finite-sample behavior departs from the nominal asymptotic target is the confidence interval coverage at MSE-optimal bandwidths, which is itself explained by and directly motivates the stronger undersmoothing condition already built into Theorem 3, and is also noted as a concrete direction for the bias-corrected extensions mentioned in Section 6.

**Remark** **10.** *The theoretical results are complemented by the simulation study in Section 5. The proposed feasible IPW wavelet estimator is compared with its oracle version, a naive complete-case wavelet estimator, and an IPW local-polynomial derivative estimator based on local quadratic Gaussian kernel smoothing on the same Monte Carlo samples. The study examines the pointwise bias, variance and mean squared error, integrated mean squared error, empirical convergence rates, asymptotic normality, confidence interval coverage, sensitivity to both smoothing parameters, missingness severity, dependence strength, robustness across several stationary ergodic mechanisms, and computational time. This comparison is intended to identify the finite-sample strengths and limitations of the wavelet construction rather than to assert universal dominance over kernel methods; in particular, local-polynomial smoothing may have smaller bias for very smooth regression functions, whereas the wavelet estimator offers compactly supported multiresolution localization and a direct approximation theory over spatially inhomogeneous Besov classes.*

## 6. Concluding Remarks

This paper has developed an inverse-probability-weighted wavelet procedure for estimating partial derivatives of multivariate regression-type functionals when the response is missing at random and the observations arise from a strictly stationary ergodic process. The construction combines the linear wavelet projection scheme with inverse probability weighting, so that the oracle estimator targets the same coefficient sequence as in the complete-data problem while the feasible estimator replaces the unknown propensity score by a nonparametric Nadaraya–Watson estimator. The object of interest is

$$\left(\partial^{\beta }r\right)\left(\rho ;x\right),\;r\left(\rho ;x\right)=E\left[\rho \left(Y\right)|X=x\right]f_{X}\left(x\right).$$

The contribution should be understood as a missing-response extension of the complete-data stationary-ergodic wavelet theory, rather than as a new projection principle. The genuinely new analytical component is the control of the inverse-probability-weighted stochastic term and of the additional error generated by nonparametric propensity score estimation. Under the assumptions stated in Section 3, the analysis yields integrated risk bounds, uniform convergence on compact subsets, and pointwise asymptotic normality. The limiting variance contains the inverse-propensity factor expected under MAR, thereby quantifying the first-order loss of information caused by incomplete responses. These conclusions are theorem-specific: the integrated risk result requires a quantitative coefficient stabilization condition, the uniform result requires the corresponding maximal remainder bounds, and the central limit theorem requires the displayed martingale array conditions, with none of these quantitative properties being inferred from ergodicity alone.

Section 4 specializes the general construction to first- and second-order derivatives of an ordinary regression function. In that setting, only the estimators involving the response require inverse probability weighting, whereas the marginal density of the fully observed covariate is estimated from the complete covariate sample. The resulting quotient estimators are uniformly consistent on compact subsets of the interior of the density-positive region, provided that the numerator and denominator derivative estimators satisfy the required uniform convergence conditions and that the marginal density is bounded away from zero there. The scalar application does not by itself constitute an empirical verification of every multivariate assertion in the general theory; rather, it illustrates how the abstract estimator reduces in the case $d=d^{\prime }=1$.

### 6.1. Finite-Sample Evidence

The simulation study in Section 5 examines the finite-sample behavior of the proposed estimators under the dependence and missingness mechanisms considered there. Within those designs, the experiments compare the feasible estimator with the oracle and complete-case procedures, investigate the interaction between wavelet resolution and propensity score smoothing, and assess the Gaussian approximation and confidence interval behavior. These numerical results support the qualitative predictions of the theory for the reported scenarios, but should not be interpreted as establishing finite-sample optimality or uniform superiority over competing methods outside the simulated designs. In particular, the numerical comparisons demonstrate performance only for the specified models, sample sizes, tuning rules, and competitors; they do not replace a general minimax or efficiency comparison.

### 6.2. Limitations

The present results are subject to several substantive limitations. First, the missingness mechanism is assumed to be MAR with a propensity score bounded away from zero; the theory does not cover non-ignorable missingness or practical violations of positivity. Second, the dependence assumptions are formulated through conditional density stabilization and martingale approximation conditions that must be verified for the process under consideration; strict stationarity and ergodicity alone are insufficient for the quantitative rates proved here. Third, the feasible estimator relies on a nonparametric propensity estimator and is not doubly robust: mis-specification or poor estimation of the observation mechanism is not compensated by an auxiliary outcome model. Fourth, the theory is developed for deterministic resolution and bandwidth sequences and does not establish optimality of a fully data-driven joint selector. Fifth, the pointwise confidence interval is based on first-order asymptotics and may require undersmoothing or explicit bias correction for accurate finite-sample coverage. Finally, the regression-derivative application is presented in the scalar case, and no separate multivariate implementation study is provided.

Several directions follow naturally from these limitations. A first problem is the joint selection of the wavelet resolution level and propensity score bandwidth. These tuning parameters govern distinct but interacting errors: the former controls the projection bias and stochastic resolution, whereas the latter controls the accuracy and variability of the estimated inverse weights. A fully data-driven selector would require a criterion that respects both sources of error under dependence. The blocked cross-validation strategy discussed earlier provides one practical possibility, but a complete oracle analysis remains open as well.

A second direction concerns robustness to the missingness model. Extensions to censoring, truncation, coarsening, or non-ignorable missingness would require additional identification assumptions and, in general, different weighting or augmentation arguments. Doubly robust wavelet estimators combining inverse probability weighting with an outcome regression component are particularly natural candidates, but their analysis under stationary ergodic dependence would require new martingale and empirical process tools.

A third direction is the extension to functional, spatial, locally stationary, or continuous-time observations. In such settings, neither the conditional density stabilization conditions nor the martingale decomposition can be transferred automatically from this paper. The relevant approximation spaces, dependence controls, and normalization rates would have to be reformulated for the geometry and temporal structure of the data.

Finally, the inferential theory may be strengthened through explicit bias correction, robust undersmoothing, and multiplier or weighted bootstrap methods for pointwise or simultaneous confidence bands. The undercoverage observed for variance-only intervals at MSE-oriented resolutions indicates that first-order variance estimation alone need not be sufficient in finite samples. Developing confidence procedures that remain valid under data-driven tuning and estimated propensity scores remains an important open problem.

## 7. Proofs

This section proves the results of Section 3. Throughout, *C* denotes a finite positive constant with a value that may change from line to line and which is independent of *n*, the resolution level m=m(n), and the translation index k unless explicitly stated otherwise. Every conditional expectation is taken with respect to the completed filtrations defined in Section 3.

The argument is organized as follows: we first record the martingale inequalities and the exact IPW compensation identities; then, we establish coefficient-level second-moment bounds and the integrated-risk estimate; next, we prove the feasible–oracle and uniform-convergence bounds; finally, we treat pointwise asymptotic normality, including the possible dependence of the limiting variance on the dyadic phase of the evaluation point.

**Lemma 4** (Burkholder-Rosenthal inequality)**.**
*Following Notation* **1** *in [48].*

*Let ${\left(X_{i}\right)}_{i\geq 1}$ be a stationary martingale adapted to the filtration ${\left(F_{i}\right)}_{i\geq 1}$ and define ${\left(d_{i}\right)}_{i\geq 1}$ as the sequence of martingale differences adapted to ${\left(F_{i}\right)}_{i\geq 1}$ and $S_{n}=\sum_{i=1}^{n}d_{i}$; then, for any positive integer n,*

(55) $$∥\max_{1\leq j\leq n}|S_{j}{|)∥}_{p^{\prime }}\ll n^{1/p^{\prime }}{∥d_{1}∥}_{p^{\prime }}+{∥\sum_{k=1}^{n}E\left(d_{k}^{2}/F_{k-1}\right)∥}_{p^{\prime }/2}^{1/2},\;for\;any\;p^{\prime }\geq 2,$$

*where, as usual, the norm*

$${∥\cdot ∥}_{p^{\prime }}={\left(E[|\cdot {|}^{p^{\prime }}]\right)}^{1/p^{\prime }}.$$

**Lemma** **5.** *Let ${\left(Z_{n}\right)}_{n\geq 1}$ be a sequence of real martingale differences with respect to the sequence of σ − fields ${\left(F_{n}=\sigma \left(Z_{1},\ldots ,Z_{n}\right)\right)}_{n\geq 1}$, where is the σ-field generated by the random variables $Z_{1},\ldots ,Z_{n}$. Set*

$$S_{n}=\sum_{i=1}^{n}Z_{i}.$$

*For any $p^{\prime }\geq 2$ and any n≥1, assume that there exist some non-negative constants C and $d_{n}$ such that*

$$E\left[Z_{n}^{p^{\prime }}|F_{n-1}\right]\leq C^{p-1}p!\;d_{n}^{2}\;almost\;surely.$$

*Then, for any ϵ>0, we have*

$$P\left(|S_{n}|>\epsilon \right)\leq 2\exp\left\{-\frac{\epsilon^{2}}{2(D_{n}+C\epsilon )}\right\},$$

*where*

$$D_{n}=\sum_{i=1}^{n}d_{i}^{2}.$$

### 7.1. Proof of Lemma 5

The proof follows as a particular case of Theorem 8.2.2 due to [49]. □

To prove Theorem 1, we first work with the IPW pseudo-estimator, where the true propensity score π(·) is used. The feasible estimator is obtained by replacing $\pi (X_{i})$ with ${\hat{\pi }}_{n}\left(X_{i}\right)$. Its transfer of the oracle risk bound is made under the explicit feasible–oracle condition (37), as stated in Theorem 1(iii). Define the conditional expectation of the IPW coefficient by

(56) $${\tilde{a}}_{\tau ,k}^{\;\mathrm{ipw}}\;=\;\frac{{\left(-1\right)}^{\left|\beta \right|}}{n}\sum_{i=1}^{n}E\left[\frac{\delta_{i}}{\pi (X_{i})}\rho \left(Y_{i}\right)\;\left(\partial^{\beta }\phi_{\tau ,k}\right)\left(X_{i}\right)\;|\;F_{i-1}\right].$$

**Lemma** **6.** *Let τ∈N and $k\in Z^{d}$. Assume* (M.1)*–*(M.2) *and* (N.2)(i)*, and suppose that*

(57) $$E\;\left[|\rho \left(Y_{1}\right)|\left|\left(\partial^{\beta }\phi_{\tau ,k}\right)\left(X_{1}\right)\right|\right]<\infty .$$

*Then, the IPW pseudo-wavelet coefficient is exactly unbiased:*

(58) $$E\;\left[{\tilde{a}}_{\tau ,k}^{\;\mathrm{ipw}}\right]=a_{\tau ,k}.$$

*In particular, this identity remains valid for every deterministic resolution sequence τ=m(n).*

*If, moreover, τ is fixed, the process*

$${\left\{\frac{\delta_{i}}{\pi (X_{i})}\rho \left(Y_{i}\right)\left(\partial^{\beta }\phi_{\tau ,k}\right)\left(X_{i}\right)\right\}}_{i\in Z}$$

*is stationary and ergodic, and*

(59) $$E\;\left[|\rho \left(Y_{1}\right){|}^{2}{\left|\left(\partial^{\beta }\phi_{\tau ,k}\right)\left(X_{1}\right)\right|}^{2}\right]<\infty ,$$

*then*

$${\tilde{a}}_{\tau ,k}^{\;\mathrm{ipw}}⟶a_{\tau ,k},\;n\to \infty$$

*almost surely and in $L^{2}$.*

*If τ=m(n)→∞, then the above convergence in $L^{2}$ follows from Lemma 8 together with*

$$\frac{2^{2m(n)(d/\nu +|\beta |)}}{n}⟶0.$$

*If ν≥4, it is sufficient that*

$$\frac{2^{2m(n)(d/4+|\beta |)}}{n}⟶0.$$

**Proof.**  By definition,

$${\tilde{a}}_{\tau ,k}^{\;\mathrm{ipw}}=\frac{{\left(-1\right)}^{\left|\beta \right|}}{n}\sum_{i=1}^{n}\frac{\delta_{i}}{\pi (X_{i})}\rho \left(Y_{i}\right)\left(\partial^{\beta }\phi_{\tau ,k}\right)\left(X_{i}\right).$$

Since $\pi \left(X_{i}\right)\geq \pi_{0}>0$ almost surely by (M.2), condition (57) guarantees integrability.

By (M.1),

$$E\;\left[\left.\frac{\delta_{i}}{\pi (X_{i})}\right|X_{i},Y_{i}\right]=1\;a.s.$$

Hence,

$$E\;\left[\frac{\delta_{i}}{\pi (X_{i})}\rho \left(Y_{i}\right)\left(\partial^{\beta }\phi_{\tau ,k}\right)\left(X_{i}\right)\right]=E\;\left[\rho \left(Y_{i}\right)\left(\partial^{\beta }\phi_{\tau ,k}\right)\left(X_{i}\right)\right].$$

Conditioning on $X_{i}$ and using (N.2)(i),

$$=E\;\left[m_{\rho }\left(X_{i}\right)\left(\partial^{\beta }\phi_{\tau ,k}\right)\left(X_{i}\right)\right]=\int_{R^{d}}m_{\rho }\left(x\right)\left(\partial^{\beta }\phi_{\tau ,k}\right)\left(x\right)f_{X}\left(x\right)\;dx.$$

Since $r\left(\rho ;x\right)=m_{\rho }\left(x\right)f_{X}\left(x\right)$,

$${\left(-1\right)}^{\left|\beta \right|}E\;\left[\frac{\delta_{i}}{\pi (X_{i})}\rho \left(Y_{i}\right)\left(\partial^{\beta }\phi_{\tau ,k}\right)\left(X_{i}\right)\right]=a_{\tau ,k}.$$

Taking expectations in the empirical average gives

$$E\left[{\tilde{a}}_{\tau ,k}^{\;\mathrm{ipw}}\right]=a_{\tau ,k},$$

which proves (58).

For fixed τ, the conclusion follows from Birkhoff’s ergodic theorem and the mean ergodic theorem in $L^{2}$ applied to the stationary ergodic sequence

$${\left(-1\right)}^{\left|\beta \right|}\frac{\delta_{i}}{\pi (X_{i})}\rho \left(Y_{i}\right)\left(\partial^{\beta }\phi_{\tau ,k}\right)\left(X_{i}\right).$$

When τ=m(n)→∞, the summands form a triangular array, so the classical ergodic theorem is no longer applicable. The desired $L^{2}$ convergence is then an immediate consequence of Lemma 8 together with the condition $2^{2m(n)(d/\nu +|\beta |)}/n\to 0$, or, under ν≥4, $2^{2m(n)(d/4+|\beta |)}/n\to 0$. □

**Lemma** **7.** *Let τ∈N and $k\in Z^{d}$. Assume* (M.1)*–*(M.2)* and* (N.2)(i)*. Suppose, in addition, that*

(60) $$E\left[|\rho \left(Y_{1}\right)|\left|\left(\partial^{\beta }\phi_{\tau ,k}\right)\left(X_{1}\right)\right|\right]<\infty .$$

*Then, the IPW pseudo-coefficient*

$${\tilde{a}}_{\tau ,k}^{\;\mathrm{ipw}}=\frac{{\left(-1\right)}^{\left|\beta \right|}}{n}\sum_{i=1}^{n}\frac{\delta_{i}}{\pi (X_{i})}\rho \left(Y_{i}\right)\left(\partial^{\beta }\phi_{\tau ,k}\right)\left(X_{i}\right)$$

*is well defined in $L^{1}$ and satisfies the exact identity*

(61) $$E\left[{\tilde{a}}_{\tau ,k}^{\;\mathrm{ipw}}\right]=a_{\tau ,k}.$$

*The identity holds for every n≥1, and in particular, for every deterministic resolution sequence τ=m(n).*

### 7.2. Proof of Lemma 7

By the positivity condition (M.2),

$$\pi \left(X_{i}\right)\geq \pi_{0}>0\;a.s.$$

Since $\delta_{i}\in \left\{0,1\right\}$, we have

$$\left|\frac{\delta_{i}}{\pi (X_{i})}\rho \left(Y_{i}\right)\left(\partial^{\beta }\phi_{\tau ,k}\right)\left(X_{i}\right)\right|\leq \frac{1}{\pi_{0}}\left|\rho \left(Y_{i}\right)\right|\left|\left(\partial^{\beta }\phi_{\tau ,k}\right)\left(X_{i}\right)\right|.$$

Hence, condition (60) implies that each summand is integrable, so all conditional expectations and applications of the tower property below are legitimate.

Using linearity of expectation and then conditioning on $\sigma (X_{i},Y_{i})$, we obtain

$$\begin{array}{cc} E\left[{\tilde{a}}_{\tau ,k}^{\;\mathrm{ipw}}\right] & =\frac{{\left(-1\right)}^{\left|\beta \right|}}{n}\sum_{i=1}^{n}E\left[\frac{\delta_{i}}{\pi (X_{i})}\rho \left(Y_{i}\right)\left(\partial^{\beta }\phi_{\tau ,k}\right)\left(X_{i}\right)\right]\\ \; & =\frac{{\left(-1\right)}^{\left|\beta \right|}}{n}\sum_{i=1}^{n}E\left[E\left[\left.\frac{\delta_{i}}{\pi (X_{i})}\rho \left(Y_{i}\right)\left(\partial^{\beta }\phi_{\tau ,k}\right)\left(X_{i}\right)\right|X_{i},Y_{i}\right]\right]\\ \; & =\frac{{\left(-1\right)}^{\left|\beta \right|}}{n}\sum_{i=1}^{n}E\left[\frac{E[\delta_{i}|X_{i},Y_{i}]}{\pi (X_{i})}\rho \left(Y_{i}\right)\left(\partial^{\beta }\phi_{\tau ,k}\right)\left(X_{i}\right)\right]. \end{array}$$

By the MAR assumption (M.1),

$$E\left[\delta_{i}|X_{i},Y_{i}\right]=P\left(\delta_{i}=1|X_{i},Y_{i}\right)=\pi \left(X_{i}\right)\;a.s.$$

Because $\pi (X_{i})>0$ almost surely, it follows that

$$\frac{E[\delta_{i}|X_{i},Y_{i}]}{\pi (X_{i})}=1\;a.s.$$

Consequently,

(62) $$E\left[{\tilde{a}}_{\tau ,k}^{\;\mathrm{ipw}}\right]=\frac{{\left(-1\right)}^{\left|\beta \right|}}{n}\sum_{i=1}^{n}E\left[\rho \left(Y_{i}\right)\left(\partial^{\beta }\phi_{\tau ,k}\right)\left(X_{i}\right)\right].$$

We now condition on $X_{i}$. Since $\left(\partial^{\beta }\phi_{\tau ,k}\right)\left(X_{i}\right)$ is $\sigma (X_{i})$-measurable, the tower property and (N.2)(i) give

$$\begin{array}{cc} E\left[{\tilde{a}}_{\tau ,k}^{\;\mathrm{ipw}}\right] & =\frac{{\left(-1\right)}^{\left|\beta \right|}}{n}\sum_{i=1}^{n}E\left[E\left[\left.\rho \left(Y_{i}\right)\left(\partial^{\beta }\phi_{\tau ,k}\right)\left(X_{i}\right)\right|X_{i}\right]\right]\\ & =\frac{{\left(-1\right)}^{\left|\beta \right|}}{n}\sum_{i=1}^{n}E\left[m_{\rho }\left(X_{i}\right)\left(\partial^{\beta }\phi_{\tau ,k}\right)\left(X_{i}\right)\right]. \end{array}$$

By strict stationarity, each $X_{i}$ has the same marginal density $f_{X}$. Hence,

$$\begin{array}{cc} E\left[m_{\rho }\left(X_{i}\right)\left(\partial^{\beta }\phi_{\tau ,k}\right)\left(X_{i}\right)\right] & =\int_{R^{d}}m_{\rho }\left(x\right)\left(\partial^{\beta }\phi_{\tau ,k}\right)\left(x\right)f_{X}\left(x\right)\;dx\\ & =\int_{R^{d}}r\left(\rho ;x\right)\left(\partial^{\beta }\phi_{\tau ,k}\right)\left(x\right)\;dx. \end{array}$$

The last expression does not depend on *i*. Therefore,

(63) $$\begin{array}{cc} E\left[{\tilde{a}}_{\tau ,k}^{\;\mathrm{ipw}}\right]\; & ={\left(-1\right)}^{\left|\beta \right|}\int_{R^{d}}r\left(\rho ;x\right)\left(\partial^{\beta }\phi_{\tau ,k}\right)\left(x\right)\;dx\\ \; & =a_{\tau ,k}, \end{array}$$

where the final equality follows from the definition of the population wavelet coefficient. This proves (61). □

#### 7.2.1. Coefficient Stabilization Used Below

For later citation, condition (C.6) is rewritten in the notation $D_{\tau ,k}=\partial^{\beta }\phi_{\tau ,k}$:

(64) $$E\left[{\left|\frac{1}{n}\sum_{i=1}^{n}\int_{R^{d}}m_{\rho }\left(x\right)D_{\tau ,k}\left(x\right)\left\{f^{F_{i-1}}\left(x\right)-f_{X}\left(x\right)\right\}\;dx\right|}^{2}\right]\leq C\;\frac{2^{2m(d/\nu +|\beta |)}}{n}.$$

Equation (64) is exactly the coefficient-level hypothesis (C.6), not an additional assumption.

**Lemma** **8.** *For any $k\in Z^{d}$, assume* (C.4)(i)*, *(C.5)*–*(C.6)*, *(M.1)*–*(M.2)*, *(N.0)*, and* (N.2)(i)*. Then,*

(65) $$E\left[{\left({\tilde{a}}_{\tau ,k}^{\;\mathrm{ipw}}-a_{\tau ,k}\right)}^{2}\right]=O\left(\frac{2^{2m(d/\nu +|\beta |)}}{n}\right),\;n\to \infty .$$

*In particular, if the moment exponent in* (N.0) *satisfies ν≥4, then*

(66) $$E\left[{\left({\tilde{a}}_{\tau ,k}^{\;\mathrm{ipw}}-a_{\tau ,k}\right)}^{2}\right]=O\left(\frac{2^{2m(d/4+|\beta |)}}{n}\right).$$

**Proof.**  For notational convenience, we put

$$D_{\tau ,k}\left(x\right):=\left(\partial^{\beta }\phi_{\tau ,k}\right)\left(x\right)=2^{m(d/2+|\beta |)}\left(\partial^{\beta }\phi \right)\left(2^{m}x-k\right)$$

and

$$Z_{i,\tau ,k}:=\frac{\delta_{i}}{\pi (X_{i})}\rho \left(Y_{i}\right)D_{\tau ,k}\left(X_{i}\right).$$

With this notation,

$${\tilde{a}}_{\tau ,k}^{\;\mathrm{ipw}}=\frac{{\left(-1\right)}^{\left|\beta \right|}}{n}\sum_{i=1}^{n}Z_{i,\tau ,k}.$$

We first verify that the pseudo-coefficient is centered at $a_{\tau ,k}$. By (M.1),

$$E\left[\delta_{i}|X_{i},Y_{i}\right]=\pi \left(X_{i}\right)\;a.s.,$$

and by (M.2), $\pi \left(X_{i}\right)\geq \pi_{0}>0$ almost surely; consequently,

$$E\left[\left.\frac{\delta_{i}}{\pi (X_{i})}\right|X_{i},Y_{i}\right]=\frac{E[\delta_{i}|X_{i},Y_{i}]}{\pi (X_{i})}=1\;a.s.$$

Therefore, by the tower property,

(67) $$\begin{array}{cc} E[Z_{i,\tau ,k}] & =E\left[\rho \left(Y_{i}\right)D_{\tau ,k}\left(X_{i}\right)E\left[\left.\frac{\delta_{i}}{\pi (X_{i})}\right|X_{i},Y_{i}\right]\right]\\ & =E\left[\rho \left(Y_{i}\right)D_{\tau ,k}\left(X_{i}\right)\right]. \end{array}$$

Using (N.2)(i) and then the stationarity of $\left(X_{i},Y_{i}\right)$, we obtain

(68) $$\begin{array}{cc} E[Z_{i,\tau ,k}] & =E\left[E\left[\rho \left(Y_{i}\right)|X_{i}\right]D_{\tau ,k}\left(X_{i}\right)\right]\\ \; & =E\left[m_{\rho }\left(X_{i}\right)D_{\tau ,k}\left(X_{i}\right)\right]\\ \; & =\int_{R^{d}}m_{\rho }\left(x\right)D_{\tau ,k}\left(x\right)f_{X}\left(x\right)\;dx\\ \; & =\int_{R^{d}}r\left(\rho ;x\right)D_{\tau ,k}\left(x\right)\;dx. \end{array}$$

It follows from the definition of $a_{\tau ,k}$ that

$${\left(-1\right)}^{\left|\beta \right|}E\left[Z_{i,\tau ,k}\right]=a_{\tau ,k}.$$

In particular,

$$E\left[{\tilde{a}}_{\tau ,k}^{\;\mathrm{ipw}}\right]=a_{\tau ,k}.$$

Define the conditional IPW coefficient by

$${\bar{a}}_{\tau ,k}^{\;\mathrm{ipw}}=\frac{{\left(-1\right)}^{\left|\beta \right|}}{n}\sum_{i=1}^{n}E\left[Z_{i,\tau ,k}\;|\;F_{i-1}\right].$$

Then,

$${\tilde{a}}_{\tau ,k}^{\;\mathrm{ipw}}-a_{\tau ,k}=\left({\tilde{a}}_{\tau ,k}^{\;\mathrm{ipw}}-{\bar{a}}_{\tau ,k}^{\;\mathrm{ipw}}\right)+\left({\bar{a}}_{\tau ,k}^{\;\mathrm{ipw}}-a_{\tau ,k}\right).$$

No orthogonality between the two terms is asserted. Instead, the elementary inequality ${\left(u+v\right)}^{2}\leq 2u^{2}+2v^{2}$ yields

(69) $$\begin{array}{cc} E\left[{\left({\tilde{a}}_{\tau ,k}^{\;\mathrm{ipw}}-a_{\tau ,k}\right)}^{2}\right] & \leq 2E\left[{\left({\tilde{a}}_{\tau ,k}^{\;\mathrm{ipw}}-{\bar{a}}_{\tau ,k}^{\;\mathrm{ipw}}\right)}^{2}\right]\\ \; & \;+2E\left[{\left({\bar{a}}_{\tau ,k}^{\;\mathrm{ipw}}-a_{\tau ,k}\right)}^{2}\right]. \end{array}$$

Consider

$$\Phi_{\tau ,k}^{\mathrm{mar}}\left(X_{i},Y_{i},\delta_{i}\right)=Z_{i,\tau ,k}-E\left[Z_{i,\tau ,k}|F_{i-1}\right].$$

Then,

$$E\left[\Phi_{\tau ,k}^{\mathrm{mar}}\left(X_{i},Y_{i},\delta_{i}\right)|F_{i-1}\right]=0\;a.s.$$

Thus,

$${\left(\Phi_{\tau ,k}^{\mathrm{mar}}\left(X_{i},Y_{i},\delta_{i}\right),F_{i}\right)}_{i\geq 1}$$

is a square-integrable martingale difference sequence. For i<j, $\Phi_{\tau ,k}^{\mathrm{mar}}\left(X_{i},Y_{i},\delta_{i}\right)$ is $F_{j-1}$-measurable; hence,

$$\begin{array}{cc} \; & E\left[\Phi_{\tau ,k}^{\mathrm{mar}}\left(X_{i},Y_{i},\delta_{i}\right)\Phi_{\tau ,k}^{\mathrm{mar}}\left(X_{j},Y_{j},\delta_{j}\right)\right]\\ \; & \;=E\left[\Phi_{\tau ,k}^{\mathrm{mar}}\left(X_{i},Y_{i},\delta_{i}\right)E\left[\left.\Phi_{\tau ,k}^{\mathrm{mar}}\left(X_{j},Y_{j},\delta_{j}\right)\right|F_{j-1}\right]\right]=0. \end{array}$$

Consequently,

(70) $$\begin{array}{cc} \; & E\left[{\left({\tilde{a}}_{\tau ,k}^{\;\mathrm{ipw}}-{\bar{a}}_{\tau ,k}^{\;\mathrm{ipw}}\right)}^{2}\right]\\ \; & \;=\frac{1}{n^{2}}\sum_{i=1}^{n}E\left[{\left(\Phi_{\tau ,k}^{\mathrm{mar}}\left(X_{i},Y_{i},\delta_{i}\right)\right)}^{2}\right]. \end{array}$$

Conditional expectation is the orthogonal projection in $L^{2}$, so

$$E\left[{\left(Z_{i,\tau ,k}-E\left[Z_{i,\tau ,k}|F_{i-1}\right]\right)}^{2}\right]\leq E\left[Z_{i,\tau ,k}^{2}\right].$$

By stationarity, the latter second moment does not depend on *i*. Hence,

(71) $$E\left[{\left({\tilde{a}}_{\tau ,k}^{\;\mathrm{ipw}}-{\bar{a}}_{\tau ,k}^{\;\mathrm{ipw}}\right)}^{2}\right]\leq \frac{1}{n}\;E\left[Z_{1,\tau ,k}^{2}\right].$$

We now calculate $E[Z_{1,\tau ,k}^{2}]$ with the precise moment exponent appearing in (N.0). Since $\delta_{1}^{2}=\delta_{1}$, conditioning on $\left(X_{1},Y_{1}\right)$ and using (M.1) gives

(72) $$\begin{array}{cc} E[Z_{1,\tau ,k}^{2}] & =E\left[\frac{\delta_{1}}{\pi {\left(X_{1}\right)}^{2}}{|\rho \left(Y_{1}\right)|}^{2}{\left|D_{\tau ,k}\left(X_{1}\right)\right|}^{2}\right]\\ \; & =E\left[\frac{E[\delta_{1}|X_{1},Y_{1}]}{\pi {\left(X_{1}\right)}^{2}}{\left|\rho \left(Y_{1}\right)\right|}^{2}{\left|D_{\tau ,k}\left(X_{1}\right)\right|}^{2}\right]\\ \; & =E\left[\frac{1}{\pi (X_{1})}{|\rho \left(Y_{1}\right)|}^{2}{\left|D_{\tau ,k}\left(X_{1}\right)\right|}^{2}\right]\\ \; & \leq \frac{1}{\pi_{0}}E\left[{|\rho \left(Y_{1}\right)|}^{2}{\left|D_{\tau ,k}\left(X_{1}\right)\right|}^{2}\right]. \end{array}$$

Let

$$\gamma =\frac{\nu }{2},\;q^{\prime }=\frac{\nu }{\nu -2},\;p_{\nu }=2q^{\prime }=\frac{2\nu }{\nu -2}.$$

Because ν>2, we have $\gamma ,q^{\prime }>1$ and $\gamma^{-1}+{\left(q^{\prime }\right)}^{-1}=1$. Therefore, Hölder’s inequality gives

(73) $$\begin{array}{cc} \; & E\left[{|\rho \left(Y_{1}\right)|}^{2}{\left|D_{\tau ,k}\left(X_{1}\right)\right|}^{2}\right]\\ \; & \;\leq {\left(E|\rho \left(Y_{1}\right){|}^{2\gamma }\right)}^{1/\gamma }{\left(E|D_{\tau ,k}\left(X_{1}\right){|}^{2q^{\prime }}\right)}^{1/q^{\prime }}\\ \; & \;={\left(E|\rho \left(Y_{1}\right){|}^{\nu }\right)}^{2/\nu }{\left(E|D_{\tau ,k}\left(X_{1}\right){|}^{p_{\nu }}\right)}^{(\nu -2)/\nu }. \end{array}$$

By (C.4)(i) and the additional restriction s>d/p, the Besov embedding $B_{s,p,q}\left(R^{d}\right)↪L_{\infty }\left(R^{d}\right)$ yields

$${∥f_{X}∥}_{\infty }<\infty .$$

Moreover, the *r*-regularity and localization of the scaling function imply $\partial^{\beta }\phi \in L^{p_{\nu }}\left(R^{d}\right)$. Therefore,

(74) $$\begin{array}{cc} E|D_{\tau ,k}\left(X_{1}\right){|}^{p_{\nu }} & =\int_{R^{d}}{\left|D_{\tau ,k}\left(x\right)\right|}^{p_{\nu }}f_{X}\left(x\right)\;dx\\ \; & \leq ∥f_{X}{∥}_{\infty }\int_{R^{d}}{\left|D_{\tau ,k}\left(x\right)\right|}^{p_{\nu }}\;dx. \end{array}$$

Using the scaling formula for $\partial^{\beta }\phi_{\tau ,k}$,

(75) $$\begin{array}{cc} \int_{R^{d}}{\left|D_{\tau ,k}\left(x\right)\right|}^{p_{\nu }}\;dx & =2^{mp_{\nu }\left(d/2+|\beta |\right)}\int_{R^{d}}{\left|\left(\partial^{\beta }\phi \right)\left(2^{m}x-k\right)\right|}^{p_{\nu }}\;dx\\ \; & =2^{m\{p_{\nu }\left(d/2+|\beta |)-d\right\}}\int_{R^{d}}{\left|\left(\partial^{\beta }\phi \right)\left(v\right)\right|}^{p_{\nu }}\;dv\\ \; & =2^{m\{p_{\nu }\left(d/2+|\beta |)-d\right\}}{∥\partial^{\beta }\phi ∥}_{p_{\nu }}^{p_{\nu }}, \end{array}$$

where the second equality follows from $v=2^{m}x-k$ and $dx=2^{-md}\;dv$. Combining (74) and (75),

(76) $$\begin{array}{cc} \; & {\left(E{|D_{\tau ,k}\left(X_{1}\right)|}^{p_{\nu }}\right)}^{(\nu -2)/\nu }\\ \; & \;\leq \;∥f_{X}{∥}_{\infty }^{(\nu -2)/\nu }{∥\partial^{\beta }\phi ∥}_{p_{\nu }}^{2}2^{m\{p_{\nu }(d/2+|\beta |)-d\}\left(\nu -2\right)/\nu }. \end{array}$$

Since

$$p_{\nu }\frac{\nu -2}{\nu }=2,$$

we have

$$\begin{array}{cc} \left\{p_{\nu }\left(\frac{d}{2}+\left|\beta \right|\right)-d\right\}\frac{\nu -2}{\nu } & =2\left(\frac{d}{2}+\left|\beta \right|\right)-d\frac{\nu -2}{\nu }\\ \; & =d+2|\beta |-d+\frac{2d}{\nu }\\ \; & =2|\beta |+\frac{2d}{\nu }. \end{array}$$

It follows from (76) that

(77) $${\left(E|D_{\tau ,k}\left(X_{1}\right){|}^{p_{\nu }}\right)}^{(\nu -2)/\nu }\leq C_{D}\;2^{2m(d/\nu +|\beta |)},$$

where

$$C_{D}=\;∥f_{X}{∥}_{\infty }^{(\nu -2)/\nu }{∥\partial^{\beta }\phi ∥}_{p_{\nu }}^{2}.$$

Substitution of (77) into (73) and then into (72) gives

(78) $$E\left[Z_{1,\tau ,k}^{2}\right]\leq C_{Z}\;2^{2m(d/\nu +|\beta |)},$$

with

$$C_{Z}=\frac{1}{\pi_{0}}{\left(E|\rho \left(Y_{1}\right){|}^{\nu }\right)}^{2/\nu }∥f_{X}{∥}_{\infty }^{(\nu -2)/\nu }{∥\partial^{\beta }\phi ∥}_{p_{\nu }}^{2}.$$

The constant $C_{Z}$ is finite and independent of *n*, *m*, and k. From (71) and (78),

(79) $$E\left[{\left({\tilde{a}}_{\tau ,k}^{\;\mathrm{ipw}}-{\bar{a}}_{\tau ,k}^{\;\mathrm{ipw}}\right)}^{2}\right]\leq C_{Z}\frac{2^{2m(d/\nu +|\beta |)}}{n}.$$

It remains to control the predictable term. Successively using (M.1), (N.2)(i), and the conditional density $f^{F_{i-1}}$, we have almost surely

(80) $$\begin{array}{cc} E[Z_{i,\tau ,k}|F_{i-1}] & =E\left[\rho \left(Y_{i}\right)D_{\tau ,k}\left(X_{i}\right)|F_{i-1}\right]\\ \; & =E\left[m_{\rho }\left(X_{i}\right)D_{\tau ,k}\left(X_{i}\right)|F_{i-1}\right]\\ \; & =\int_{R^{d}}m_{\rho }\left(x\right)D_{\tau ,k}\left(x\right)f^{F_{i-1}}\left(x\right)\;dx. \end{array}$$

On the other hand, by (68),

$$E\left[Z_{i,\tau ,k}\right]=\int_{R^{d}}m_{\rho }\left(x\right)D_{\tau ,k}\left(x\right)f_{X}\left(x\right)\;dx.$$

Consequently,

(81) $$\begin{array}{cc} {\bar{a}}_{\tau ,k}^{\;\mathrm{ipw}}-a_{\tau ,k} & =\frac{{\left(-1\right)}^{\left|\beta \right|}}{n}\sum_{i=1}^{n}\int_{R^{d}}m_{\rho }\left(x\right)D_{\tau ,k}\left(x\right)\left\{f^{F_{i-1}}\left(x\right)-f_{X}\left(x\right)\right\}\;dx. \end{array}$$

The quantitative stabilization condition (C.$1^{♯}$) directly gives

(82) $$E\left[{\left({\bar{a}}_{\tau ,k}^{\;\mathrm{ipw}}-a_{\tau ,k}\right)}^{2}\right]\leq C\frac{2^{2m(d/\nu +|\beta |)}}{n}.$$

Combining (69), (79), and (82), we obtain

$$E\left[{\left({\tilde{a}}_{\tau ,k}^{\;\mathrm{ipw}}-a_{\tau ,k}\right)}^{2}\right]\leq C^{\prime }\frac{2^{2m(d/\nu +|\beta |)}}{n},$$

where $C^{\prime }$ is independent of *n*, *m*, and k. This proves (65).

Finally, if ν≥4, then d/ν≤d/4. Since m≥0,

$$2^{2m(d/\nu +|\beta |)}\leq 2^{2m(d/4+|\beta |)}.$$

Therefore,

$$E\left[{\left({\tilde{a}}_{\tau ,k}^{\;\mathrm{ipw}}-a_{\tau ,k}\right)}^{2}\right]=O\left(\frac{2^{2m(d/4+|\beta |)}}{n}\right),$$

which is (66). □

#### 7.2.2. A Stronger Sufficient Condition for (C.6)

Suppose that there exists $C_{\mathrm{st}}>0$ such that

(83) $$E\left[\underset{x\in R^{d}}{\sup}{\left|\frac{1}{n}\sum_{i=1}^{n}f^{F_{i-1}}\left(x\right)-f_{X}\left(x\right)\right|}^{2}\right]\leq \frac{C_{\mathrm{st}}}{n},\;n\geq 1.$$

**Lemma** **9.** *Assume* (N.3)(i)*, the r-regularity of the multiresolution analysis with |β|≤r, and *(83). *Then, for every ν>2, every resolution level τ=m(n), and every $k\in Z^{d}$,*

(84) $$\begin{array}{cc} \; & E\left[{\left|\frac{1}{n}\sum_{i=1}^{n}\int_{R^{d}}m_{\rho }\left(x\right)\left(\partial^{\beta }\phi_{\tau ,k}\right)\left(x\right)\left\{f^{F_{i-1}}\left(x\right)-f_{X}\left(x\right)\right\}\;dx\right|}^{2}\right]\\ \; & \;\leq C\;\frac{2^{2m(|\beta |-d/2)}}{n}. \end{array}$$

*Consequently,*

(85) $$E\left[{\left|\frac{1}{n}\sum_{i=1}^{n}\int_{R^{d}}m_{\rho }\left(x\right)\left(\partial^{\beta }\phi_{\tau ,k}\right)\left(x\right)\left\{f^{F_{i-1}}\left(x\right)-f_{X}\left(x\right)\right\}\;dx\right|}^{2}\right]\leq C\;\frac{2^{2m(d/\nu +|\beta |)}}{n}.$$

*Thus, under* (N.3)(i)*, the stronger bound *(83) *implies the coefficient-level condition* (C.6).

**Proof.**  Put

$$\Delta_{n}\left(x\right):=\frac{1}{n}\sum_{i=1}^{n}f^{F_{i-1}}\left(x\right)-f_{X}\left(x\right),\;x\in R^{d}.$$

The random quantity appearing on the left-hand side of (84) can then be written as

$$R_{\tau ,k,n}:=\int_{R^{d}}m_{\rho }\left(x\right)\left(\partial^{\beta }\phi_{\tau ,k}\right)\left(x\right)\Delta_{n}\left(x\right)\;dx.$$

By the elementary inequality

$$\left|\int_{R^{d}}u\left(x\right)v\left(x\right)\;dx\right|\leq {∥u∥}_{L^{1}\left(R^{d}\right)}{∥v∥}_{L^{\infty }\left(R^{d}\right)}$$

applied with

$$u\left(x\right)=m_{\rho }\left(x\right)\left(\partial^{\beta }\phi_{\tau ,k}\right)\left(x\right),\;v\left(x\right)=\Delta_{n}\left(x\right),$$

we obtain

(86) $$\begin{array}{cc} |R_{\tau ,k,n}| & \leq \underset{x\in R^{d}}{\sup}|\Delta_{n}\left(x\right)|\int_{R^{d}}\left|m_{\rho }\left(x\right)\right|\left|\left(\partial^{\beta }\phi_{\tau ,k}\right)\left(x\right)\right|\;dx. \end{array}$$

Under (N.3)(i), there exists $C_{m}>0$ such that

$$\underset{x\in J}{\sup}\left|m_{\rho }\left(x\right)\right|\leq C_{m}.$$

Assuming that the support of the relevant wavelet term is contained in J, or equivalently that the preceding bound is valid on the region on which the coefficient is evaluated, it follows that

(87) $$\begin{array}{cc} \int_{R^{d}}\left|m_{\rho }\left(x\right)\right|\left|\left(\partial^{\beta }\phi_{\tau ,k}\right)\left(x\right)\right|\;dx & \leq C_{m}{∥\partial^{\beta }\phi_{\tau ,k}∥}_{L^{1}\left(R^{d}\right)}. \end{array}$$

By the definition of the dilated scaling functions,

$$\phi_{\tau ,k}\left(x\right)=2^{md/2}\phi (2^{m}x-k);$$

therefore,

$$\left(\partial^{\beta }\phi_{\tau ,k}\right)\left(x\right)=2^{m(d/2+|\beta |)}\left(\partial^{\beta }\phi \right)\left(2^{m}x-k\right).$$

Consequently,

(88) $$\begin{array}{cc} {∥\partial^{\beta }\phi_{\tau ,k}∥}_{L^{1}\left(R^{d}\right)} & =2^{m(d/2+|\beta |)}\int_{R^{d}}\left|\left(\partial^{\beta }\phi \right)\left(2^{m}x-k\right)\right|\;dx. \end{array}$$

Making the change of variables

$$v=2^{m}x-k,\;x=2^{-m}\left(v+k\right),\;dx=2^{-md}\;dv,$$

we obtain

(89) $$\begin{array}{cc} {∥\partial^{\beta }\phi_{\tau ,k}∥}_{L^{1}\left(R^{d}\right)} & =2^{m(d/2+|\beta |)}2^{-md}\int_{R^{d}}\left|\left(\partial^{\beta }\phi \right)\left(v\right)\right|\;dv\\ \; & =2^{m(|\beta |-d/2)}{∥\partial^{\beta }\phi ∥}_{L^{1}\left(R^{d}\right)}. \end{array}$$

The *r*-regularity and rapid decrease of the scaling function imply

$${∥\partial^{\beta }\phi ∥}_{L^{1}\left(R^{d}\right)}<\infty$$

for every |β|≤r. Combining (86), (87), and (89), we obtain the pathwise inequality

(90) $$\begin{array}{cc} |R_{\tau ,k,n}| & \leq C_{m}2^{m(|\beta |-d/2)}{∥\partial^{\beta }\phi ∥}_{1}\underset{x\in R^{d}}{\sup}\left|\Delta_{n}\left(x\right)\right|. \end{array}$$

After squaring,

(91) $$\begin{array}{cc} |R_{\tau ,k,n}{|}^{2} & \leq C_{m}^{2}2^{2m(|\beta |-d/2)}{∥\partial^{\beta }\phi ∥}_{1}^{2}\underset{x\in R^{d}}{\sup}{\left|\Delta_{n}\left(x\right)\right|}^{2}. \end{array}$$

Taking expectations and applying (C.$2^{⋆}$) gives

(92) $$\begin{array}{cc} E|R_{\tau ,k,n}{|}^{2} & \leq C_{m}^{2}2^{2m(|\beta |-d/2)}{∥\partial^{\beta }\phi ∥}_{1}^{2}E\left[\underset{x\in R^{d}}{\sup}{\left|\Delta_{n}\left(x\right)\right|}^{2}\right]\\ \; & \leq C_{m}^{2}C_{\mathrm{st}}{∥\partial^{\beta }\phi ∥}_{1}^{2}\frac{2^{2m(|\beta |-d/2)}}{n}. \end{array}$$

This proves (84).

It remains to compare the exponent in (84) with the exponent required in (C.$1^{♯}$). Since ν>2 and d≥1,

$$-\frac{d}{2}\leq \frac{d}{\nu }.$$

Therefore,

$$\left|\beta \right|-\frac{d}{2}\leq \left|\beta \right|+\frac{d}{\nu }.$$

Because m≥0,

$$2^{2m(|\beta |-d/2)}\leq 2^{2m(|\beta |+d/\nu )}.$$

It follows from (92) that

$$E|R_{\tau ,k,n}{|}^{2}\leq C\frac{2^{2m(|\beta |+d/\nu )}}{n},$$

which is precisely the estimate required in (C.6). □

#### 7.2.3. Conditions Used for Uniform Convergence

Throughout the proof of Theorem 2, assumptions (U.1)–(U.5) are understood in the form stated in Section 3. In particular, all references to (21)–(27) refer to the labels defined there.

### 7.3. Proof of Theorem 2

We first consider the IPW pseudo-estimator based on the true propensity score:

$${\tilde{\left(\partial^{\beta }r\right)}}_{n}^{\;\mathrm{ipw}}\left(\rho ;x\right)=\frac{{\left(-1\right)}^{\left|\beta \right|}}{nh_{n}^{d+|\beta |}}\sum_{i=1}^{n}\frac{\delta_{i}}{\pi (X_{i})}\rho \left(Y_{i}\right)K^{\left(\beta \right)}\left(\frac{x}{h_{n}},\frac{X_{i}}{h_{n}}\right).$$

Define its predictable counterpart by

$${\bar{r}}_{n}^{\;\mathrm{ipw}}\left(\rho ;x\right)=\frac{{\left(-1\right)}^{\left|\beta \right|}}{nh_{n}^{d+|\beta |}}\sum_{i=1}^{n}E\left[\left.\frac{\delta_{i}}{\pi (X_{i})}\rho \left(Y_{i}\right)K^{\left(\beta \right)}\left(\frac{x}{h_{n}},\frac{X_{i}}{h_{n}}\right)\right|F_{i-1}\right].$$

Then,

(93) $$\begin{array}{cc} \; & \underset{x\in D}{\sup}\left|{\tilde{\left(\partial^{\beta }r\right)}}_{n}^{\;\mathrm{ipw}}\left(\rho ;x\right)-E\left[{\tilde{\left(\partial^{\beta }r\right)}}_{n}^{\;\mathrm{ipw}}\left(\rho ;x\right)\right]\right|\\ \; & \;\leq G_{n,1}^{\mathrm{ipw}}\left(\rho \right)+G_{n,2}^{\mathrm{ipw}}\left(\rho \right), \end{array}$$

where

$$G_{n,1}^{\mathrm{ipw}}\left(\rho \right)=\underset{x\in D}{\sup}\left|{\tilde{\left(\partial^{\beta }r\right)}}_{n}^{\;\mathrm{ipw}}\left(\rho ;x\right)-{\bar{r}}_{n}^{\;\mathrm{ipw}}\left(\rho ;x\right)\right|$$

and

$$G_{n,2}^{\mathrm{ipw}}\left(\rho \right)=\underset{x\in D}{\sup}\left|{\bar{r}}_{n}^{\;\mathrm{ipw}}\left(\rho ;x\right)-E\left[{\tilde{\left(\partial^{\beta }r\right)}}_{n}^{\;\mathrm{ipw}}\left(\rho ;x\right)\right]\right|.$$

Let

$$\rho_{i,n}^{T}=\rho \left(Y_{i}\right)1_{\left\{|\rho \left(Y_{i}\right)|\leq T_{n}\right\}},\;\rho_{i,n}^{R}=\rho \left(Y_{i}\right)1_{\left\{|\rho \left(Y_{i}\right)|>T_{n}\right\}}.$$

Define

(94) $${\tilde{a}}_{\tau ,k}^{T,\mathrm{ipw}}=\frac{{\left(-1\right)}^{\left|\beta \right|}}{n}\sum_{i=1}^{n}\frac{\delta_{i}}{\pi (X_{i})}\rho_{i,n}^{T}\left(\partial^{\beta }\phi_{\tau ,k}\right)\left(X_{i}\right)$$

and

(95) $${\tilde{\left(\partial^{\beta }r\right)}}_{n}^{T,\mathrm{ipw}}\left(\rho ;x\right)=\sum_{k\in Z^{d}}{\tilde{a}}_{\tau ,k}^{T,\mathrm{ipw}}\phi_{\tau ,k}\left(x\right).$$

Equivalently,

(96) $${\tilde{\left(\partial^{\beta }r\right)}}_{n}^{T,\mathrm{ipw}}\left(\rho ;x\right)=\frac{{\left(-1\right)}^{\left|\beta \right|}}{nh_{n}^{d+|\beta |}}\sum_{i=1}^{n}\frac{\delta_{i}}{\pi (X_{i})}\rho_{i,n}^{T}K^{\left(\beta \right)}\left(\frac{x}{h_{n}},\frac{X_{i}}{h_{n}}\right).$$

Likewise, put

(97) $${\bar{r}}_{n}^{T,\mathrm{ipw}}\left(\rho ;x\right)=\frac{{\left(-1\right)}^{\left|\beta \right|}}{nh_{n}^{d+|\beta |}}\sum_{i=1}^{n}E\left[\left.\frac{\delta_{i}}{\pi (X_{i})}\rho_{i,n}^{T}K^{\left(\beta \right)}\left(\frac{x}{h_{n}},\frac{X_{i}}{h_{n}}\right)\right|F_{i-1}\right].$$

We have

$$\begin{array}{cc} G_{n,1}^{\mathrm{ipw}}\left(\rho \right) & \leq W_{n,1}^{\mathrm{ipw}}\left(\rho \right)+W_{n,2}^{\mathrm{ipw}}\left(\rho \right)+W_{n,3}^{\mathrm{ipw}}\left(\rho \right), \end{array}$$

where

$$\begin{array}{cc} W_{n,1}^{\mathrm{ipw}}\left(\rho \right) & =\underset{x\in D}{\sup}\left|{\tilde{\left(\partial^{\beta }r\right)}}_{n}^{\;\mathrm{ipw}}\left(\rho ;x\right)-{\tilde{\left(\partial^{\beta }r\right)}}_{n}^{T,\mathrm{ipw}}\left(\rho ;x\right)\right|,\\ W_{n,2}^{\mathrm{ipw}}\left(\rho \right) & =\underset{x\in D}{\sup}\left|{\tilde{\left(\partial^{\beta }r\right)}}_{n}^{T,\mathrm{ipw}}\left(\rho ;x\right)-{\bar{r}}_{n}^{T,\mathrm{ipw}}\left(\rho ;x\right)\right|,\\ W_{n,3}^{\mathrm{ipw}}\left(\rho \right) & =\underset{x\in D}{\sup}\left|{\bar{r}}_{n}^{T,\mathrm{ipw}}\left(\rho ;x\right)-{\bar{r}}_{n}^{\;\mathrm{ipw}}\left(\rho ;x\right)\right|. \end{array}$$

We first show that the truncation remainder is eventually zero. By the union bound, stationarity, Markov’s inequality, and (N.0),

$$\begin{array}{cc} P\left(\max_{1\leq i\leq n}\left|\rho \left(Y_{i}\right)\right|>T_{n}\right) & \leq \sum_{i=1}^{n}P\left(|\rho \left(Y_{i}\right)|>T_{n}\right)\\ \; & =n\;P\left(|\rho \left(Y_{1}\right)|>T_{n}\right)\\ \; & \leq nT_{n}^{-\nu }E{\left|\rho \left(Y_{1}\right)\right|}^{\nu }. \end{array}$$

Condition (26) and the Borel–Cantelli lemma imply

$$\max_{1\leq i\leq n}\left|\rho \left(Y_{i}\right)\right|\leq T_{n}\;a.s.$$

for all sufficiently large *n*. Consequently,

(98) $$W_{n,1}^{\mathrm{ipw}}\left(\rho \right)=0\;a.s.$$

eventually. The same event implies

(99) $$W_{n,3}^{\mathrm{ipw}}\left(\rho \right)=0\;a.s.$$

eventually. This avoids the invalid inference obtained from the weaker condition $\sum_{n}T_{n}^{-\nu }<\infty$, which controls only $\left|\rho (Y_{n})\right|$, not the maximum over 1≤i≤n. It remains to control $W_{n,2}^{\mathrm{ipw}}\left(\rho \right)$. Let

$$r_{n}={\left(\frac{\log n}{nh_{n}^{d+2|\beta |}}\right)}^{1/2}.$$

Cover *D* by $L_{n}$ closed cubes of side length $\ell_{n}$, with centers $x_{n,1},\ldots ,x_{n,L_{n}}$, where

$$\ell_{n}=c_{0}\frac{h_{n}^{d+|\beta |+1}r_{n}}{T_{n}},\;L_{n}\leq C_{D}\ell_{n}^{-d}.$$

Then,

$$\begin{array}{cc} W_{n,2}^{\mathrm{ipw}}\left(\rho \right) & \leq Q_{1}^{\mathrm{ipw}}+Q_{2}^{\mathrm{ipw}}+Q_{3}^{\mathrm{ipw}}, \end{array}$$

where

$$\begin{array}{cc} Q_{1}^{\mathrm{ipw}} & =\max_{1\leq j\leq L_{n}}\underset{x\in D\cap I_{n,j}}{\sup}\left|{\tilde{\left(\partial^{\beta }r\right)}}_{n}^{T,\mathrm{ipw}}\left(\rho ;x\right)-{\tilde{\left(\partial^{\beta }r\right)}}_{n}^{T,\mathrm{ipw}}\left(\rho ;x_{n,j}\right)\right|,\\ Q_{2}^{\mathrm{ipw}} & =\max_{1\leq j\leq L_{n}}\left|{\tilde{\left(\partial^{\beta }r\right)}}_{n}^{T,\mathrm{ipw}}\left(\rho ;x_{n,j}\right)-{\bar{r}}_{n}^{T,\mathrm{ipw}}\left(\rho ;x_{n,j}\right)\right|,\\ Q_{3}^{\mathrm{ipw}} & =\max_{1\leq j\leq L_{n}}\underset{x\in D\cap I_{n,j}}{\sup}\left|{\bar{r}}_{n}^{T,\mathrm{ipw}}\left(\rho ;x_{n,j}\right)-{\bar{r}}_{n}^{T,\mathrm{ipw}}\left(\rho ;x\right)\right|. \end{array}$$

By (22), $\pi \left(X_{i}\right)\geq \pi_{0}$, and $|\rho_{i,n}^{T}|\leq T_{n}$,

$$\begin{array}{cc} Q_{1}^{\mathrm{ipw}} & \leq \frac{CT_{n}\ell_{n}}{h_{n}^{d+|\beta |+1}}=O\left(r_{n}\right)\;a.s. \end{array}$$

The conditional-expectation contraction gives the same bound:

(100) $$Q_{3}^{\mathrm{ipw}}=O\left(r_{n}\right)\;a.s.$$

For $1\leq j\leq L_{n}$, define

(101) $$\begin{array}{cc} Z_{n,i,j} & ={\left(-1\right)}^{\left|\beta \right|}[\frac{\delta_{i}}{\pi (X_{i})}\rho_{i,n}^{T}K^{\left(\beta \right)}\left(\frac{x_{n,j}}{h_{n}},\frac{X_{i}}{h_{n}}\right)\\ \; & \;\;-E\left[\left.\frac{\delta_{i}}{\pi (X_{i})}\rho_{i,n}^{T}K^{\left(\beta \right)}\left(\frac{x_{n,j}}{h_{n}},\frac{X_{i}}{h_{n}}\right)\right|F_{i-1}\right]]. \end{array}$$

For fixed n,j, ${\left\{Z_{n,i,j},F_{i}\right\}}_{1\leq i\leq n}$ is a martingale difference array and

$$Q_{2}^{\mathrm{ipw}}=\max_{1\leq j\leq L_{n}}\left|\frac{1}{nh_{n}^{d+|\beta |}}\sum_{i=1}^{n}Z_{n,i,j}\right|.$$

The truncation and positivity imply the deterministic increment bound

(102) $$|Z_{n,i,j}|\leq \frac{2C_{K}T_{n}}{\pi_{0}}=:B_{n}.$$

Furthermore, by conditional Jensen’s inequality,

$$E\left[Z_{n,i,j}^{2}|F_{i-1}\right]\leq E\left[\left.{\left(\frac{\delta_{i}}{\pi (X_{i})}\rho_{i,n}^{T}K^{\left(\beta \right)}\left(\frac{x_{n,j}}{h_{n}},\frac{X_{i}}{h_{n}}\right)\right)}^{2}\right|F_{i-1}\right].$$

Since $\delta_{i}^{2}=\delta_{i}$, MAR and positivity yield

$$\begin{array}{cc} \; & E\left[\left.{\left(\frac{\delta_{i}}{\pi (X_{i})}\rho_{i,n}^{T}K^{\left(\beta \right)}\left(\frac{x_{n,j}}{h_{n}},\frac{X_{i}}{h_{n}}\right)\right)}^{2}\right|F_{i-1}\right]\\ \; & \;\leq \frac{1}{\pi_{0}}E\left[\left.|\rho \left(Y_{i}\right){|}^{2}{\left|K^{\left(\beta \right)}\left(\frac{x_{n,j}}{h_{n}},\frac{X_{i}}{h_{n}}\right)\right|}^{2}\right|F_{i-1}\right]. \end{array}$$

By (U.2), conditional Hölder’s inequality, (U.3), and the kernel integrability bound,

$$\begin{array}{cc} E[Z_{n,i,j}^{2}|F_{i-1}] & \leq C\int_{R^{d}}{\left|K^{\left(\beta \right)}\left(\frac{x_{n,j}}{h_{n}},\frac{y}{h_{n}}\right)\right|}^{2}f^{F_{i-1}}\left(y\right)\;d\;y\\ \; & \leq Ch_{n}^{d}\int_{R^{d}}{\left|K^{\left(\beta \right)}\left(0,u\right)\right|}^{2}\;d\;u\\ \; & \leq Ch_{n}^{d}\;a.s. \end{array}$$

Hence, the predictable quadratic variation satisfies

$$V_{n,j}:=\sum_{i=1}^{n}E\left[Z_{n,i,j}^{2}|F_{i-1}\right]\leq Cnh_{n}^{d}\;a.s.$$

Then, for every t>0, Freedman’s inequality gives

$$P\left(\left|\sum_{i=1}^{n}Z_{n,i,j}\right|>t\right)\leq 2\exp\left\{-\frac{t^{2}}{2(Cnh_{n}^{d}+B_{n}t/3)}\right\}.$$

Choose

$$t_{n}=Anh_{n}^{d+|\beta |}r_{n}=A\sqrt{nh_{n}^{d}\log n}.$$

By (25),

$$\frac{B_{n}t_{n}}{nh_{n}^{d}}\leq CT_{n}{\left(\frac{\log n}{nh_{n}^{d}}\right)}^{1/2}⟶0.$$

Thus, for all sufficiently large *n*,

$$P\left(\left|\sum_{i=1}^{n}Z_{n,i,j}\right|>t_{n}\right)\leq 2n^{-cA^{2}}$$

for some c>0. By the union bound,

$$P\left(Q_{2}^{\mathrm{ipw}}>Ar_{n}\right)\leq 2L_{n}n^{-cA^{2}}.$$

The definition of $\ell_{n}$ together with (24)–(25) implies that $L_{n}$ grows at most polynomially. Choosing sufficiently large *A* makes $\sum_{n}L_{n}n^{-cA^{2}}<\infty$. Borel–Cantelli then yields

(103) $$Q_{2}^{\mathrm{ipw}}=O\left(r_{n}\right)\;a.s.$$

Combining (98)–(100), and (103), we obtain

(104) $$G_{n,1}^{\mathrm{ipw}}\left(\rho \right)=O\left[{\left(\frac{\log n}{nh_{n}^{d+2|\beta |}}\right)}^{1/2}\right]\;a.s.$$

We next control the predictable component. By the MAR identity and (N.2)(i),

$$E\left[\left.\frac{\delta_{i}}{\pi (X_{i})}\rho \left(Y_{i}\right)\right|G_{i-1}\right]=m_{\rho }\left(X_{i}\right)\;a.s.$$

Consequently,

(105) $$\begin{array}{cc} G_{n,2}^{\mathrm{ipw}}\left(\rho \right) & =\underset{x\in D}{\sup}\left|\frac{{\left(-1\right)}^{\left|\beta \right|}}{h_{n}^{d+|\beta |}}\int_{R^{d}}m_{\rho }\left(y\right)K^{\left(\beta \right)}\left(\frac{x}{h_{n}},\frac{y}{h_{n}}\right)\Delta_{n}\left(y\right)\;dy\right|. \end{array}$$

Using (N.3)(i), the predictable bound used to verify (41), and the $L^{1}$-kernel bound, we find

$$\begin{array}{cc} G_{n,2}^{\mathrm{ipw}}\left(\rho \right) & \leq \frac{C_{m}{∥\Delta_{n}∥}_{\infty }}{h_{n}^{d+|\beta |}}\underset{x\in D}{\sup}\int_{R^{d}}\left|K^{\left(\beta \right)}\left(\frac{x}{h_{n}},\frac{y}{h_{n}}\right)\right|\;d\;y\\ \; & =\frac{C_{m}{∥\Delta_{n}∥}_{\infty }}{h_{n}^{\left|\beta \right|}}\underset{u\in R^{d}}{\sup}\int_{R^{d}}\left|K^{\left(\beta \right)}\left(u,v\right)\right|\;d\;v\\ \; & =O\left(b_{n}h_{n}^{-|\beta |}\right)\;a.s. \end{array}$$

Therefore,

(106) $$\begin{array}{cc} \; & \underset{x\in D}{\sup}\left|{\tilde{\left(\partial^{\beta }r\right)}}_{n}^{\;\mathrm{ipw}}\left(\rho ;x\right)-E\left[{\tilde{\left(\partial^{\beta }r\right)}}_{n}^{\;\mathrm{ipw}}\left(\rho ;x\right)\right]\right|\\ \; & \;=O\left[{\left(\frac{\log n}{nh_{n}^{d+2|\beta |}}\right)}^{1/2}\right]+O\left(b_{n}h_{n}^{-|\beta |}\right)\;a.s. \end{array}$$

We finally pass to the feasible estimator. We have

(107) $$\begin{array}{cc} \; & {\hat{\left(\partial^{\beta }r\right)}}_{n}^{\;\mathrm{mar}}\left(\rho ;x\right)-{\tilde{\left(\partial^{\beta }r\right)}}_{n}^{\;\mathrm{ipw}}\left(\rho ;x\right)\\ \; & \;=\frac{{\left(-1\right)}^{\left|\beta \right|}}{nh_{n}^{d+|\beta |}}\sum_{i=1}^{n}\delta_{i}\rho \left(Y_{i}\right)K^{\left(\beta \right)}\left(\frac{x}{h_{n}},\frac{X_{i}}{h_{n}}\right)\left[\frac{1}{{\hat{\pi }}_{n}\left(X_{i}\right)}-\frac{1}{\pi (X_{i})}\right]. \end{array}$$

By (M.2) and (M.4), for all sufficiently large *n*,

$$\underset{x\in J}{\sup}\left|\frac{1}{{\hat{\pi }}_{n}\left(x\right)}-\frac{1}{\pi (x)}\right|\leq \frac{2}{\pi_{0}^{2}}{∥{\hat{\pi }}_{n}-\pi ∥}_{\infty ,J}=O\left(a_{n}\right)\;a.s.$$

Therefore,

(108) $$\begin{array}{cc} \; & \underset{x\in D}{\sup}\left|{\hat{\left(\partial^{\beta }r\right)}}_{n}^{\;\mathrm{mar}}\left(\rho ;x\right)-{\tilde{\left(\partial^{\beta }r\right)}}_{n}^{\;\mathrm{ipw}}\left(\rho ;x\right)\right|\\ \; & \;\leq Ca_{n}\underset{x\in D}{\sup}\frac{1}{nh_{n}^{d+|\beta |}}\sum_{i=1}^{n}\delta_{i}\left|\rho \left(Y_{i}\right)\right|\left|K^{\left(\beta \right)}\left(\frac{x}{h_{n}},\frac{X_{i}}{h_{n}}\right)\right|. \end{array}$$

Under the same conditional moment, density, and kernel envelope bounds used above, the last supremum is $O(h_{n}^{-|\beta |})$ almost surely. Hence,

$$\underset{x\in D}{\sup}\left|{\hat{\left(\partial^{\beta }r\right)}}_{n}^{\;\mathrm{mar}}\left(\rho ;x\right)-{\tilde{\left(\partial^{\beta }r\right)}}_{n}^{\;\mathrm{ipw}}\left(\rho ;x\right)\right|=O\left(a_{n}h_{n}^{-|\beta |}\right)\;a.s.$$

Combining this estimate with (106) gives

$$\begin{array}{cc} \; & \underset{x\in D}{\sup}\left|{\hat{\left(\partial^{\beta }r\right)}}_{n}^{\;\mathrm{mar}}\left(\rho ;x\right)-E\left[{\tilde{\left(\partial^{\beta }r\right)}}_{n}^{\;\mathrm{ipw}}\left(\rho ;x\right)\right]\right|\\ \; & \;=O\left[{\left(\frac{\log n}{nh_{n}^{d+2|\beta |}}\right)}^{1/2}\right]+O\left(b_{n}h_{n}^{-|\beta |}\right)+O\left(a_{n}h_{n}^{-|\beta |}\right)\;a.s. \end{array}$$

The propensity estimation term is asymptotically negligible relative to the oracle stochastic and predictable terms. Adding the deterministic projection bias from Lemma 3 completes the proof of Theorem 2. □

#### Conditions Used for Pointwise Asymptotic Normality

Throughout the proof of Theorem 3, assumptions (CLT.1)–(CLT.8) are understood in the form stated in Section 3. All references to (28)–(36) refer to the labels defined there.

### 7.4. Proof of Theorem 3

By (CLT.1), the dyadic phase $\vartheta_{n}$ converges to $\vartheta \in {\left[0,1\right)}^{d}$. Therefore, the proof identifies the phase-dependent variance in (43). If the kernel energy is phase-invariant, then the same variance applies along the full sequence; otherwise, the conclusion is understood along sequences satisfying (28), exactly as stated in Theorem 3. For notational convenience, we write

$$K_{n,i}\left(x\right):=K^{\left(\beta \right)}\left(\frac{x}{h_{n}},\frac{X_{i}}{h_{n}}\right)$$

and

$$g_{\beta }\left(x\right):=\left(\partial^{\beta }r\right)\left(\rho ;x\right).$$

Recall that

$${\tilde{\left(\partial^{\beta }r\right)}}_{n}^{\;\mathrm{ipw}}\left(\rho ;x\right)=\frac{{\left(-1\right)}^{\left|\beta \right|}}{nh_{n}^{d+|\beta |}}\sum_{i=1}^{n}\frac{\delta_{i}}{\pi (X_{i})}\rho \left(Y_{i}\right)K_{n,i}\left(x\right)$$

and

$${\bar{r}}_{n}^{\;\mathrm{ipw}}\left(\rho ;x\right)=\frac{{\left(-1\right)}^{\left|\beta \right|}}{nh_{n}^{d+|\beta |}}\sum_{i=1}^{n}E\left[\left.\frac{\delta_{i}}{\pi (X_{i})}\rho \left(Y_{i}\right)K_{n,i}\left(x\right)\right|F_{i-1}\right].$$

We use the exact decomposition

(109) $$\begin{array}{cc} \; & \sqrt{nh_{n}^{d+2|\beta |}}\left[{\hat{\left(\partial^{\beta }r\right)}}_{n}^{\;\mathrm{mar}}\left(\rho ;x\right)-g_{\beta }\left(x\right)\right]\\ \; & \;=S_{n}\left(x\right)+R_{n,1}\left(x\right)+R_{n,2}\left(x\right), \end{array}$$

where

$$\begin{array}{cc} S_{n}\left(x\right) & =\sqrt{nh_{n}^{d+2|\beta |}}\left[{\tilde{\left(\partial^{\beta }r\right)}}_{n}^{\;\mathrm{ipw}}\left(\rho ;x\right)-{\bar{r}}_{n}^{\;\mathrm{ipw}}\left(\rho ;x\right)\right],\\ R_{n,1}\left(x\right) & =\sqrt{nh_{n}^{d+2|\beta |}}\left[{\bar{r}}_{n}^{\;\mathrm{ipw}}\left(\rho ;x\right)-g_{\beta }\left(x\right)\right],\\ R_{n,2}\left(x\right) & =\sqrt{nh_{n}^{d+2|\beta |}}\left[{\hat{\left(\partial^{\beta }r\right)}}_{n}^{\;\mathrm{mar}}\left(\rho ;x\right)-{\tilde{\left(\partial^{\beta }r\right)}}_{n}^{\;\mathrm{ipw}}\left(\rho ;x\right)\right]. \end{array}$$

We first identify the deterministic and predictable components of $R_{n,1}\left(x\right)$. By adding and subtracting the unconditional expectation,

$$\begin{array}{c} {\bar{r}}_{n}^{\;\mathrm{ipw}}\left(\rho ;x\right)-g_{\beta }\left(x\right)=B_{n,1}^{\mathrm{ipw}}\left(x\right)+B_{n,2}^{\mathrm{ipw}}\left(x\right), \end{array}$$

where

$$B_{n,1}^{\mathrm{ipw}}\left(x\right)={\bar{r}}_{n}^{\;\mathrm{ipw}}\left(\rho ;x\right)-E\left[{\tilde{\left(\partial^{\beta }r\right)}}_{n}^{\;\mathrm{ipw}}\left(\rho ;x\right)\right]$$

and

$$B_{n,2}^{\mathrm{ipw}}\left(x\right)=E\left[{\tilde{\left(\partial^{\beta }r\right)}}_{n}^{\;\mathrm{ipw}}\left(\rho ;x\right)\right]-g_{\beta }\left(x\right).$$

By (M.1) and (N.2)(i),

$$E\left[\left.\frac{\delta_{i}}{\pi (X_{i})}\rho \left(Y_{i}\right)\right|G_{i-1}\right]=m_{\rho }\left(X_{i}\right)\;a.s.$$

Since the conditional law of $X_{i}$ given $F_{i-1}$ has density $f^{F_{i-1}}$, it follows that

(110) $$\begin{array}{cc} B_{n,1}^{\mathrm{ipw}}\left(x\right)\; & =\frac{{\left(-1\right)}^{\left|\beta \right|}}{h_{n}^{d+|\beta |}}\int_{R^{d}}m_{\rho }\left(u\right)K^{\left(\beta \right)}\left(\frac{x}{h_{n}},\frac{u}{h_{n}}\right)\\ \; & \;\;\times \left[\frac{1}{n}\sum_{i=1}^{n}f^{F_{i-1}}\left(u\right)-f_{X}\left(u\right)\right]\;du. \end{array}$$

Therefore, Condition (34) gives

(111) $$\sqrt{nh_{n}^{d+2|\beta |}}\;B_{n,1}^{\mathrm{ipw}}\left(x\right)=o_{P}\left(1\right).$$

Notice that qualitative convergence in (C.2) alone does not imply this negligibility of the central limit scale. By the exact IPW compensation identity,

(112) $$\begin{array}{cc} E\left[{\tilde{\left(\partial^{\beta }r\right)}}_{n}^{\;\mathrm{ipw}}\left(\rho ;x\right)\right] & =\frac{{\left(-1\right)}^{\left|\beta \right|}}{h_{n}^{d+|\beta |}}\int_{R^{d}}r\left(\rho ;u\right)K^{\left(\beta \right)}\left(\frac{x}{h_{n}},\frac{u}{h_{n}}\right)\;du\\ \; & =P_{V_{m(n)}}g_{\beta }\left(x\right). \end{array}$$

The second equality follows from the projection identity and integration by parts. The latter is justified by the weak differentiability of r(ρ;·), the compact support assumptions in (C.5), and the regularity of the scaling function; compact support of the scaling function alone would not justify moving derivatives from the target. Hence,

$$B_{n,2}^{\mathrm{ipw}}\left(x\right)=P_{V_{m(n)}}g_{\beta }\left(x\right)-g_{\beta }\left(x\right),$$

and (35) gives

(113) $$\sqrt{nh_{n}^{d+2|\beta |}}\;B_{n,2}^{\mathrm{ipw}}\left(x\right)=o\left(1\right).$$

Combining (111) and (113), we obtain

(114) $$R_{n,1}\left(x\right)=o_{P}\left(1\right).$$

We next prove a martingale central limit theorem for $S_{n}\left(x\right)$. Set

$$\xi_{ni}^{\mathrm{ipw}}\left(x\right)=\frac{{\left(-1\right)}^{\left|\beta \right|}}{\sqrt{nh_{n}^{d}}}\frac{\delta_{i}}{\pi (X_{i})}\rho \left(Y_{i}\right)K_{n,i}\left(x\right)$$

and

$$\chi_{ni}^{\mathrm{ipw}}\left(x\right)=\xi_{ni}^{\mathrm{ipw}}\left(x\right)-E\left[\left.\xi_{ni}^{\mathrm{ipw}}\left(x\right)\right|F_{i-1}\right].$$

Then,

$$S_{n}\left(x\right)=\sum_{i=1}^{n}\chi_{ni}^{\mathrm{ipw}}\left(x\right)$$

and ${\left\{\chi_{ni}^{\mathrm{ipw}}\left(x\right),F_{i}\right\}}_{1\leq i\leq n}$ is a martingale difference triangular array. The correct candidate variance is phase-dependent:

(115) $$\Sigma_{\mathrm{mar},(\beta )}^{2}\left(x;\vartheta \right)=\frac{m_{\rho ,2}\left(x\right)f_{X}\left(x\right)}{\pi (x)}\int_{R^{d}}{\left[K^{\left(\beta \right)}\left(\vartheta ,\vartheta +v\right)\right]}^{2}\;dv.$$

We verify the conditional variance convergence

(116) $$\sum_{i=1}^{n}E\left[\left.{\left(\chi_{ni}^{\mathrm{ipw}}\left(x\right)\right)}^{2}\right|F_{i-1}\right]\overset{P}{\to }\Sigma_{\mathrm{mar},(\beta )}^{2}\left(x;\vartheta \right).$$

Because conditional expectation is an $L^{2}$-contraction,

$$E\left[{\left(\chi_{ni}^{\mathrm{ipw}}\right)}^{2}|F_{i-1}\right]=E\left[{\left(\xi_{ni}^{\mathrm{ipw}}\right)}^{2}|F_{i-1}\right]-{\left(E[\xi_{ni}^{\mathrm{ipw}}|F_{i-1}]\right)}^{2}.$$

The predictable quadratic variation hypothesis in Theorem 3 requires the squared predictable means to be negligible. Under the local sufficient conditions above, the same localization calculation used for (34) gives

(117) $$\sum_{i=1}^{n}{\left(E[\xi_{ni}^{\mathrm{ipw}}\left(x\right)|F_{i-1}]\right)}^{2}=o_{P}\left(1\right).$$

This is a separate quadratic estimate, and does not follow merely by squaring the aggregate centering bound. Thus, it remains to calculate the first conditional second-moment term. Since $\delta_{i}^{2}=\delta_{i}$, (M.1) and (N.2)(ii) give

(118) $$E\left[\left.\frac{\delta_{i}^{2}}{\pi {\left(X_{i}\right)}^{2}}\rho {\left(Y_{i}\right)}^{2}\right|G_{i-1}\right]=\frac{m_{\rho ,2}\left(X_{i}\right)}{\pi (X_{i})}\;a.s.$$

Consequently,

(119) $$\begin{array}{cc} \; & \sum_{i=1}^{n}E\left[\left.{\left(\xi_{ni}^{\mathrm{ipw}}\left(x\right)\right)}^{2}\right|F_{i-1}\right]\\ \; & \;=\frac{1}{nh_{n}^{d}}\sum_{i=1}^{n}\int_{R^{d}}\frac{m_{\rho ,2}\left(u\right)}{\pi (u)}{\left[K^{\left(\beta \right)}\left(\frac{x}{h_{n}},\frac{u}{h_{n}}\right)\right]}^{2}f^{F_{i-1}}\left(u\right)\;du. \end{array}$$

Set

$$k_{n}=\left\lfloor \frac{x}{h_{n}}\right\rfloor \in Z^{d},\;\vartheta_{n}=\frac{x}{h_{n}}-k_{n}.$$

With the change of variables $u=x+h_{n}v$ and using the integer-shift periodicity

$$K^{\left(\beta \right)}\left(a+k,b+k\right)=K^{\left(\beta \right)}\left(a,b\right),\;k\in Z^{d},$$

we obtain

$$\begin{array}{cc} \; & \sum_{i=1}^{n}E\left[\left.{\left(\xi_{ni}^{\mathrm{ipw}}\left(x\right)\right)}^{2}\right|F_{i-1}\right] \end{array}$$

(120) $$\begin{array}{cc} \; & \;=\int_{R^{d}}\frac{m_{\rho ,2}\left(x+h_{n}v\right)}{\pi (x+h_{n}v)}{\left[K^{\left(\beta \right)}\left(\vartheta_{n},\vartheta_{n}+v\right)\right]}^{2} \end{array}$$

(121) $$\begin{array}{cc} \; & \;\;\times \left[\frac{1}{n}\sum_{i=1}^{n}f^{F_{i-1}}\left(x+h_{n}v\right)\right]\;dv. \end{array}$$

For each fixed v, conditions (28) and (29) together with continuity at x imply convergence in probability of the integrand to

$$\frac{m_{\rho ,2}\left(x\right)f_{X}\left(x\right)}{\pi (x)}{\left[K^{\left(\beta \right)}\left(\vartheta ,\vartheta +v\right)\right]}^{2}.$$

The envelope assumptions (30) and (31) imply uniform integrability of the integrands. Truncating first to {∥v∥≤M}, applying dominated convergence there, and then letting M→∞ yields

(122) $$\sum_{i=1}^{n}E\left[\left.{\left(\xi_{ni}^{\mathrm{ipw}}\left(x\right)\right)}^{2}\right|F_{i-1}\right]\overset{P}{\to }\Sigma_{\mathrm{mar},(\beta )}^{2}\left(x;\vartheta \right).$$

Together with (117), this proves (116). We now verify the following conditional Lindeberg condition: for every ε>0,

(123) $$\sum_{i=1}^{n}E\left[\left.{\left(\chi_{ni}^{\mathrm{ipw}}\left(x\right)\right)}^{2}1_{\left\{|\chi_{ni}^{\mathrm{ipw}}\left(x\right)|>\varepsilon \right\}}\right|F_{i-1}\right]\overset{P}{\to }0.$$

Choose η∈(0,ν−2). The elementary inequality

$${\left|u-v\right|}^{2+\eta }\leq 2^{1+\eta }\left({\left|u\right|}^{2+\eta }+{\left|v\right|}^{2+\eta }\right)$$

and conditional Jensen’s inequality imply

$$E\left[|\chi_{ni}^{\mathrm{ipw}}{|}^{2+\eta }|F_{i-1}\right]\leq 2^{2+\eta }E\left[|\xi_{ni}^{\mathrm{ipw}}{|}^{2+\eta }|F_{i-1}\right].$$

Hence, conditional Markov’s inequality gives

(124) $$\begin{array}{cc} \; & \sum_{i=1}^{n}E\left[\left.{\left(\chi_{ni}^{\mathrm{ipw}}\right)}^{2}1_{\left\{|\chi_{ni}^{\mathrm{ipw}}|>\varepsilon \right\}}\right|F_{i-1}\right]\\ \; & \;\leq C_{\varepsilon }\sum_{i=1}^{n}E\left[\left.|\xi_{ni}^{\mathrm{ipw}}{|}^{2+\eta }\right|F_{i-1}\right]. \end{array}$$

Taking expectations and using positivity, Hölder’s inequality with the marginal ν-moment, boundedness of $f_{X}$, and (32), we obtain

(125) $$\begin{array}{cc} \sum_{i=1}^{n}E\left[|\xi_{ni}^{\mathrm{ipw}}{\left(x\right)|}^{2+\eta }\right] & \leq Cn{\left(nh_{n}^{d}\right)}^{-(2+\eta )/2}h_{n}^{d}\\ \; & =C{\left(nh_{n}^{d}\right)}^{-\eta /2}⟶0 \end{array}$$

by (33). Therefore, the expectation of the left-hand side of (124) tends to zero. Since that left-hand side is non-negative, Markov’s inequality proves (123). The martingale central limit theorem now yields

(126) $$S_{n}\left(x\right)\overset{D}{\to }N\left(0,\Sigma_{\mathrm{mar},(\beta )}^{2}\left(x;\vartheta \right)\right).$$

Combining (126) with (114), we obtain

(127) $$\sqrt{nh_{n}^{d+2|\beta |}}\left[{\tilde{\left(\partial^{\beta }r\right)}}_{n}^{\;\mathrm{ipw}}\left(\rho ;x\right)-g_{\beta }\left(x\right)\right]\overset{D}{\to }N\left(0,\Sigma_{\mathrm{mar},(\beta )}^{2}\left(x;\vartheta \right)\right).$$

It remains to prove that replacing π by ${\hat{\pi }}_{n}$ is negligible. By direct subtraction,

$$\begin{array}{cc} R_{n,2}\left(x\right) & =\frac{{\left(-1\right)}^{\left|\beta \right|}}{\sqrt{nh_{n}^{d}}}\sum_{i=1}^{n}\delta_{i}\rho \left(Y_{i}\right)K_{n,i}\left(x\right)\left[\frac{1}{{\hat{\pi }}_{n}\left(X_{i}\right)}-\frac{1}{\pi (X_{i})}\right]. \end{array}$$

By positivity and uniform consistency, for all sufficiently large *n*,

$$\underset{u\in J}{\sup}\left|\frac{1}{{\hat{\pi }}_{n}\left(u\right)}-\frac{1}{\pi (u)}\right|\leq \frac{2}{\pi_{0}^{2}}{∥{\hat{\pi }}_{n}-\pi ∥}_{\infty ,J}.$$

Therefore,

(128) $$\begin{array}{cc} |R_{n,2}\left(x\right)| & \leq C\sqrt{nh_{n}^{d}}\;{∥{\hat{\pi }}_{n}-\pi ∥}_{\infty ,J}\\ \; & \;\times \frac{1}{nh_{n}^{d}}\sum_{i=1}^{n}\delta_{i}|\rho \left(Y_{i}\right)\left|\right|K_{n,i}\left(x\right)|. \end{array}$$

The last average is $O_{P}\left(1\right)$. To see this, truncate |ρ| at a deterministic level, apply the localized kernel $L^{1}$ bound to the truncated part, and control the tail by the ν-moment in (N.0) and positivity. The predictable contribution is bounded by the local conditional density envelope, while the centered contribution is handled by the same martingale localization inequality used in the uniform argument. This establishes tightness of the displayed average; a bounded expectation alone would not control its conditional fluctuation. Consequently,

(129) $$R_{n,2}\left(x\right)=O_{P}\left(\sqrt{nh_{n}^{d}}\;{∥{\hat{\pi }}_{n}-\pi ∥}_{\infty ,J}\right)=o_{P}\left(1\right)$$

by (36). Notice that no additional factor $h_{n}^{-|\beta |}$ appears here, as the derivative normalization has already canceled in the definition of $R_{n,2}$. Finally, (109), (114), (126), and (129) together with Slutsky’s theorem give

$$\sqrt{nh_{n}^{d+2|\beta |}}\left[{\hat{\left(\partial^{\beta }r\right)}}_{n}^{\;\mathrm{mar}}\left(\rho ;x\right)-g_{\beta }\left(x\right)\right]\overset{D}{\to }N\left(0,\Sigma_{\mathrm{mar},(\beta )}^{2}\left(x;\vartheta \right)\right).$$

The variance

$$\frac{m_{\rho ,2}\left(x\right)f_{X}\left(x\right)}{\pi (x)}\int_{R^{d}}{\left[K^{\left(\beta \right)}\left(0,v\right)\right]}^{2}\;dv$$

is recovered if either

$$\vartheta_{n}⟶0$$

or the integral

$$\int_{R^{d}}{\left[K^{\left(\beta \right)}\left(\theta ,\theta +v\right)\right]}^{2}\;dv$$

is independent of $\theta \in {\left[0,1\right)}^{d}$.

Under the phase qualification stated at the beginning of the proof, this completes the proof of Theorem 3. In the absence of that qualification, the same argument proves the corresponding subsequential central limit theorem with variance $\Sigma_{\mathrm{mar},(\beta )}^{2}\left(x;\vartheta \right)$. □

## 8. Besov Spaces

This appendix records the Besov space facts used in the approximation arguments of the paper. Its purpose is not to develop the general theory of Besov spaces but to specify a mathematically unambiguous regularity scale and identify the precise consequences invoked in the wavelet analysis. Besov spaces are particularly natural in this setting because their norms admit equivalent descriptions by both finite differences and wavelet coefficients; the first description makes the underlying smoothness requirement intrinsic, whereas the second converts that regularity into quantitative decay of multiresolution coefficients, and hence into projection bias bounds. Following [50], let 1≤p,q≤∞. For a vector $\tau \in R^{d}$, define the translation operator

$$\left(S_{\tau }f\right)\left(x\right)=f\left(x-\tau \right),\;x\in R^{d}.$$

For 0<s<1, the first-order modulus of smoothness is

$$\omega_{1}{\left(f,t\right)}_{p}=\underset{∥\tau ∥\leq t}{\sup}{∥S_{\tau }f-f∥}_{L_{p}},\;t>0,$$

and the corresponding Besov seminorm may be written equivalently as

$$\ell_{s,p,q}\left(f\right)={\left(\int_{R^{d}}{\left[{∥\tau ∥}^{-s}{∥S_{\tau }f-f∥}_{L_{p}}\right]}^{q}\frac{d\tau }{{∥\tau ∥}^{d}}\right)}^{1/q},$$

with the usual modification when q=∞:

$$\ell_{s,p,\infty }\left(f\right)=\underset{\tau \neq 0}{\sup}\frac{{∥S_{\tau }f-f∥}_{L_{p}}}{{∥\tau ∥}^{s}}.$$

The Besov space is then

$$B_{s,p,q}\left(R^{d}\right)=\left\{f\in L_{p}\left(R^{d}\right):\ell_{s,p,q}\left(f\right)<\infty \right\},$$

equipped with the norm

$${∥f∥}_{B_{s,p,q}}={∥f∥}_{L_{p}}+\ell_{s,p,q}\left(f\right).$$

The integral over all $\tau \in R^{d}$ may equivalently be restricted to ∥τ∥≤1, since the contribution of large translations is controlled by ${∥f∥}_{L_{p}}$. At the borderline value s=1, first-order differences do not provide the appropriate Zygmund regularity scale. Therefore, one uses the second-order symmetric difference

$$\Delta_{\tau }^{2}f=S_{\tau }f+S_{-\tau }f-2f$$

and defines

$$\ell_{1,p,q}\left(f\right)={\left(\int_{R^{d}}{\left[{∥\tau ∥}^{-1}{∥S_{\tau }f+S_{-\tau }f-2f∥}_{L_{p}}\right]}^{q}\frac{d\tau }{{∥\tau ∥}^{d}}\right)}^{1/q},$$

with

$$\ell_{1,p,\infty }\left(f\right)=\underset{\tau \neq 0}{\sup}\frac{{∥S_{\tau }f+S_{-\tau }f-2f∥}_{L_{p}}}{∥\tau ∥}.$$

For arbitrary s>0, the cleanest nonrecursive definition uses differences of an integer order k>s. Let

$$\Delta_{\tau }^{k}={\left(S_{\tau }-I\right)}^{k}$$

and

$$\omega_{k}{\left(f,t\right)}_{p}=\underset{∥\tau ∥\leq t}{\sup}{∥\Delta_{\tau }^{k}f∥}_{L_{p}}.$$

Then, $f\in B_{s,p,q}\left(R^{d}\right)$ if and only if

$${∥f∥}_{L_{p}}+{\left(\int_{0}^{1}{\left[t^{-s}\omega_{k}{\left(f,t\right)}_{p}\right]}^{q}\frac{dt}{t}\right)}^{1/q}<\infty ,$$

with the usual supremum interpretation when q=∞. Different integers k>s yield equivalent norms. This formulation avoids the ambiguity inherent in writing $s={\left[s\right]}^{-}+{\left\{s\right\}}^{+}$ at integer values of *s* and does not require fractional regularity to be imposed on every derivative of order below [s]. An equivalent derivative characterization is available when s=m+σ, where $m\in N_{0}$ and 0<σ<1: under the standard distributional interpretation,

$$f\in B_{s,p,q}\left(R^{d}\right)$$

if and only if $f\in W_{p}^{m}\left(R^{d}\right)$ and every weak derivative $D^{j}f$ of order |j|=m belongs to $B_{\sigma ,p,q}\left(R^{d}\right)$, with equivalence of the corresponding norms. At integer smoothness, the highest-order regularity is of Zygmund type and is expressed through higher-order differences rather than by setting the fractional part equal to one without qualification.

The Besov scale contains a number of classical spaces. In particular,

$$H^{s}\left(R^{d}\right)=B_{s,2,2}\left(R^{d}\right)$$

with equivalence of norms, while $B_{s,\infty ,\infty }\left(R^{d}\right)$ is the Hölder–Zygmund class. For non-integer s>0, this space agrees with the classical Hölder class under the usual identification. The spaces used in this paper are isotropic Besov spaces, for which the same smoothness index applies in every coordinate direction. While they accommodate spatially inhomogeneous and locally irregular behavior, anisotropic regularity requires a genuinely anisotropic Besov scale with direction-dependent smoothness indices, and is not covered by the present notation. The wavelet characterization supplies the approximation results needed in the main proofs. Let the multiresolution analysis be *r*-regular and let 0<s<r. If

$$f=\sum_{k\in Z^{d}}a_{0,k}\phi_{0,k}+\sum_{j\geq 0}\sum_{\ell =1}^{2^{d}-1}\sum_{k\in Z^{d}}b_{\ell ,j,k}\psi_{\ell ,j,k}$$

in the appropriate distributional sense, then $f\in B_{s,p,q}\left(R^{d}\right)$ if and only if

$$∥a_{0,\cdot }{∥}_{\ell_{p}}+{\left(\sum_{j\geq 0}{\left[2^{j\{s+d(1/2-1/p)\}}{∥b_{j,\cdot }∥}_{\ell_{p}}\right]}^{q}\right)}^{1/q}<\infty ,$$

with the standard modification when p=∞ or q=∞. This equivalence is the precise bridge between analytic smoothness and multiresolution approximation. For the $L_{2}$-risk bound used in Theorem 1, the relevant specialization is

$$f\in B_{s,2,q}\left(R^{d}\right).$$

In that case,

$$∥f-P_{V_{m}}{f∥}_{L_{2}}\leq C2^{-ms}{∥f∥}_{B_{s,2,q}},$$

and therefore

$$∥f-P_{V_{m}}{f∥}_{L_{2}}^{2}\leq C2^{-2ms}{∥f∥}_{B_{s,2,q}}^{2}.$$

This is the deterministic term appearing in (38) and (39). No derivative correction is to be subtracted from *s* at this stage, because the function being projected is already the target derivative $\left(\partial^{\beta }r\right)\left(\rho ;\cdot \right)$. For uniform approximation, the embedding condition is dimension dependent. If

$$s>\frac{d}{p},$$

then

$$B_{s,p,q}\left(R^{d}\right)↪C_{b}\left(R^{d}\right)$$

under the standard restrictions on the fine index at the critical boundary, and the wavelet projection satisfies a bound of the form

$$∥f-P_{V_{m}}{f∥}_{L_{\infty }}\leq C2^{-m(s-d/p)}{∥f∥}_{B_{s,p,q}}.$$

Thus, whenever the proofs invoke boundedness or uniform projection convergence through a Besov embedding, the condition s>d/p, rather than s>1/p, is the relevant hypothesis in dimension *d*.

**Remark** **11.** *The minimax $L_{2}$-risk over an isotropic d-dimensional Sobolev or Besov ball has the classical order*

$$n^{-2s/(2s+d)}$$

*for direct density estimation under the standard independent model. The rate*

$$n^{-2s/(2s+1)}$$

*is the special case d=1; see [51]. This benchmark is not the rate proved in the present dependent derivative estimation problem, for which the stochastic exponent is altered by derivative order, dimension, inverse weighting, and the conditional density stabilization bound. Detailed accounts of the relationships among Besov, Sobolev, Hölder, and related smoothness classes may be found in [47,52]. Connections between Besov spaces and $V_{p}$-type spaces of functions of bounded p-variation are developed in [53] using interpolation-theoretic tools from [54]; a classical treatment of p-variation is given in [55]. Extensions of Besov theory to more general geometric and analytic settings, including manifolds and Dirichlet spaces, are discussed in [56].*

## Figures and Tables

**Table 1:** Empirical verification of stationarity and ergodicity for DGP1–DGP4 (n=20,000): split-sample mean/variance stability and Cesàro-averaged autocorrelation decay.

DGP	Mean (1st Half)	Mean (2nd Half)	Var (1st Half)	Var (2nd Half)	ACF (1)	ACF (10)	ACF (20)
AR1	0.008	−0.007	0.994	0.984	0.596	0.022	0.005
NLAR	0.008	0.000	0.255	0.257	0.718	0.034	0.010
MARKOV	0.071	0.010	1.883	1.885	0.698	0.046	−0.005
GARCH	−0.001	0.014	0.973	0.990	0.005	−0.006	0.014

**Table 2:** Pointwise Bias, Variance and MSE at n=500, 30% missingness (polynomial regression function).

*x*	Estimator	Bias	Variance	MSE
−1.40	Oracle IPW	−0.362	1.562	1.694
	Feasible IPW	−0.538	0.594	0.883
	Complete-case	−1.622	0.699	3.331
	IPW kernel	0.007	0.024	0.024
−1.05	Oracle IPW	−0.247	0.318	0.379
	Feasible IPW	−0.352	0.167	0.291
	Complete-case	−1.039	0.175	1.254
	IPW kernel	0.011	0.013	0.013
−0.70	Oracle IPW	−0.174	0.099	0.129
	Feasible IPW	−0.248	0.054	0.115
	Complete-case	−0.566	0.060	0.381
	IPW kernel	0.003	0.005	0.005
−0.35	Oracle IPW	−0.166	0.045	0.072
	Feasible IPW	−0.186	0.038	0.073
	Complete-case	−0.230	0.040	0.093
	IPW kernel	−0.010	0.005	0.005
0.00	Oracle IPW	−0.128	0.036	0.052
	Feasible IPW	−0.115	0.023	0.036
	Complete-case	0.046	0.038	0.040
	IPW kernel	−0.003	0.005	0.005
0.35	Oracle IPW	−0.060	0.058	0.062
	Feasible IPW	−0.043	0.033	0.035
	Complete-case	0.276	0.071	0.147
	IPW kernel	−0.004	0.004	0.004
0.70	Oracle IPW	−0.035	0.087	0.088
	Feasible IPW	−0.014	0.040	0.041
	Complete-case	0.387	0.114	0.264
	IPW kernel	0.007	0.006	0.006
1.05	Oracle IPW	0.019	0.130	0.130
	Feasible IPW	0.044	0.059	0.060
	Complete-case	0.468	0.187	0.406
	IPW kernel	0.019	0.005	0.005
1.40	Oracle IPW	0.036	0.187	0.188
	Feasible IPW	0.063	0.083	0.087
	Complete-case	0.450	0.288	0.490
	IPW kernel	−0.001	0.014	0.014

**Table 3:** Empirical MISE by sample size *n* and missingness rate: polynomial regression function (AR (1), ϕ=0.6).

Missing (%)	*n*	Oracle IPW	Feasible IPW	Complete-Case	IPW Kernel
10	100	0.446	0.361	0.491	0.046
	250	0.184	0.150	0.274	0.010
	500	0.113	0.090	0.189	0.006
	800	0.094	0.073	0.185	0.005
30	100	0.701	0.516	1.000	0.056
	250	0.418	0.285	0.785	0.014
	500	0.310	0.180	0.712	0.009
	800	0.219	0.132	0.631	0.007
50	100	1.514	1.008	1.927	0.144
	250	0.915	0.508	1.663	0.022
	500	0.673	0.333	1.390	0.014
	800	0.504	0.230	1.293	0.010

**Table 4:** Empirical MISE by sample size *n* and missingness rate: sinusoidal regression function (AR (1), ϕ=0.6).

Missing (%)	*n*	Oracle IPW	Feasible IPW	Complete-Case	IPW Kernel
10	100	0.322	0.317	0.316	0.140
	250	0.174	0.172	0.171	0.072
	500	0.116	0.115	0.116	0.051
	800	0.081	0.079	0.081	0.036
30	100	0.363	0.354	0.363	0.167
	250	0.200	0.186	0.209	0.080
	500	0.124	0.119	0.144	0.053
	800	0.091	0.087	0.115	0.038
50	100	0.430	0.399	0.444	0.220
	250	0.229	0.210	0.277	0.092
	500	0.166	0.145	0.229	0.056
	800	0.111	0.100	0.191	0.042

**Table 5:** MISE and mean CPU time per Monte Carlo replication as a function of *n* (polynomial regression function, AR (1), 30% missingness).

*n*	Oracle	Feasible	Complete-Case	IPW Kernel	CPU (s)
100	0.738	0.527	0.968	0.041	0.016
200	0.504	0.309	0.812	0.019	0.025
350	0.385	0.236	0.736	0.011	0.039
500	0.295	0.183	0.666	0.008	0.053
800	0.212	0.123	0.587	0.006	0.100
1200	0.185	0.105	0.586	0.005	0.197
Fitted rate: log(MISE)=a+blog(n), ${\hat{b}}_{\mathrm{oracle}}=-0.576$, ${\hat{b}}_{\mathrm{feasible}}=-0.655$					

**Table 6:** Average empirical coverage (nominal 95%) and CI length of the feasible IPW plug-in confidence interval.

Regression Fn.	Missing (%)	*n*	Coverage	CI Length
polynomial	10	100	0.584	0.725
	10	250	0.639	0.580
	10	500	0.644	0.497
	10	800	0.651	0.449
	30	100	0.560	0.807
	30	250	0.590	0.647
	30	500	0.611	0.567
	30	800	0.618	0.512
	50	100	0.547	0.966
	50	250	0.561	0.781
	50	500	0.576	0.677
	50	800	0.620	0.607
sinusoidal	10	100	0.222	0.328
	10	250	0.240	0.269
	10	500	0.247	0.227
	10	800	0.250	0.206
	30	100	0.231	0.370
	30	250	0.245	0.303
	30	500	0.273	0.258
	30	800	0.284	0.236
	50	100	0.261	0.447
	50	250	0.296	0.363
	50	500	0.298	0.310
	50	800	0.318	0.281

**Table 7:** Effect of undersmoothing on the coverage of the plug-in 95% confidence interval (n=500, 30% missingness, polynomial regression function; coverage and CI length averaged over the evaluation grid).

$h_{n}$ mult.	$h_{n}$	Mean Coverage	Mean CI Length	MISE (Feasible)
1.00	0.301	0.611	0.577	0.164
0.60	0.180	0.690	1.220	0.673
0.40	0.120	0.649	2.231	2.518

**Table 8:** Sensitivity to the wavelet bandwidth $h_{n}$ (n=500, 30% missingness, polynomial regression function).

Multiplier	$h_{n}$	MISE (Oracle)	MISE (Feasible)
0.50	0.150	1.489	1.195
0.75	0.225	0.614	0.380
1.00	0.301	0.264	0.159
1.50	0.451	0.232	0.203
2.00	0.601	0.314	0.340

**Table 9:** Sensitivity to the propensity-score bandwidth $\lambda_{n}$ (n=500, 30% missingness, polynomial regression function).

Multiplier	$\lambda_{n}$	MISE (Feasible)
0.50	0.150	0.132
0.75	0.225	0.149
1.00	0.301	0.151
1.50	0.451	0.245
2.00	0.601	0.309

**Table 10:** Sensitivity to the dependence strength (AR (1) parameter ϕ), n=500, 30% missingness, polynomial regression function.

ϕ	Oracle IPW	Feasible IPW	Complete-Case
0.2	0.258	0.163	0.661
0.5	0.332	0.186	0.704
0.8	0.281	0.198	0.705

**Table 11:** MISE of the four estimators across the four dependence structures (DGP1–DGP4), n=500, 30% missingness.

Process	Oracle IPW	Feasible IPW	Complete-Case	IPW Kernel
DGP1: Gaussian AR (1)	0.257	0.163	0.671	0.010
DGP2: Nonlinear AR (1)	0.265	0.159	0.359	0.033
DGP3: Markov chain	2.927	2.641	2.759	0.020
DGP4: GARCH(1,1)	0.312	0.181	0.663	0.009

**Table 12:** Simulation evidence produced in Section 5.

Referee Criticism	Simulation Evidence
(1) Finite-sample bias, variance, MSE, MISE, rates	Figure 1, Figure 2, Figure 3 and Figure 4; Table 2, Table 3, Table 4 and Table 5
(2) Asymptotic normality	Figure 6 and Figure 7 (QQ-plot, histogram/density, Shapiro–Wilk test)
(3) Confidence intervals (coverage, length)	Table 6 and Table 7; Figure 8, Figure 9 and Figure 10
(4) Comparison with competitors	All of Section 5.6 (oracle IPW, feasible IPW, complete-case, IPW local-polynomial kernel), Table 11
(5) Tuning-parameter sensitivity (resolution/bandwidth, propensity bandwidth, sample size, missingness rate)	Section 5.10: Table 8, Table 9 and Table 10, Figure 11, Figure 12 and Figure 13
(6) Robustness under stationary ergodic dependence	Section 5.11: Table 11, Figure 14, together with the stationarity/ergodicity verification of Table 1

## Data Availability

Data are contained within the article.
